# Supplementary material for: EnrichDO: a global weighted model for Disease Ontology enrichment analysis
Source: Gigascience. 2025 Mar 26;14:giaf021. doi: 10.1093/gigascience/giaf021 (PMC11945307; doi:10.1093/gigascience/giaf021)
Supplement: giaf021_Supplemental_File [file giaf021_supplemental_file.zip › Supplementary Materials.docx]

**Supplementary Materials for**

### **EnrichDO: a Global Weighted Model for Disease Ontology Enrichment Analysis**

Haixiu Yang^1,†,*^, Hongyu Fu^1,†^, Meiyi Zhang^1^, Yangyang Liu^1^, Yongqun Oliver He^3^, Chao Wang^1^, and Liang Cheng^1,2,*^

^1^ College of Bioinformatics Science and Technology, Harbin Medical University, Harbin, Heilongjiang, 150081, China.

^2^ National Health Commission (NHC) Key Laboratory of Molecular Probes and Targeted Diagnosis and Therapy, Harbin Medical University, Harbin, 150028, China.

^3^ University of Michigan Medical School, Ann Arbor, MI, USA.

^*^ Correspondence address. Liang Cheng, College of Bioinformatics Science and Technology, Harbin Medical University, Harbin, Heilongjiang, 150081, China. Email: [liangcheng@hrbmu.edu.cn](mailto:liangcheng@hrbmu.edu.cn); Haixiu Yang, College of Bioinformatics Science and Technology, Harbin Medical University, Harbin, Heilongjiang, 150081, China. Email: yanghaixiu@ems.hrbmu.edu.cn

^†^ Contributed equally.

Table 1. Comparison of enrichment results with other methods on ALL case.

| GOID | GOTerm | | level | | EnrichDO | | ORA | | topGO | |
| --- | --- | --- | --- | --- | --- | --- | --- | --- | --- | --- |
|  |  |  |  |  | p | rank | p | rank | p | rank |
| GO:0050851 | antigen receptor-mediated signaling pathway | 10 | | 3.24E-19 | | 1 | 2.35E-21 | 11 | 5.71E-03 | 91 |
| GO:0050852 | T cell receptor signaling pathway | 11 | | 5.03E-16 | | 2 | 1.03E-18 | 17 | 1.03E-18 | 1 |
| GO:0002429 | immune response-activating cell surface receptor signaling pathway | 9 | | 2.39E-14 | | 3 | 1.31E-20 | 12 | 1 | 1848 |
| GO:0002250 | adaptive immune response | 4 | | 1.87E-13 | | 4 | 5.35E-15 | 31 | 2.76E-10 | 6 |
| GO:0050870 | positive regulation of T cell activation | 9 | | 4.82E-13 | | 5 | 9.40E-14 | 39 | 1 | 8809 |
| GO:0002503 | peptide antigen assembly with MHC class II protein complex | 9 | | 1.05E-12 | | 6 | 1.05E-12 | 56 | 1.05E-12 | 2 |
| GO:0019886 | antigen processing and presentation of exogenous peptide antigen via MHC class II | 6 | | 1.40E-11 | | 7 | 1.40E-11 | 65 | 1 | 4697 |
| GO:0002768 | immune response-regulating cell surface receptor signaling pathway | 7 | | 1.59E-11 | | 8 | 1.19E-21 | 10 | 2.76E-02 | 241 |
| GO:0042113 | B cell activation | 6 | | 2.24E-11 | | 9 | 6.94E-13 | 52 | 2.40E-11 | 4 |
| GO:0009966 | regulation of signal transduction | 6 | | 3.74E-11 | | 10 | 1.17E-13 | 41 | 6.05E-01 | 958 |
| GO:0001775 | cell activation | 3 | | 2.38E-01 | | 1154 | 2.63E-29 | 1 | 5.57E-01 | 926 |
| GO:0045321 | leukocyte activation | 4 | | 2.01E-02 | | 199 | 7.01E-29 | 2 | 2.46E-01 | 717 |
| GO:0046649 | lymphocyte activation | 5 | | 3.06E-04 | | 59 | 2.81E-27 | 3 | 2.24E-07 | 11 |
| GO:0048583 | regulation of response to stimulus | 4 | | 1.48E-04 | | 51 | 1.60E-26 | 4 | 1 | 8571 |
| GO:0002684 | positive regulation of immune system process | 5 | | 3.60E-02 | | 276 | 6.85E-25 | 5 | 1 | 1985 |
| GO:0050776 | regulation of immune response | 5 | | 1.11E-06 | | 30 | 1.52E-24 | 6 | 1 | 8767 |
| GO:0050778 | positive regulation of immune response | 6 | | 1.50E-01 | | 860 | 1.60E-24 | 7 | 2.18E-04 | 26 |
| GO:0002682 | regulation of immune system process | 4 | | 3.51E-02 | | 273 | 2.19E-23 | 8 | 1 | 1984 |
| GO:0048584 | positive regulation of response to stimulus | 5 | | 2.54E-08 | | 17 | 2.41E-23 | 9 | 9.50E-01 | 1161 |
| GO:0002768 | immune response-regulating cell surface receptor signaling pathway | 7 | | 1.59E-11 | | 8 | 1.19E-21 | 10 | 2.76E-02 | 241 |
| GO:0019884 | antigen processing and presentation of exogenous antigen | 4 | | 5.37E-04 | | 65 | 1.59E-12 | 58 | 1.59E-12 | 3 |
| GO:0051251 | positive regulation of lymphocyte activation | 8 | | 4.99E-06 | | 37 | 3.56E-15 | 30 | 1.56E-10 | 5 |
| GO:0032663 | regulation of interleukin-2 production | 8 | | 1.77E-03 | | 97 | 1.25E-08 | 100 | 1.25E-08 | 7 |
| GO:0050853 | B cell receptor signaling pathway | 11 | | 1.43E-02 | | 170 | 7.53E-08 | 118 | 7.53E-08 | 8 |
| GO:0045061 | thymic T cell selection | 14 | | 1.28E-06 | | 32 | 8.17E-08 | 119 | 8.17E-08 | 9 |
| GO:0030098 | lymphocyte differentiation | 11 | | 1 | | 5584 | 2.46E-14 | 36 | 1.29E-07 | 10 |

* ORA represents the hypergeometric test.

Table 2. Hierarchical status of enrichment results with EnrichDO, ORA, and topGO on AD case.

|  | Top 50 | | | Top 100 | | | Top 200 | | |
| --- | --- | --- | --- | --- | --- | --- | --- | --- | --- |
|  | ORA | EnrichDO | topGO | ORA | EnrichDO | topGO | ORA | EnrichDO | topGO |
| level:2 | 3 |  |  | 3 |  |  | 5 | 2 | 1 |
| level:3 | 4 | 4 | 5 | 14 | 5 | 5 | 20 | 6 | 7 |
| level:4 | 8 | 4 | 3 | 19 | 11 | 7 | 38 | 21 | 13 |
| level:5 | 10 | 8 | 9 | 21 | 20 | 18 | 45 | 38 | 34 |
| level:6 | 12 | 17 | 17 | 24 | 29 | 32 | 45 | 57 | 62 |
| level:7 | 9 | 11 | 9 | 13 | 17 | 20 | 28 | 38 | 42 |
| level:8 | 4 | 6 | 7 | 6 | 16 | 13 | 17 | 30 | 28 |
| level:9 |  |  |  |  | 2 | 5 | 2 | 8 | 11 |
| level:10 |  |  |  |  |  |  |  |  | 2 |
| branch num | 21 | 31 | 49 | 43 | 68 | 98 | 90 | 131 | 190 |

* ORA represents the hypergeometric test.

Table 3. Enrichment results with different background of DOSE on AD case.

| DOID | DOTerm | Background with pc-genes | | | | Background with all-genes | | | |
| --- | --- | --- | --- | --- | --- | --- | --- | --- | --- |
|  |  | geneRatio | bgRatio | p-value | rank | geneRatio | bgRatio | p-value | rank |
| DOID:680 | tauopathy | 70/87 | 671/9106 | 2.55E-64 | 1 | 71/91 | 682/10312 | 1.01E-66 | 1 |
| DOID:10652 | Alzheimer's disease | 69/87 | 662/9106 | 5.29E-63 | 2 | 70/91 | 673/10312 | 2.05E-65 | 2 |
| DOID:1289 | neurodegenerative disease | 73/87 | 1356/9106 | 5.10E-47 | 3 | 74/91 | 1412/10312 | 2.57E-48 | 3 |
| DOID:331 | central nervous system disease | 77/87 | 2242/9106 | 1.34E-36 | 4 | 78/91 | 2318/10312 | 8.01E-38 | 4 |
| DOID:1307 | dementia | 33/87 | 194/9106 | 1.68E-33 | 5 | 33/91 | 201/10312 | 6.27E-34 | 5 |
| DOID:863 | nervous system disease | 77/87 | 3190/9106 | 2.86E-25 | 6 | 78/91 | 3308/10312 | 2.45E-26 | 6 |
| DOID:6713 | cerebrovascular disease | 32/87 | 330/9106 | 1.86E-24 | 7 | 32/91 | 337/10312 | 4.34E-25 | 7 |
| DOID:1561 | cognitive disorder | 46/87 | 1018/9106 | 8.13E-22 | 8 | 46/91 | 1031/10312 | 9.56E-23 | 8 |
| DOID:3454 | brain infarction | 25/87 | 215/9106 | 6.29E-21 | 9 | 25/91 | 220/10312 | 2.00E-21 | 9 |
| DOID:14330 | Parkinson's disease | 30/87 | 363/9106 | 7.92E-21 | 10 | 30/91 | 392/10312 | 1.01E-20 | 11 |
