# Supplementary figures and images for: EnrichDO: a global weighted model for Disease Ontology enrichment analysis
Source: Gigascience. 2025 Mar 26;14:giaf021. doi: 10.1093/gigascience/giaf021 (PMC11945307; doi:10.1093/gigascience/giaf021)

Performance with different threshold

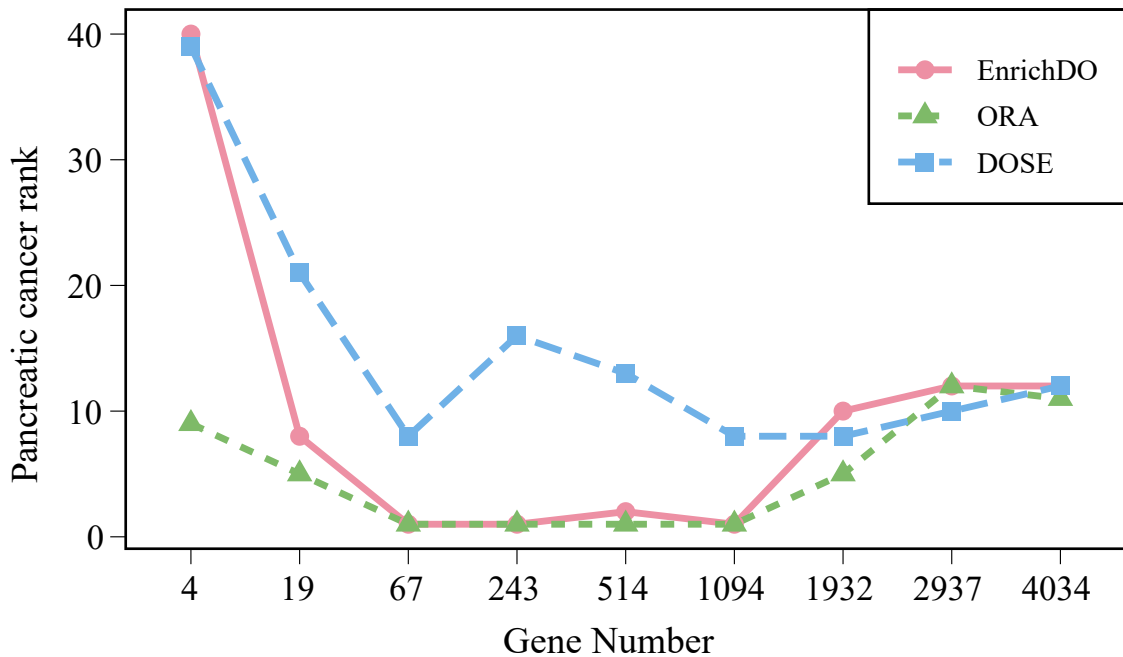

Supplement: giaf021_Supplemental_File [file giaf021_supplemental_file.zip › Figure S1_Supplementary Material.pdf]
