# Supplementary material for: EnrichDO: a global weighted model for Disease Ontology enrichment analysis
Source: Gigascience. 2025 Mar 26;14:giaf021. doi: 10.1093/gigascience/giaf021 (PMC11945307; doi:10.1093/gigascience/giaf021)

GigaScience

EnrichDO: a Global Weighted Model for Disease Ontology Enrichment Analysis

--Manuscript Draft--

**Manuscript Number:**	GIGA-D-24-00357
**Full Title:**	EnrichDO: a Global Weighted Model for Disease Ontology Enrichment Analysis
**Article Type:**	Technical Note
**Funding Information:**	Tou-Yan Innovation Team Program of the Heilongjiang Province (2019-15) — Dr. Liang Cheng<br>National Natural Science Foundation of China (62222104, 62172130) — Dr. Liang Cheng<br>National Natural Science Foundation of China (61902095) — Dr. Haixiu Yang<br>Heilongjiang Postdoctoral Science Foundation (LBH-Q20030) — Dr. Liang Cheng
**Abstract:**	Background: Disease Ontology (DO) has been widely studied in biomedical research and clinical practices to describe roles of genes, and DO enrichment analysis is an effective means to discover the associations between genes and diseases. Compared to hundreds of GO-based enrichment analysis methods, DO-based methods were relatively scare, and most current DO-based enrichment methods were term-for-term approach that unable to solve the over enriched problem caused by the “true-path” rule.<br>Results: We develop a double weighted model, EnrichDO, which is based on the latest annotations of the human genome with DO terms and integrates the DO graph topology on a global scale. Compared to classic enrichment methods (mainly for GO) and existing DO-based enrichment tools, EnrichDO performs better in both GO and DO enrichment analysis cases, it can accurately identify more specific terms without ignoring the truly associated parent terms, as shown in Alzheimer's disease (AD) case (AD ranked 1st). Moreover, the simulated test and data perturbation test validate the accuracy and the robustness of EnrichDO. Finally, EnrichDO is applied on various types of datasets to expand its application, such as gene expression profile datasets, host gene set of microorganisms, and hallmark gene sets, and shows significant improvement via all experimental results.<br>Conclusions: EnrichDO provides an effective DO enrichment analysis model to gain insights into the significance of a particular gene set in the context of diseases. To increase the usability of EnrichDO, we have developed an R-based software package, which is freely available through Bioconductor (https://www.bioconductor.org/packages/devel/bioc/html/EnrichDO.html) or at https://github.com/liangcheng-hrbmu/EnrichDO.
**Corresponding Author:**	Liang Cheng<br>Harbin Medical University<br>CHINA
**Corresponding Author Secondary Information:**	
**Corresponding Author's Institution:**	Harbin Medical University
**Corresponding Author's Secondary Institution:**	
**First Author:**	Haixiu Yang
**First Author Secondary Information:**	
**Order of Authors:**	Haixiu Yang<br>Hongyu Fu

Powered by Editorial Manager® and ProduXion Manager® from Aries Systems Corporation

	Meiyi Zhang
	Yangyang Liu
	Yongqun Oliver He
	Chao Wang
	Liang Cheng
**Order of Authors Secondary Information:**	
**Additional Information:**	
**Question**	**Response**
Are you submitting this manuscript to a special series or article collection?	No
**Experimental design and statistics**<br><br>Full details of the experimental design and statistical methods used should be given in the Methods section, as detailed in our Minimum Standards Reporting Checklist. Information essential to interpreting the data presented should be made available in the figure legends.<br><br>Have you included all the information requested in your manuscript?	Yes
**Resources**<br><br>A description of all resources used, including antibodies, cell lines, animals and software tools, with enough information to allow them to be uniquely identified, should be included in the Methods section. Authors are strongly encouraged to cite Research Resource Identifiers (RRIDs) for antibodies, model organisms and tools, where possible.<br><br>Have you included the information requested as detailed in our Minimum Standards Reporting Checklist?	Yes
**Availability of data and materials**<br><br>All datasets and code on which the conclusions of the paper rely must be	Yes

either included in your submission or deposited in publicly available repositories (where available and ethically appropriate), referencing such data using a unique identifier in the references and in the "Availability of Data and Materials" section of your manuscript.<br><br>Have you have met the above requirement as detailed in our Minimum Standards Reporting Checklist?	

Powered by Editorial Manager® and ProduXion Manager® from Aries Systems Corporation

Manuscript

Click here to access/download;Manuscript;EnrichDO-Manuscript.docx

EnrichDO: a Global Weighted Model for Disease Ontology Enrichment Analysis

Haixiu Yang[1,†,*], Hongyu Fu[1,†], Meiyi Zhang[1], Yangyang Liu[1], Yongqun Oliver He[3], Chao Wang[1], and Liang Cheng[1,2,*]

[1] College of Bioinformatics Science and Technology, Harbin Medical University, Harbin, Heilongjiang, 150081, China.

[2] National Health Commission (NHC) Key Laboratory of Molecular Probes and Targeted Diagnosis and Therapy, Harbin Medical University, Harbin, 150028, China.

[3] University of Michigan Medical School, Ann Arbor, MI, USA.

[*] Correspondence address. Liang Cheng, College of Bioinformatics Science and Technology, Harbin Medical University, Harbin, Heilongjiang, 150081, China. Email: liangcheng@hrbmu.edu.cn; Haixiu Yang, College of Bioinformatics Science and Technology, Harbin Medical University, Harbin, Heilongjiang, 150081, China. Email: yanghaixiu@ems.hrbmu.edu.cn

[†] Contributed equally.

ABSTRACT

Background: Disease Ontology (DO) has been widely studied in biomedical research and clinical practices to describe roles of genes, and DO enrichment analysis is an effective means to discover the associations between genes and diseases. Compared to hundreds of GO-based enrichment analysis methods, DO-based methods were relatively scare, and most current DO-based enrichment methods were term-for-term approach that unable to solve the over enriched problem caused by the “true-path” rule.

Results: We develop a double weighted model, EnrichDO, which is based on the latest annotations of the human genome with DO terms and integrates the DO graph topology on a global scale. Compared to classic enrichment methods (mainly for GO) and existing DO-based enrichment tools, EnrichDO performs better in both GO and DO enrichment analysis cases, it can accurately identify more specific terms without ignoring the truly associated parent terms, as shown in Alzheimer's disease (AD) case (AD ranked 1st). Moreover, the simulated test and data perturbation test validate the accuracy and the robustness of EnrichDO. Finally, EnrichDO is applied on various types of datasets to expand its application, such as gene expression profile datasets, host gene set of microorganisms, and hallmark gene sets, and shows significant improvement via all experimental results.

Conclusions: EnrichDO provides an effective DO enrichment analysis model to gain insights into the significance of a particular gene set in the context of diseases. To increase the usability of EnrichDO, we have developed an R-based software package, which is freely available through Bioconductor (https://www.bioconductor.org/packages/devel/bioc/html/EnrichDO.html) or at https://github.com/liangcheng-hrbmu/EnrichDO.

Keywords: Disease Ontology; human genome; enrichment analysis; double weighting

Key Points:

- EnrichDO presents a double weighted iterative model to address the over enriched problem caused by the "true-path" rule in DO enrichment analysis.
- Compared to the classic enrichment methods (mainly for GO) and the current DO-based enrichment tools, EnrichDO can accurately identify more specific terms without ignoring the truly associated parent terms.
- EnrichDO annotated the latest GeneRIF entries with current DO terms, which supplied a reliable data foundation for DO enrichment analysis.

Background

Biomedical ontologies organize biomedical findings into hierarchical structures and controlled vocabularies, which are human-readable and machine-computable. Biomedical ontologies have been widely studied and applied in biomedical research and clinical practice [1]. The Gene Ontology (GO) is arguably the most successful example of a biomedical ontology. It provides structured, controlled vocabularies and classifications that cover several domains of molecular and cellular biology and are freely available for community use in the annotation of genes, gene products and sequences [2, 3]. Besides, the Disease Ontology (DO) is also a presentative example, which organizes, represents, and standardizes human diseases through extensive cross-mapping and integration of MeSH, ICD, NCI's thesaurus, SNOMED CT, and OMIM disease-specific terms and identifiers [4, 5]. Other widely used biomedical ontologies, such as the Human Phenotype Ontology (HPO) [6, 7], Chemical Entities of Biological Interest (ChEBI) [8, 9] and the Ontology of Adverse Events (OAE) [10] have been developed, and included in the BioPortal [11, 12] and/or the OBO Foundry [13, 14]. With the development of biomedical ontologies, massive excellent algorithms, tools and platforms have emerged, including annotation, similarity calculation, enrichment analysis and function prediction. For example, AmiGO [15] and GO-CAM [16] enable functional annotations of genes and gene products, GOSemSim provides an R package for measuring semantic similarity among GO terms and gene products [17], PANTHER presents a web service for GO enrichment analysis [18], and PhenIX effectively diagnoses genetic diseases through computational phenotype analysis of disease-associated genomes [19]. However, most of state-of-the-art methods were focused on GO, and DO-based analysis mainly focus on similarity calculation [20-22].

Biomedical ontology-based enrichment analysis can help to elucidate the potential biological significance of a particular set of genes, such as differentially expressed gene lists of high-throughput experiments. Thousands of studies have been conducted by GO-based enrichment, but DO-based enrichment methods were relatively scare. Osborne et al. used the Unified Medical Language System (UMLS) MetaMap Transfer tool (MMTx) and the Gene

Reference Into Function (GeneRIF) database to annotate the human genome with DO for the first time [23]. LePendu et al. annotated the GO annotation files with DO terms by the NCBO annotator, and enabled an enrichment analysis with DO using a simple binomial model [24]. The disease and gene annotations database (DGA) semantically annotates human genes with disease descriptors by using NCBO Annotator service and GeneRIF [25]. Based on the annotations of the human genome with DO, DO-based enrichment analysis has appeared to discover disease associations from high-throughput biological data. For example, KOBAS 2.0 has incorporated 5 human disease databases, and provided a web server for annotation and identification of enriched diseases by binomial test and FDR correction [26]. DOSim [20] and DOSE [27] were widely used R packages for enrichment analysis which using the hypergeometric model and gene set enrichment analysis. Based on high-quality curated database holding gene expression profiles and diseases, ADEPTUS provided a tool that enable various functional genomics analyses, including DO enrichment analysis [28].

Obviously, DO-based annotation and enrichment is relative scare, and current DO enrichment methods are lacking. On the one hand, gene-DO annotations and DO terms of the current methods have not been updated, and some web services cannot be accessed due to lack of maintenance. On the other hand, these methods were based on classic models, such as hypergeometric test, Fisher's exact test, χ^2-test and binomial test, which do not take dependencies between DO terms into consideration. DO has a hierarchical structure that forms a DAG that follows the "true-path" rule, which means if a gene is annotated to node t, it is also annotated to all parent terms of t. This inheritance problem can lead to some terms being over enriched, which has been well addressed in GO-based enrichment methods [29, 30].

In this study, we annotated the human genome with DO based on the latest data source, and developed a novel DO enrichment method termed EnrichDO. The method tackled the "inheritance problem" moderately by considering the DO graph topology on a global scale, and double weighted the annotated genes. We compared

EnrichDO with classic enrichment methods and current DO-based enrichment tools, and evaluated the performance and robustness of EnrichDO. We also performed EnrichDO on datasets directly related to diseases (including a microarray gene-expression dataset and an RNA-seq expression dataset), as well as the datasets indirectly related to diseases (including a microorganisms co-expressed host gene set and biological processes-related datasets). Our results showed that EnrichDO outperformed classic methods and current DO-based enrichment tools, demonstrating EnrichDO as an effective tool to gain insights into the significance of a particular set of genes in the context of diseases. EnrichDO has been implemented as an R-based tool, which is available at https://www.bioconductor.org/packages/devel/bioc/html/EnrichDO.html or https://github.com/liangcheng-hrbmu/EnrichDO.

Materials and Methods

Data Collection and Pre-processing

The standard DO terms were downloaded from the DO Database (http://disease-ontology.org) (data-version: releases/2024-03-28/doid.obo). The DO database represents a comprehensive knowledgebase aim to organize, represent, and standardize human diseases. The DO semantically integrates disease and medical vocabularies through extensive cross-mapping of DO terms to MeSH, ICD, NCI's thesaurus, SNOMED CT and OMIM disease-specific terms and identifiers [4, 5]. The DO has been widely used for disease annotation by various biomedical databases [5]. The obo file records the information of 14,019 DO terms, and we collected the information of 11,537 DO terms excluding the obsolete DO terms (is_obsolete=true). The names and the synonyms of DO terms, were extracted to construct the dictionary of disease names for DO annotation. The DOID and is_a relations were used to construct a directed acyclic graph (DAG) of DO.

The information of human genes with disease description was downloaded from the GeneRIF (version date:31/3/2024). GeneRIF is a database that provides concise description of gene functions, making it the most

suitable resource for DO annotation to infer gene-disease associations [23]. GeneRIF is available from the NCBI Gene Database (ftp://ftp.ncbi.nih.gov/gene/GeneRIF/). GeneRIF entries were manually extracted from scientific literature, including Tax IDs, Gene IDs, PubMed IDs, and concise GeneRIF textual descriptions (up to 250 characters) of gene function. The GeneRIF entries of the *Homo sapiens* were extracted from the generifs_basic file, then GeneRIF text was annotated with DO terms.

Annotating the Human Genome with DO Terms

Semantically annotating the human genome with DO terms can connect biomedical gene and disease data through the lens of human disease. In this study, the textual descriptions of gene function in the GeneRIF database were annotated with DO terms using the University of Michigan's Mgrep tool. The Mgrep is an efficient tool for mapping free text to ontology terms [31], which is used in the concept recognition step by NCBO Annotator service [11]. We choose Mgrep because it is claimed to be a fast and scalable tool for concept recognition with a high degree of customizability vis-à-vis dictionaries and resources [32]. For concept recognition, the data resources represent a particular type of biomedical knowledge, a dictionary represents a set of terms to recognize in the biomedical data resource. In our study, the biomedical data resources are GeneRIF entries of the *Homo sapiens*, the disease dictionary was constructed by extracting all standard DO terms and their synonyms. The concept recognizer recognizes the related disease name in the GeneRIF entries and maps it to the concept of DO terms in the dictionary. At last, 525,289 out of 1,039,774 GeneRIF entries of *Homo sapiens* were annotated with 4,407 disease terms.

The data pre-processing and result filtering process were conducted as follows: (i) the disease dictionary was generated to include all standard DO terms and synonyms, with character length no less than 3, and exclude the obsolete DO terms (is_obsolete=true); (ii) only GeneRIF entries of protein-coding genes (pc genes) of *Homo sapiens* were retained; (iii) the disease names annotated to DO terms and synonyms were standardized to DOID;

(vi) all repeating results were removed. As a result, we acquired 191,542 gene-DO annotations, which involved 4,361 DO terms and 15,106 pc genes. These DO annotations of human gene describe unique roles for genes in the context of diseases, and can be used for enrichment analysis with DO.

Semantic Expansion in DO DAG

We leveraged the hierarchical structure of the DO to expand annotations. Like GO, DO has a hierarchical structure that forms a DAG, which follows the "true-path" rule. The additional annotation information is produced using the semantic relationships in DO such as "is_a" relation as follows: firstly, DOID, name and "is_a" relations were used to construct the DAG. Every DO term is represented by a node in the graph, "is_a" relation is represented by an edge in the graph. Two terms with "is_a" relations are represented by a child node and parent node in the graph, and a child can have multiple parents. For example, "B-cell acute lymphoblastic leukemia" (DOID:0080638) is_a "acute lymphoblastic leukemia" (DOID:9952) and "lymphoma" (DOID:0060058), then the node "B-cell acute lymphoblastic leukemia" is a child of "acute lymphoblastic leukemia" and "lymphoma", and the child is a more specific biological classification than its parent. The term "Disease" (DOID:4) is the root node of the DAG, that has no parent, and is the most general node. Next, according to the gene-DO annotations mentioned above, a set of genes annotated with each DO term of DAG, were also annotated to its parent and its ancestor (the so-called true path rule). Leaf nodes with no annotated genes were iteratively pruned, until all nodes in the final DAG tree contained at least one annotated gene. After pruning, the DAG contained 4,813 nodes. At last, all nodes of DAG were marked as different levels. For a node n, the level was defined as the length of the longest path from the root to node n. The root node, the term "Disease", was marked as level 1, and level 13 is the highest level. Nodes in the same level do not share any edges, which can be investigated independently. The levels of DAG and the statistics of DO terms are displayed in Table 1.

Table 1. Annotations of molecules with ontology terms in EnrichDO.

Level	Num of DO	Num of Annotated Genes
1	1	15106
2	8	14400
3	120	15185
4	212	15526
5	574	15929
6	1242	15790
7	1009	13686
8	850	11600
9	535	7826
10	170	3231
11	58	1400
12	26	567
13	8	186

DO Enrichment based on Weighted Algorithm

Classical Enrichment Analysis

Overrepresentation analysis (ORA) is a widely used term-for-term approach for enrichment analysis. The ORA method measures the statistical proportion of a pre-selected list of genes of interest (e.g., differentially expressed gene list) and specific gene sets according to known gene functions (e.g. DO terms). Hypergeometric Test is a classical ORA test, the p-value indicating the probability of the null hypothesis, which can be calculated as follows:

$$p = 1 - \sum_{k=0}^{r-1} \frac{\binom{m}{k}\binom{N-m}{n-k}}{\binom{N}{n}} \qquad (1)$$

Where, N indicates the number of all human genes annotated to DO terms, n indicates the number of genes annotated to term t, m indicates the size of interesting gene list, of which r are included in the term t. A small p-value indicates a low probability of randomly obtaining such statistical proportion of interesting gene list and term t. Then multiple testing correction method such as Benjamini-Hochberg method was used to adjust the p-value to control the type I error (false positive) rate.

Weighted DO Enrichment Analysis

The disadvantage of the classical enrichment analysis approach is that it ignores the dependencies of annotations

between DO terms caused by "true-path" rule. And this inheritance problem can lead to some terms being over enriched. To address this problem, we developed the weighted DO Enrichment analysis method, referred to as EnrichDO, by double weighting the annotated genes and integrating DO graph topology on a global scale. On one hand, to reinforce the saliency of direct gene-DO annotations, we assigned different initial weights to directly annotated genes and indirectly annotated genes caused by the "true-path" rule, respectively. On the other hand, to detect locally most significant node between the parent and its children, we dynamically down-weighted genes in less significant nodes, as described by Alexa et al. [29].

We demonstrate how to set weights and how to calculate significance score with corresponding weights as follows.

(i) Setting initial weights w_i. Initially, for each DO term, the weights for all directly annotated genes are set to 1, and the weights for indirectly annotated genes lose weight by 0.1 for each level inherited upwards. The smaller the weights of indirectly annotated genes, the less contribution of these genes in the enrichment analysis. Notably, smaller weights can cause certain nodes being missed during the enrichment analysis. In this study, the weights for indirectly annotated genes were no less than 0.5.

(ii) Setting dynamic weights w_d. For a given DO term t and its child c, the weight assigned to genes annotated to this pair of terms is defined as follows:

$$w_d = \frac{\log(\text{score(c)})}{\log(\text{score(t)})} \qquad (2)$$

where score(.) is the p-value of hypergeometric test with weighted genes. The weights for genes annotated to each node are memorized and updated during the process.

(iii) Setting penalty score. The penalty score was used to alleviate the impact of different magnitudes of p-values, which defined as follows:

$$penal = \max\left(\frac{1}{10} \times \frac{\lg(xmin)}{\lg(score(t)) + \lg(score(c))}, 1\right) \qquad (3)$$

where $xmin$ was the min positive number in R, and score(.) is the p-value of hypergeometric test with weighted

genes mentioned above.

(iv) Calculating significance score. The significance score for a given DO term t is calculated by applying hypergeometric test with weighted genes mentioned above. The number r in formula (1), indicates the number of interesting genes included in the term t, is replaced by rounding down the sum of the weight of r genes. The number n, which indicates the number of genes annotated to term t is replaced by rounding down the sum of the weight of n genes.

Given a threshold of 0.01 (or less), our aim is to find significantly enriched DO terms with p-values <= 0.01. The main process is shown in Figure 1A, and the algorithm process is described step-by-step as follows:

Step 1. We process the nodes of the DAG bottom-up, from the highest level and then iteratively moves to nodes of lower level, with the aim to identify the most specific nodes with minimum required significance. For the current node t, all its children have significance scores, and the children with p-values larger than the threshold are excluded, shown in Figure 1B. Then the node t was processed by step 2 to step 5, as shown in Figure 1C.

Step 2. For the current node t, we calculate its significant score. If the score is larger than the threshold, then moves on to the next node. Otherwise, we compare t with each of its children to discover the most significant nodes locally. For each child c, we calculate the weight w_d of this pair of terms. $w_d > 1$ indicates that the child c has a more significant score than t, thus node c is a local optimum, we move c from children to sigChildren.

Step 3. For each child c in sigChildren, we down-weight the genes, that are annotated to c, from term t and all its ancestors by dividing them by w_d and penal, and removed c from the sigChildren, until the sigChildren is empty.

Step 4. Recursively execute Steps 2-3 with updated weights of t and compare it with its remaining children, to eliminate the significant children of t, until come with these two situations: (1) no remaining children, then moves on to the next node; (2) exist remaining children, then go to step 5.

Step 5. For each remaining child c in children, it is less significant than t, thus node t is a local optimum, and we

reduce the weight of c by multiplying w_d (w_d <1) and dividing it by the penal value, to reduce the contribution of gene to the child nodes, and recalculate its score to make it less significant, then moves on to the next node.

At last, the multiple testing correction procedure from Benjamini-Hochberg method (1995) is applied to adjust the significance score (p-values).

Results

Summary of Annotations of the Human Genome with DO

Annotating human genes with DO is a crucial work to discover gene-disease associations. In this study, GeneRIF and DO terms were utilized to annotate human genome with DO, by using the Michigan's Mrep tool. We annotated 525,289 GeneRIF entries of *Homo sapiens* with 4,407 disease ontologies directly. We then integrated and filtered the annotated results, and acquired 191,542 gene-DO annotations finally, which involved 4,361 DO terms and 15,106 genes. We analyzed all gene-DO annotations except for the term "disease" (DOID=4), because of its' lack of specific significance. The number of genes annotated with DO was distributed from 1 to 4,730. The annotation results present an uneven distribution, as shown in Figure 2A. 2,971 out of 4,360 DO terms (68.14%) only have less than 10 annotated genes, among which, 1,217 specific terms have only one annotated gene. In contrast, 29 DO terms have more than 1,000 annotated genes. The DO term "cancer" (DOID=162), with the highest number of 4,730 annotated genes, is a disease of cellular proliferation that is malignant and primary, characterized by uncontrolled cellular proliferation, local cell invasion and metastasis. It is a generalized disease lies in level 4 of DAG, which is widely studied. Other specific cancer types, such as breast cancer, hepatocellular carcinoma and colorectal cancer, are also widely studied, which annotated more than 3000 genes.

The number of DO terms annotated by gene was distributed from 1 to 506, which presented an uneven distribution, as shown in Figure 2B. 213 out of 13,699 genes (1.55%) were annotated to more than 100 DO terms, while 9,595 genes were annotated to no more than 10 DO terms, of which 2,463 genes annotated to only one DO term. The pc

gene "tumor necrosis factor (TNF)", annotated with the highest number of 506 DO terms, encodes a multifunctional proinflammatory cytokine that belongs to the TNF superfamily. This cytokine is involved in the regulation of a wide spectrum of biological processes including cell proliferation, differentiation, apoptosis, lipid metabolism, and coagulation. This cytokine has been implicated in a variety of diseases, including autoimmune diseases, insulin resistance, psoriasis, rheumatoid arthritis ankylosing spondylitis, tuberculosis, autosomal dominant polycystic kidney disease, and cancer. Mutations in this gene affect susceptibility to cerebral malaria, septic shock, and Alzheimer disease [33]. Other important genes, such as interleukin 6 (IL6), tumor protein p53 (TP53), vascular endothelial growth factor A (VEGFA), transforming growth factor beta 1 (TGFB1) and matrix metallopeptidase 9 (MMP9), are also implicated in a variety of diseases, which annotated more than 400 DO terms.

The accuracy of the annotations of EnrichDO was evaluated by comparing with the disease and gene annotations of DGA database [25], which was widely used for DO enrichment analysis [27]. We selected four diseases with annotated gene numbers in different distribution intervals, namely Leigh disease (DOID:3652, with 53 annotated genes), retinitis pigmentosa (DOID:10584, with 158 annotated genes), colorectal carcinoma (DOID:0080199, with 549 annotated genes) and schizophrenia (DOID:5419, with 1299 annotated genes). We then collected the manually curated disease-gene relations for these four diseases from the DisGeNET [34], MalaCards [35] and DISEASE [36] databases as standards. When comparing the directly annotated genes of Leigh disease with the DisGeNET, MalaCards and DISEASE database, we acquired 27, 11 and 26 overlaps, respectively, compared with 14, 7, and 13 overlaps of DGA (see Figure 2C). Other three diseases were all likewise. As Figure 2D-2F and Table 2 shown, the overlaps of the annotations of EnrichDO are all more than the DGA database, indicated the accuracy of the annotations of EnrichDO, which supply a reliable data foundation for DO enrichment analysis.

Table 2. Comparison of Annotations between EnrichDO and DGA for different DO Terms.

DOID	Disease	Num of related genes in EnrichDO and DGA	Database	Num of related genes	Num of overlaps with EnrichDO	Num of overlaps with DGA
DOID:3652	Leigh disease	53/21/15	MalaCards	23	11	7
			DISEASE	63	26	13
			DisGeNET	47	27	14
DOID:10548	retinitis pigmentosa	158/126/92	MalaCards	154	89	62
			DISEASE	106	77	62
			DisGeNET	104	80	57
DOID:0080199	colorectal carcinoma	549/52/15	MalaCards	97	26	8
			DISEASE	0	0	0
			DisGeNET	702	95	12
DOID:5419	schizophrenia	1299/720/665	MalaCards	30	12	13
			DISEASE	16	13	12
			DisGeNET	883	602	448

* The three values in the third column represent the number of genes annotated with corresponding DO term in EnrichDO and DGA, as well as the number of overlaps between EnrichDO and DGA.

Algorithm Comparison and Assessment

Comparison with Other Methods

We compared EnrichDO with classic overrepresentation analysis method (common hypergeometric test) and topGO [29]. These two methods were widely used in the GO enrichment analysis, thus we compared them based on both GO and DO, respectively.

We first applied three methods to implement GO (Biological Process, BP) enrichment analysis on acute lymphoblastic leukemia (ALL) case [37], which was studied in topGO previously. The dataset consists of 95 patients with B-cell ALL and 33 patients with T-cell ALL in the study. 194 differentially expressed pc genes with |logFC|>=1 were obtained to implemented the enrichment analysis. The top 10 enriched DO terms of EnrichDO were displayed in Figure 3A. Among top 10 result, EnrichDO had 4 terms overlapped with the topGO, including "T cell receptor signaling pathway" (GO:0050852), "adaptive immune response" (GO:0002250), "peptide antigen assembly with MHC class II protein complex" (GO:0002503) and "B cell activation" (GO:0042113). The top

enriched GO term of EnrichDO was “antigen receptor-mediated signaling pathway” (GO:0050851), which was defined the series of molecular signals initiated by the cross-linking of an antigen receptor on a B or T cell. The hypergeometric test had only one overlap with EnrichDO, named “immune response-regulating cell surface receptor signaling pathway” (GO:0002768). Besides, GO terms like “positive regulation of T cell activation” (GO:0050870), and “immune response-activating cell surface receptor signaling pathway” (GO:0002429) were also top ranked in EnrichDO’s results. As shown in Supplementary Table S1, the enriched terms of EnrichDO and topGO are more specific than the hypergeometric test. Notably, although the results of topGO are specific, they overlook the parent nodes of some significant nodes, and this is more evident in the following DO enrichment analysis.

We then implemented DO enrichment analysis on a publicly available datasets of Alzheimer’s disease (AD), which is a neurological disorders. Alzheimer’s disease causes a decline in thinking, memory, and language, personality changes, and certain changes in the brain, eventually results in the loss of ability to carry out the simplest daily tasks [38]. Due to the lack of a gold standard dataset for DO enrichment analysis, we collected AD related genes from the DisGeNET [34], which is deemed as gold standard dataset. We collected 91 curated disease associated pc genes from the DisGeNET, which were taken as interesting gene set for DO enrichment analysis. All three methods were based on the latest annotations of DO of EnrichDO.

The top 10 enriched DO terms of EnrichDO are presented in Figure 3B, with ascending order of their computed p-values. The first ranked disease is AD, indicating that EnrichDO can accurately enriched the specific disease. The second ranked DO term is “tauopathy” (DOID:680), which is a heterogeneous group of neurodegenerative diseases that all include aggregated tau proteins, and AD is a tauopathy that characterized by memory lapses, confusion, emotional instability and progressive loss of mental ability [39]. Besides, “mild cognitive impairment” (DOID:0080832) and “dementia” (DOID:1307) were also top ranked. Mild cognitive impairment (MCI) is an

intermediate state between normal cognition and dementia. MCI has proved to be a useful label in clinical settings to define people who are at risk of developing AD [40]. Dementia was characterized by progressive deterioration in cognition, function and behavior, and AD is the most frequent cause of dementia [40]. Apparently, top 10 ranked DO terms are all neurological or cognitive system diseases, indicating the accuracy of the enrichment algorithm. The comparison with the hypergeometric test and topGO on AD case is displayed in Figure 3B and Table 3. Among top 10 result, topGO had 7 overlaps with EnrichDO's results，the DO term "tauopathy" (2nd)，synucleinopathy (6th) were identified as non-significant terms (p-vlaue=1) by topGO, and cognitive disorder (7th) ranked 45th in the topGO results. Besides, "polycystic ovary syndrome", "type 2 diabetes mellitus" and "rheumatoid arthritis" were among top 10 results, which were more specific DO terms, but not so related to AD compared to the three terms mentioned above. Hypergeometric test had 8 overlapped DO terms with EnrichDO, and the top two enriched DO terms were "Alzheimer's disease" and "tauopathy", indicating the accuracy of our annotation results. Furthermore, DO terms ranked differently between two methods, of which top ranked DO terms of EnrichDO were more specific. The above results indicate that EnrichDO often yield more accuracy specific significant DO terms, which overcomes the over enriched problem caused by the "true-path" rule. At the same time, it is also a moderate weighted algorithm that will not neglect the true significant terms, even though their child nodes were significant.

Table 3. Comparison of enrichment results with other methods on AD case.

DOID	DOTerm	level	EnrichDO		ORA		topGO	
			p	rank	p	rank	p	rank
DOID:10652	Alzheimer's disease	7	2.19E-71	1	2.44E-71	1	2.44E-71	1
DOID:680	tauopathy	6	4.38E-63	2	3.89E-71	2	1	4054
DOID:0080832	mild cognitive impairment	4	3.09E-52	3	3.09E-52	4	3.09E-52	2
DOID:1307	dementia	4	3.02E-43	4	4.52E-44	5	4.52E-44	3
DOID:14330	Parkinson's disease	7	3.27E-42	5	3.48E-42	7	3.48E-42	4
DOID:0050890	synucleinopathy	6	2.26E-34	6	3.30E-41	8	1	3178

DOID:1561	cognitive disorder	3	1.26E-30	7	1.01E-43	6	5.72E-14	45
DOID:1596	depressive disorder	5	2.19E-28	8	1.40E-31	10	1.40E-31	5
DOID:2377	multiple sclerosis	8	8.99E-27	9	1.33E-27	12	1.33E-27	6
DOID:5419	schizophrenia	5	2.70E-25	10	2.78E-25	16	2.78E-25	7
DOID:1289	neurodegenerative disease	5	1.68E-22	15	8.17E-58	3	1	4348
DOID:150	disease of mental health	2	2.14E-11	107	3.61E-35	9	1	4810
DOID:1596	depressive disorder	5	2.19E-28	8	1.40E-31	10	1.40E-31	5
DOID:11612	polycystic ovary syndrome	7	3.05E-25	11	3.05E-25	17	3.05E-25	8
DOID:9352	type 2 diabetes mellitus	7	2.02E-22	16	2.07E-22	28	2.07E-22	9
DOID:7148	rheumatoid arthritis	8	2.96E-21	18	3.31E-21	33	3.31E-21	10

* ORA represent the hypergeometric test.

Comparison with Current DO-based Enrichment Analysis Tools

We also applied the AD case to compare EnrichDO with DOSE (v3.26.2) and KOBAS-i, which were current widely used DO-based enrichment analysis tools. All parameters of DOSE and KOBAS were default except for minGSSize and maxGSSize were set to 5 and 5000 for DOSE, respectively. Among top 10 results, KOBAS recognizes 5 duplicate AD-liked diseases, with different disease names which come from different human disease databases. The other results are mainly related to cardiovascular disease and metabolic disease. KOBAS incorporates 3 human disease databases, such as OMIM, KEGG DISEASE and NHGRI GWAS Catalog (NHGRI), but not include DO. Multiple data sources may lead to redundancy in the enrichment results. DOSE has 4 overlaps with EnrichDO, it recognizes “tauopathy” as the most significantly enriched DO term, which is the parent of “Alzheimer's disease”, and “Alzheimer's disease” ranked second. Besides, the parent and ancestors of the term “tauopathy”, such as “neurodegenerative disease”, “central nervous system disease” and “nervous system disease” were also significantly enriched, see Figure 4. This may be attributed to the inheritance problem caused by “true-path” rule, and DOSE identified statistically significantly enriched diseases based on hypergeometric test, which did not take topological relationships between DO terms into consideration. Another reason may be the annotation data of DOSE has not been updated, with only 10312 annotated genes, compared to 15106 genes in EnrichDO.

The above results indicate that EnrichDO performs better than current state-of-the-art methods.

Stability Assessment

We tested the stability of the EnrichDO with the AD dataset, by removing interesting genes and adding noise, respectively. Firstly, we removed the AD dataset from 5% to 30%, at 5% intervals, and repeated the EnrichDO method 100 times for each removal. The number of overlapped significant DO terms (top 100) fell slowly compared with the original data, and the ratio of overlapped DO terms to original significant DO terms remained 74.17%, even after removal of up to 30% of the AD dataset, as shown in Figure 5A. These results indicate that the EnrichDO method is robust to data removal. We also take a test by adding noises to AD dataset in the same way. We added 5% to 30% noise genes from the background gene set, at 5% intervals, repeated 100 times. The overlapped significant DO terms of top 100 fell to 94.8 to 86.6, see Figure 5B. The results indicated the robustness of EnrichDO to data noise.

Accuracy Assessment

The comparison and evaluation of different DO enrichment algorithms relies on the true relevant DO terms. On real datasets mentioned above, the true significant DO terms are not known. To address this problem, the simulated dataset was applied to test the accuracy of EnrichDO. The simulation study was designed as follows:

(i) Selected the known DO terms. The number of genes directly annotated with DO terms was distributed from 1 to 4,730. DO terms with annotated genes that are too small or too large are biased, therefore, we ordered DO terms by the number of their annotated genes, and select 20 (or 30) known DO terms randomly from the middle 50% DO terms, which were deemed as the truly enriched nodes.

(ii) Obtained the interesting gene list. The genes annotated to the known DO terms were combined to a single set, which were deemed as the list of interesting genes. Considering some genes annotated with many DO terms are without specificity, and may bring noise to simulation study, thus we remove these hub genes which were

annotated to 30 or more DO terms from the interesting gene list.

(iii) Evaluate the performance. In simulation study, the performance of the algorithms is evaluated by the number of overlaps between the identified significant DO terms and the known DO terms.

We compared the EnrichDO with the hypergeometric test (based on annotations of EnrichDO) and DOSE, as shown in Figure 5C-5F. When selecting 20 known DO terms, among top 25 results, EnrichDO identified an average of 9.23 (46.15%) nodes overlapped with known DO terms, compared with an average of 7.57(37.85%) overlaps for the hypergeometric test and 4.39 (21.95%) overlaps for DOSE, respectively. When relaxed to the top 100 nodes, an average of 14.79, 14.73 and 7.2 (73.95%, 73.65%, and 36%) overlaps was identified by EnrichDO, the hypergeometric test and DOSE, respectively (see Figure 5C). The results indicated that EnrichDO has better performance. Another simulating study of 30 selected known DO terms were also implemented in the same way, and the similar results were displayed in Figure 5D. Notably, when the interesting gene list was obtained by collecting genes directly annotated with 20 (or 30) known DO terms, the accuracy has significantly improved from 73.95% to 89.7% (or 57% to 72.93%), see Figure 5E and 5F. As shown here, the accuracy of EnrichDO was better than the hypergeometric test, indicating the weighted algorithm performs better than the classic ORA enrichment methods; the hypergeometric test was better than DOSE, indicating the accuracy of disease-gene annotations of EnrichDO is higher than that of DOSE.

EnrichDO Application on Disease Expression Profile Dataset

The EnrichDO was also applied on two real gene expression datasets of pancreatic cancer [41, 42]. The pancreatic cancer dataset I is the microarray gene-expression profiles (GSE16515), including 36 pancreatic tumor and 16 normal samples [41]. Briefly, a list of 1,380 differentially expressed genes (DEGs) were identified with a strict threshold ($|logFC|>=1$, $p\text{-value}<0.05$, $q\text{-value}<0.05$), of which 1,094 up-regulated DEGs as the interesting gene list. DO enrichment results using EnrichDO, DOSE and the hypergeometric test were displayed in Table 4 and

Figure 6A. EnrichDO identified "pancreatic cancer" (DOID:1793) as the most significant enriched term, while DOSE identified it as the 8th significant enriched term, the hypergeometric test identified it as the 1st significant enriched term. Besides, KOBAS identified it as 21rd, 24th significantly enriched terms. Among top 10 results, EnrichDO identified many cancer types, such as "stomach cancer" (DOID:76), "colorectal cancer" (DOID:9256), "breast cancer" (DOID:1612), and "lung non-small cell carcinoma" (DOID:3908). On the one hand, the interesting gene list contains many cancer genes, such as "ERBB2", "HMGA1", "MET", that related to these cancer types. On the other hand, these types of cancer have been widely studied and holds thousands of direct annotated genes, made them be apt to be enriched. Such case also took place in DOSE and the hypergeometric test results. Furthermore, DO terms identified by DOSE and the hypergeometric test were more general, e.g. "endocrine gland cancer" (DOID:170), "gastrointestinal system cancer" (DOID:3119) and "carcinoma" (DOID:305), which were ancestors of the results of EnrichDO. The above results indicated that EnrichDO performs better than other algorithms in real microarray gene-expression dataset.

Table 4. Comparison of enrichment analysis results of EnrichDO on PAAD case (GSE16515).

DOID	DOTerm	level	geneRatio	EnrichDO		ORA		DOSE	
				p	rank	p	rank	p	rank
DOID:1793	pancreatic cancer	6	435/1038	2.24E-92	1	4.64E-107	1	1.99E-24	8
DOID:10534	stomach cancer	6	498/1038	6.82E-90	2	3.67E-90	7	1.82E-16	20
DOID:299	adenocarcinoma	6	488/1038	6.17E-89	3	1.04E-100	2	2.42E-10	59
DOID:1324	lung cancer	6	592/1038	4.03E-85	4	5.23E-89	9	1.86E-15	24
DOID:850	lung disease	5	646/1038	2.83E-80	5	1.20E-96	4	1.47E-12	44
DOID:9256	colorectal cancer	8	564/1038	2.34E-79	6	1.09E-79	15	3.43E-09	72
DOID:1612	breast cancer	6	613/1038	4.62E-79	7	2.18E-79	18	2.08E-25	6
DOID:3908	lung non-small cell carcinoma	8	481/1038	6.27E-79	8	2.68E-81	13	3.15E-14	28
DOID:76	stomach disease	4	501/1038	9.22E-79	9	1.11E-89	8	1.10E-03	241
DOID:0050615	respiratory system cancer	5	615/1038	1.97E-77	10	9.96E-91	6	5.30E-16	22
DOID:26	pancreas disease	4	445/1038	5.78E-68	16	7.90E-100	3	8.21E-03	376
DOID:28	endocrine system disease	3	617/1038	1.72E-32	48	3.05E-91	5	2.94E-02	495
DOID:170	endocrine gland cancer	5	633/1038	9.90E-50	24	5.71E-87	10	1.66E-27	4
DOID:0050687	cell type cancer	4	814/1038	1	4618	1	4674	1.66E-35	1

DOID:0050686	organ system cancer	4	944/1038	1	4617	1	4673	7.31E-32	2
DOID:305	carcinoma	5	792/1038	1	4497	1	4606	2.23E-30	3
DOID:170	endocrine gland cancer	5	633/1038	9.90E-50	24	5.71E-87	10	1.66E-27	4
DOID:3119	gastrointestinal system cancer	5	770/1038	1	4499	1	4608	2.44E-26	5
DOID:5093	thoracic cancer	5	613/1038	2.54E-71	13	2.40E-79	19	2.38E-25	7
DOID:120	female reproductive organ cancer	6	493/1038	3.07E-56	21	6.03E-75	21	2.62E-24	9
DOID:193	reproductive organ cancer	5	587/1038	1.14E-44	29	2.40E-71	24	1.00E-23	10

* ORA represent the hypergeometric test.

The pancreatic cancer dataset II is the RNA-seq expression profiles (GSE119794), including 10 paired pancreatic tumor and normal samples [42]. After the differential gene expression analysis, a list of 372 up-regulated DEGs were identified with a strict threshold (|logFC|>=1, p-value<0.05, q-value<0.05), which were adopted as interesting gene list. As Figure 6B displayed, EnrichDO obtained similar enrichment results to dataset I, even though the number of interesting genes varied significantly. Among top 10 results, the terms identified by EnrichDO were more specified than the hypergeometric test, though "Pancreatic cancer" ranked 2nd in the EnrichDO results, indicating that EnrichDO's utility on RNA-seq expression dataset. In contrast, DOSE identified "pancreatic cancer" as the 44th significantly enriched term. The results show that the annotations and proposed algorithms perform better than the classic methods on real gene expression datasets.

EnrichDO Application on Other Datasets

DO Enrichment on Host Gene Set of Microbes

To test the application of EnrichDO on other datasets, we applied EnrichDO on host gene set of microbes of Inflammatory Bowel Disease (IBD). IBD, mainly in forms of Crohn's disease (CD) and ulcerative colitis (UC), are characterized by debilitating and chronic relapsing and remitting inflammation of the gastrointestinal tract or the colon, exhibits heterogeneity at the clinical, molecular, genetic, and microbial levels [43]. Sambhawa et al. investigated the shared and disease-specific host gene-microbiome associations and identified subsets of

significantly correlated host genes and gut microbes [44]. The set of host genes were used to perform DO enrichment analysis in this study. EnrichDO identified the IBD (DOID:0050589) accurately, with rank 1st in the result list, and "colorectal cancer" (DOID:9256) were ranked 3rd. Besides, the second term was "asthma" (DOID:2841), which was found associated with subsequent Development of IBD by Kuenzig et al. [45]. As shown in Figure 6C, the result indicated the accuracy of EnrichDO when using microorganisms co-expressed host genes, conforming the hypothesis that host genes and gut microbial taxa involved in common biological functions would act in a coordinated fashion.

DO Enrichment on Hallmark Gene Sets

The EnrichDO can also be applied to study the potential connections between diseases and the specific gene sets, such as hallmark gene sets. We selected the hallmark gene sets of "inflammatory response" and "pancreas beta cells" from the Molecular Signatures Database (MSigDB) [46], and used the gene sets to execute DO enrichment analysis. For "inflammatory response", top 10 enriched DO terms in EnrichDO were Inflammation-related diseases or immune diseases (see Table 5 and Figure 6D). For "pancreas beta cells", tope 10 enriched DO terms in EnrichDO were all islet-related diseases or diabetes mellitus (see Table 6 and Figure 6E). The results suggested the EnrichDO is an effective mean to uncover the associations between the specified gene sets and the human diseases.

Table 5. The top 10 enrichment analysis results of EnrichDO on inflammatory response case.

DOID	DOTerm	p	p.adjust	geneRatio	bgRatio
DOID:0060032	autoimmune disease of musculoskeletal system	5.04E-56	2.43E-52	129/200	2083/15106
DOID:7148	rheumatoid arthritis	4.59E-52	1.11E-48	99/200	1235/15106
DOID:848	arthritis	1.43E-45	2.30E-42	110/200	1735/15106
DOID:2841	asthma	2.96E-40	3.56E-37	76/200	905/15106
DOID:3342	bone inflammation disease	4.70E-40	4.52E-37	112/200	1819/15106
DOID:9074	systemic lupus erythematosus	8.41E-39	6.75E-36	69/200	780/15106
DOID:3393	coronary artery disease	2.96E-38	2.03E-35	82/200	1161/15106

DOID:865	vasculitis	1.95E-37	1.17E-34	53/200	385/15106
DOID:417	autoimmune disease	1.64E-36	8.76E-34	144/200	2877/15106
DOID:1176	bronchial disease	5.42E-36	2.61E-33	79/200	952/15106

Table 6. The top 10 enrichment analysis results of EnrichDO on pancreas beta cells case.

DOID	DOTerm	p	p.adjust	geneRatio	bgRatio
DOID:11717	neonatal diabetes	5.71E-18	2.75E-14	9/39	27/15106
DOID:9351	diabetes mellitus	2.75E-15	6.61E-12	28/39	2087/15106
DOID:0050524	maturity-onset diabetes of the young	2.59E-13	4.15E-10	11/39	101/15106
DOID:10603	glucose intolerance	1.90E-12	2.28E-09	9/39	95/15106
DOID:9352	type 2 diabetes mellitus	2.44E-10	2.35E-07	22/39	1449/15106
DOID:3892	insulinoma	5.14E-10	4.13E-07	6/39	38/15106
DOID:4195	hyperglycemia	1.01E-09	6.92E-07	13/39	324/15106
DOID:0111102	maturity-onset diabetes of the young type 3	5.40E-08	3.25E-05	5/39	7/15106
DOID:2018	hyperinsulinism	6.95E-08	3.72E-05	6/39	84/15106
DOID:9993	hypoglycemia	9.20E-08	4.43E-05	6/39	89/15106

Discussion

Compared to various GO enrichment analysis methods, DO enrichment analysis methods are relatively scarce. We annotated the latest GeneRIF information with DO terms, and provided a new DO Enrichment analysis method, referred to as EnrichDO, which comprehensively considering the DO graph topology and double weighting the annotated genes. The EnrichDO was a moderate weighted algorithm that effectively addressed the over enriched problem without pruning the truly enriched nodes. EnrichDO improved the accuracy of enrichment analysis results compared to classic enrichment analysis methods and current DO-based enrichment analysis tools, and the simulation test and the stability test indicated that EnrichDO was robust. Additionally, we have also successfully applied the EnrichDO on multiple datasets, such as microarray gene-expression dataset, RNA-seq expression dataset, host gene set of microorganisms, and hallmark gene datasets, which expanded the application of EnrichDO.

There were also some limitations of our current study. One was that the size of interesting gene list influences the

enrichment analysis results. As shown in the simulation study, when the size of known DO terms has increased from 20 to 30, the average number of interesting genes (directly annotated) increased from 1525 to 2176 correspondingly, and the accuracy of EnrichDO decreases from 89.7% to 72.93% (see Figure 5E and 5F). It can be concluded that an excessively large set of interest genes can lead to a decrease of enrichment efficiency. This phenomenon also appears in the pancreatic cancer (GSE16515) case. By setting different thresholds of |logFC|, the number of interesting gene set has changed, and EnrichDO acquired the better performance when the number range 67-1094 (see Supplementary Figure S1). Besides, when the set of interest genes is quite large, some general DO terms with too many annotated genes tended to be enriched, such as "stomach cancer" and "lung non-small cell carcinoma", may be related to pancreatic cancer. It is worth noting that some top ranked terms, such as "stomach disease" (23/3141) and "lung disease" (119/4701), may be over enriched owing to inheritance problem, although EnrichDO down-weighted the indirectly annotated genes. Therefore, a moderate size of interesting gene list is suggested, and a threshold for the number of annotated gene of DO terms (e.g., minNum=10, maxNum=2000) is suggested. Another unexpected issue we observed is the bias of annotations. Some specific DO terms are with too few annotated genes to be enriched due to the lack of the relative research. 2,971 out of 4,361 DO terms were annotated to less than 10 genes. A pipeline that automatically integrates the manually curated disease-gene relations from other databases will greatly improve the performance of EnrichDO.

In general, the annotations of human genome with DO terms and the corresponding weighted DO Enrichment analysis method are superior the state-of-the-art methods. The semantically annotating results based on the latest GeneRIF and DO terms connected genes and diseases biomedical data through the lens of human disease. EnrichDO exhibits higher accuracy that often yield more specific significant DO terms, which alleviate the inheritance problem, makes it an effective DO enrichment tool.

Availability and Requirements

- Project name: EnrichDO
- Project home page: https://www.bioconductor.org/packages/devel/bioc/html/EnrichDO.html or https://github.com/liangcheng-hrbmu/EnrichDO.
- Operating system(s): Platform independent
- Programming language: R 3.5 or higher
- Other requirements: R packages BiocGenerics, Rgraphviz, clusterProfiler, hash, S4Vectors, dplyr, ggplot2, graph, magrittr, methods, pheatmap, purrr, readr, stringr, tidyr, grDevices, stats, RColorBrewer, knitr, rmarkdown, org.Hs.eg.db, testthat (>= 3.0.0)

Abbreviations

DO: Disease Ontology; AD: Alzheimer's disease; GO: Gene Ontology; HPO: Human Phenotype Ontology; ChEBI: Chemical Entities of Biological Interest; OAE: Ontology of Adverse Events; UMLS: Unified Medical Language System; MMTx: MetaMap Transfer tool; GeneRIF: Gene Reference Into Function; DGA: the disease and gene annotations database; DAG: directed acyclic graph; ORA: overrepresentation analysis; TNF: tumor necrosis factor;IL6: interleukin 6, TP53: tumor protein p53, VEGFA: vascular endothelial growth factor A, TGFB1: transforming growth factor beta 1; MMP9: matrix metallopeptidase 9; BP: Biological Process; ALL: acute lymphoblastic leukemia; MCI: Mild cognitive impairment; NHGRI: NHGRI GWAS Catalog; DEGs: differentially expressed genes; IBD: Inflammatory Bowel Disease; CD: Crohn's disease; UC: Ulcerative Colitis; MSigDB: Molecular Signatures Database.

Supplementary Material

Figure S1_Supplementary Material. Statistics of enrichment results according to different threshold of |logFC|.

Table S1_Supplementary Material. Comparison of enrichment analysis results with other methods on ALL case.

Authors' contributions

L.C. conceived the idea for the manuscript, H.Y. designed the study and generated annotations for the human genome with DO terms, H.Y. and H.F. wrote the source code, H.F. conducted testing and debugging of the model. H.F., M.Z., Y.L., and C.W. performed the case study analysis and implemented the data analytics. H.Y. and Y.O.H. wrote the manuscript. L.C. revised the manuscript. All authors read and approved the final manuscript.

Funding

This work was supported by Tou-Yan Innovation Team Program of the Heilongjiang Province [2019-15]; National Natural Science Foundation of China (grant numbers 62222104, 62172130 to LC, and 61902095 to HXY), and Heilongjiang Postdoctoral Fund (LBH-Q20030 to LC).

Data availability

Annotations of the Human Genome with DO were stored in https://github.com/liangcheng-hrbmu/EnrichDO/blame/devel/data/doterms.rda.

The datasets for case studies of enrichment analysis were collected as follows:

- The dataset of ALL case [37] was obtained from the R package from Bioconductor (https://www.bioconductor.org/packages/release/data/experiment/html/ALL.html), and the differentially expressed genes were extracted.
- The dataset of AD case was curated genes that downloaded from DisGeNET [34].
- The Expression Profile Datasets were downloaded from the Gene Expression Omnibus (GEO) database, namely GSE16515 and GSE119794 [41, 42], and the differentially expressed genes were extracted.
- The shared and disease-specific host gene-microbiome associations were obtained from the study of Priya [44].
- The Hallmark Gene Sets were downloaded from the Human Molecular Signatures Database [46] (MSigDB, https://www.gsea-msigdb.org/gsea/msigdb/), including HALLMARK_PANCREAS_BETA_CELLS and

HALLMARK_INFLAMMATORY_RESPONSE.

Specific data information has been uploaded to https://github.com/liangcheng-hrbmu/EnrichDO/tree/devel/thesisData. An archival copy of the code is available via Software Heritage and the code has been registered in SciCrunch (https://rrid.site/about/registry/SCR_025840) and bio.tools..

Competing Interests

The authors declare that they have no competing interests.

Figure Titles and Legends

Figure 1. Flowchart of EnrichDO. (A) The main process of EnrichDO. (B) The nodes of the DAG were processed bottom-up, and the children of node t were divided into different sets according to their significance scores. (C) Each node t was processed in detail step by step.

Figure 2. Statistics and Comparisons of Annotations of the Human Genome with DO. (A) Statistics of genes annotated with each DO term. (B) Statistics of DO terms annotated to each gene. (C) Annotations of Leigh disease in different databases. (D) Annotations of retinitis pigmentosa in different databases. (E) Annotations of colorectal carcinoma in different databases. (F) Annotations of schizophrenia in different databases.

Figure 3. Comparison of EnrichDO with other methods. (A) Comparison of GO enrichment analysis of EnrichDO, topGO and the hypergeometric test, on ALL case. (B) Comparison of DO enrichment analysis of EnrichDO, topGO and the hypergeometric test, on AD case.

Figure 4. Comparison of Enrichment results of EnrichDO with DOSE tool on AD case.

Figure 5. Stability and Accuracy Assessment of EnrichDO. (A) (B) Stability assessment of EnrichDO by random deletion test and noise addition test on AD case. (C) (D) Accuracy assessment of EnrichDO with simulated dataset (semantic expansion genes) of 20 and 30 known DO terms. (E) (F) Accuracy assessment of EnrichDO with simulated dataset (directly annotated genes) of 20 and 30 known DO terms.

Figure 6. Enrichment results of various datasets. (A) Top 10 enrichment results of EnrichDO, ORA (hypergeometric test) and DOSE for microarray gene-expression profiles (GSE16515); (B) Top 10 enrichment results of EnrichDO, ORA (hypergeometric test) and DOSE for RNA-seq expression profiles (GSE119794); (C) Top 10 enrichment results of EnrichDO for host genes of microbes of IBD; (D) Top 10 enrichment results of EnrichDO for hallmark gene sets of "inflammatory response"; (E) Top 10 enrichment results of EnrichDO for hallmark gene sets of "pancreas beta cells".

References

1. Alterovitz G, Xiang M, Hill DP, Lomax J, Liu J, Cherkassky M, et al. Ontology engineering. Nat Biotechnol. 2010;28 2:128-30. doi:10.1038/nbt0210-128.
2. Ashburner M, Ball CA, Blake JA, Botstein D, Butler H, Cherry JM, et al. Gene ontology: tool for the unification of biology. The Gene Ontology Consortium. Nat Genet. 2000;25 1:25-9. doi:10.1038/75556.
3. Gene Ontology C, Aleksander SA, Balhoff J, Carbon S, Cherry JM, Drabkin HJ, et al. The Gene Ontology knowledgebase in 2023. Genetics. 2023;224 1 doi:10.1093/genetics/iyad031.
4. Schriml LM, Arze C, Nadendla S, Chang YW, Mazaitis M, Felix V, et al. Disease Ontology: a backbone for disease semantic integration. Nucleic Acids Res. 2012;40 Database issue:D940-6. doi:10.1093/nar/gkr972.
5. Baron JA, Johnson CS, Schor MA, Olley D, Nickel L, Felix V, et al. The DO-KB Knowledgebase: a 20-year journey developing the disease open science ecosystem. Nucleic Acids Res. 2024;52 D1:D1305-D14. doi:10.1093/nar/gkad1051.
6. Robinson PN, Kohler S, Bauer S, Seelow D, Horn D and Mundlos S. The Human Phenotype Ontology: a tool for annotating and analyzing human hereditary disease. Am J Hum Genet. 2008;83 5:610-5. doi:10.1016/j.ajhg.2008.09.017.
7. Gargano MA, Matentzoglu N, Coleman B, Addo-Lartey EB, Anagnostopoulos AV, Anderton J, et al. The Human Phenotype Ontology in 2024: phenotypes around the world. Nucleic Acids Res. 2024;52 D1:D1333-D46. doi:10.1093/nar/gkad1005.
8. Degtyarenko K, de Matos P, Ennis M, Hastings J, Zbinden M, McNaught A, et al. ChEBI: a database and ontology for chemical entities of biological interest. Nucleic Acids Res. 2008;36 Database issue:D344-50. doi:10.1093/nar/gkm791.
9. Hastings J, Owen G, Dekker A, Ennis M, Kale N, Muthukrishnan V, et al. ChEBI in 2016: Improved services and an expanding collection of metabolites. Nucleic Acids Res. 2016;44 D1:D1214-9. doi:10.1093/nar/gkv1031.
10. He Y, Sarntivijai S, Lin Y, Xiang Z, Guo A, Zhang S, et al. OAE: The Ontology of Adverse Events. J Biomed Semantics. 2014;5:29. doi:10.1186/2041-1480-5-29.
11. Noy NF, Shah NH, Whetzel PL, Dai B, Dorf M, Griffith N, et al. BioPortal: ontologies and integrated data resources at the click of a mouse. Nucleic Acids Res. 2009;37 Web Server issue:W170-3. doi:10.1093/nar/gkp440.
12. Whetzel PL, Noy NF, Shah NH, Alexander PR, Nyulas C, Tudorache T, et al. BioPortal: enhanced functionality via new Web services from the National Center for Biomedical Ontology to access and use ontologies in software applications. Nucleic Acids Res. 2011;39 Web Server issue:W541-5.

doi:10.1093/nar/gkr469.
13. Smith B, Ashburner M, Rosse C, Bard J, Bug W, Ceusters W, et al. The OBO Foundry: coordinated evolution of ontologies to support biomedical data integration. Nat Biotechnol. 2007;25 11:1251-5. doi:10.1038/nbt1346.
14. Jackson R, Matentzoglu N, Overton JA, Vita R, Balhoff JP, Buttigieg PL, et al. OBO Foundry in 2021: operationalizing open data principles to evaluate ontologies. Database (Oxford). 2021;2021 doi:10.1093/database/baab069.
15. Carbon S, Ireland A, Mungall CJ, Shu S, Marshall B, Lewis S, et al. AmiGO: online access to ontology and annotation data. Bioinformatics. 2009;25 2:288-9. doi:10.1093/bioinformatics/btn615.
16. Thomas PD, Hill DP, Mi H, Osumi-Sutherland D, Van Auken K, Carbon S, et al. Gene Ontology Causal Activity Modeling (GO-CAM) moves beyond GO annotations to structured descriptions of biological functions and systems. Nat Genet. 2019;51 10:1429-33. doi:10.1038/s41588-019-0500-1.
17. Yu G, Li F, Qin Y, Bo X, Wu Y and Wang S. GOSemSim: an R package for measuring semantic similarity among GO terms and gene products. Bioinformatics. 2010;26 7:976-8. doi:10.1093/bioinformatics/btq064.
18. Thomas PD, Ebert D, Muruganujan A, Mushayahama T, Albou LP and Mi H. PANTHER: Making genome-scale phylogenetics accessible to all. Protein Sci. 2022;31 1:8-22. doi:10.1002/pro.4218.
19. Zemojtel T, Kohler S, Mackenroth L, Jager M, Hecht J, Krawitz P, et al. Effective diagnosis of genetic disease by computational phenotype analysis of the disease-associated genome. Sci Transl Med. 2014;6 252:252ra123. doi:10.1126/scitranslmed.3009262.
20. Li J, Gong B, Chen X, Liu T, Wu C, Zhang F, et al. DOSim: an R package for similarity between diseases based on Disease Ontology. BMC Bioinformatics. 2011;12:266. doi:10.1186/1471-2105-12-266.
21. Cheng L, Li J, Ju P, Peng J and Wang Y. SemFunSim: a new method for measuring disease similarity by integrating semantic and gene functional association. PLoS One. 2014;9 6:e99415. doi:10.1371/journal.pone.0099415.
22. Hu Y, Zhao L, Liu Z, Ju H, Shi H, Xu P, et al. DisSetSim: an online system for calculating similarity between disease sets. J Biomed Semantics. 2017;8 Suppl 1:28. doi:10.1186/s13326-017-0140-2.
23. Osborne JD, Flatow J, Holko M, Lin SM, Kibbe WA, Zhu LJ, et al. Annotating the human genome with Disease Ontology. BMC Genomics. 2009;10 Suppl 1 Suppl 1:S6. doi:10.1186/1471-2164-10-s1-s6.
24. LePendu P, Musen MA and Shah NH. Enabling enrichment analysis with the Human Disease Ontology. J Biomed Inform. 2011;44 Suppl 1 Suppl 1:S31-s8. doi:10.1016/j.jbi.2011.04.007.
25. Peng K, Xu W, Zheng J, Huang K, Wang H, Tong J, et al. The Disease and Gene Annotations (DGA): an annotation resource for human disease. Nucleic Acids Res. 2013;41 Database issue:D553-60. doi:10.1093/nar/gks1244.
26. Xie C, Mao X, Huang J, Ding Y, Wu J, Dong S, et al. KOBAS 2.0: a web server for annotation and identification of enriched pathways and diseases. Nucleic Acids Res. 2011;39 Web Server issue:W316-22. doi:10.1093/nar/gkr483.
27. Yu G, Wang LG, Yan GR and He QY. DOSE: an R/Bioconductor package for disease ontology semantic and enrichment analysis. Bioinformatics. 2015;31 4:608-9. doi:10.1093/bioinformatics/btu684.
28. Amar D, Vizel A, Levy C and Shamir R. ADEPTUS: a discovery tool for disease prediction, enrichment and network analysis based on profiles from many diseases. Bioinformatics. 2018;34 11:1959-61. doi:10.1093/bioinformatics/bty027.
29. Alexa A, Rahnenfuhrer J and Lengauer T. Improved scoring of functional groups from gene expression data by decorrelating GO graph structure. Bioinformatics. 2006;22 13:1600-7.

doi:10.1093/bioinformatics/btl140.
30. Grossmann S, Bauer S, Robinson PN and Vingron M. Improved detection of overrepresentation of Gene-Ontology annotations with parent child analysis. Bioinformatics. 2007;23 22:3024-31. doi:10.1093/bioinformatics/btm440.
31. Dai M, Shah NH, Xuan W, Musen MA, Watson SJ, Athey BD, et al. An efficient solution for mapping free text to ontology terms. AMIA summit on translational bioinformatics. 2008;21.
32. Shah NH, Bhatia N, Jonquet C, Rubin D, Chiang AP and Musen MA. Comparison of concept recognizers for building the Open Biomedical Annotator. BMC Bioinformatics. 2009;10 Suppl 9 Suppl 9:S14. doi:10.1186/1471-2105-10-S9-S14.
33. Annibaldi A and Meier P. Checkpoints in TNF-Induced Cell Death: Implications in Inflammation and Cancer. Trends Mol Med. 2018;24 1:49-65. doi:10.1016/j.molmed.2017.11.002.
34. Pinero J, Ramirez-Anguita JM, Sauch-Pitarch J, Ronzano F, Centeno E, Sanz F, et al. The DisGeNET knowledge platform for disease genomics: 2019 update. Nucleic Acids Res. 2020;48 D1:D845-D55. doi:10.1093/nar/gkz1021.
35. Rappaport N, Twik M, Plaschkes I, Nudel R, Iny Stein T, Levitt J, et al. MalaCards: an amalgamated human disease compendium with diverse clinical and genetic annotation and structured search. Nucleic Acids Res. 2017;45 D1:D877-D87. doi:10.1093/nar/gkw1012.
36. Grissa D, Junge A, Oprea TI and Jensen LJ. Diseases 2.0: a weekly updated database of disease-gene associations from text mining and data integration. Database (Oxford). 2022;2022 doi:10.1093/database/baac019.
37. Chiaretti S, Li X, Gentleman R, Vitale A, Vignetti M, Mandelli F, et al. Gene expression profile of adult T-cell acute lymphocytic leukemia identifies distinct subsets of patients with different response to therapy and survival. Blood. 2004;103 7:2771-8. doi:10.1182/blood-2003-09-3243.
38. Ballard C, Gauthier S, Corbett A, Brayne C, Aarsland D and Jones E. Alzheimer's disease. Lancet. 2011;377 9770:1019-31. doi:10.1016/s0140-6736(10)61349-9.
39. Horie K, Barthélemy NR, Spina S, VandeVrede L, He Y, Paterson RW, et al. CSF tau microtubule-binding region identifies pathological changes in primary tauopathies. Nat Med. 2022;28 12:2547-54. doi:10.1038/s41591-022-02075-9.
40. Reitz C and Mayeux R. Alzheimer disease: epidemiology, diagnostic criteria, risk factors and biomarkers. Biochem Pharmacol. 2014;88 4:640-51. doi:10.1016/j.bcp.2013.12.024.
41. Pei H, Li L, Fridley BL, Jenkins GD, Kalari KR, Lingle W, et al. FKBP51 affects cancer cell response to chemotherapy by negatively regulating Akt. Cancer Cell. 2009;16 3:259-66. doi:10.1016/j.ccr.2009.07.016.
42. Lin J, Wu YJ, Liang X, Ji M, Ying HM, Wang XY, et al. Network-based integration of mRNA and miRNA profiles reveals new target genes involved in pancreatic cancer. Mol Carcinog. 2019;58 2:206-18. doi:10.1002/mc.22920.
43. Adolph TE and Zhang J. Diet fuelling inflammatory bowel diseases: preclinical and clinical concepts. Gut. 2022;71 12:2574-86. doi:10.1136/gutjnl-2021-326575.
44. Priya S, Burns MB, Ward T, Mars RAT, Adamowicz B, Lock EF, et al. Identification of shared and disease-specific host gene-microbiome associations across human diseases using multi-omic integration. Nat Microbiol. 2022;7 6:780-95. doi:10.1038/s41564-022-01121-z.
45. Kuenzig ME, Barnabe C, Seow CH, Eksteen B, Negron ME, Rezaie A, et al. Asthma Is Associated With Subsequent Development of Inflammatory Bowel Disease: A Population-based Case-Control Study. Clin Gastroenterol Hepatol. 2017;15 9:1405-12 e3. doi:10.1016/j.cgh.2017.02.042.

46. Castanza AS, Recla JM, Eby D, Thorvaldsdottir H, Bult CJ and Mesirov JP. Extending support for mouse data in the Molecular Signatures Database (MSigDB). Nat Methods. 2023;20 11:1619-20. doi:10.1038/s41592-023-02014-7.

Figure 1

Click here to access/download;Figure;Fig1.tiff

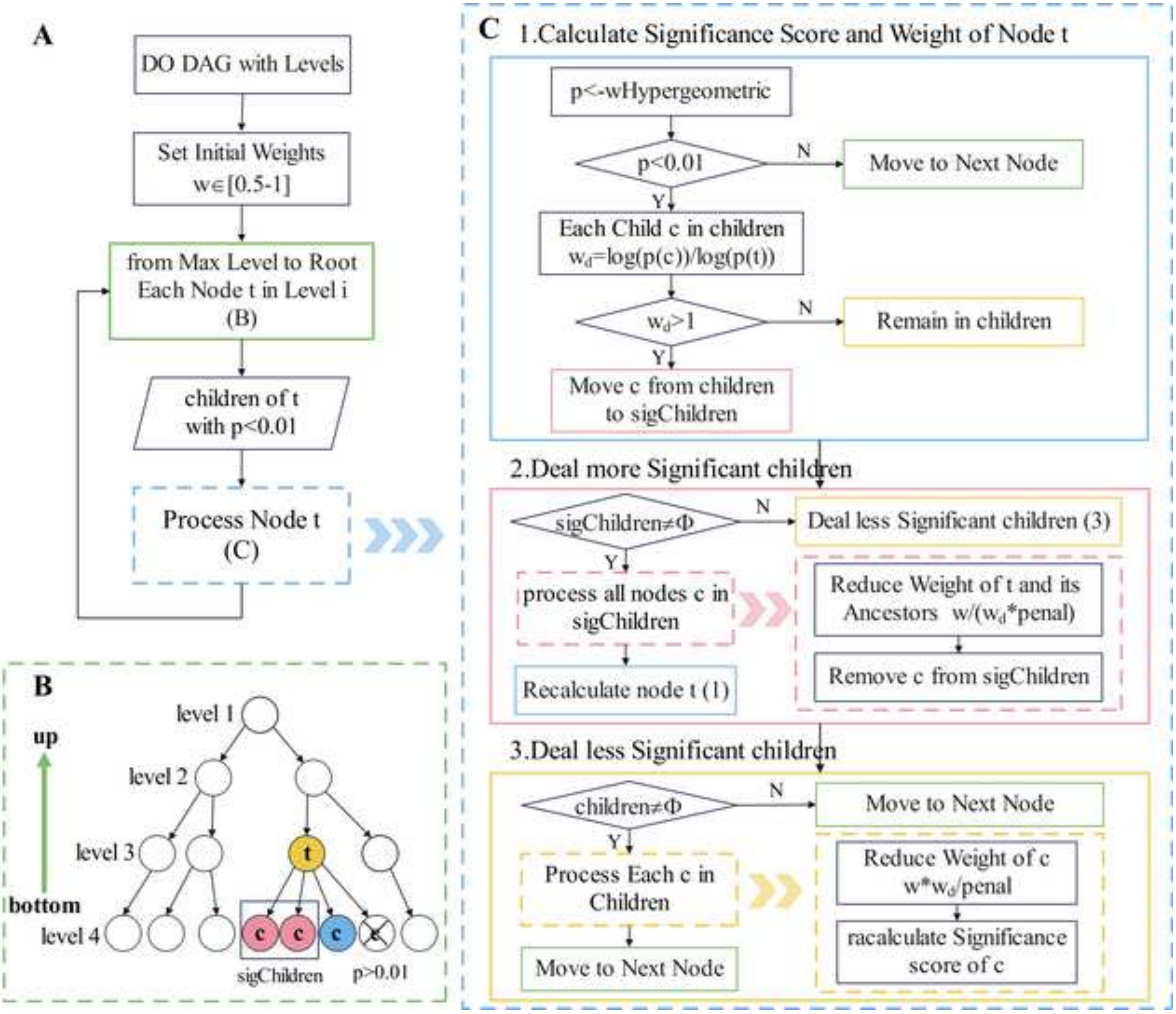

Figure 2

Click here to access/download;Figure;Fig2.tif

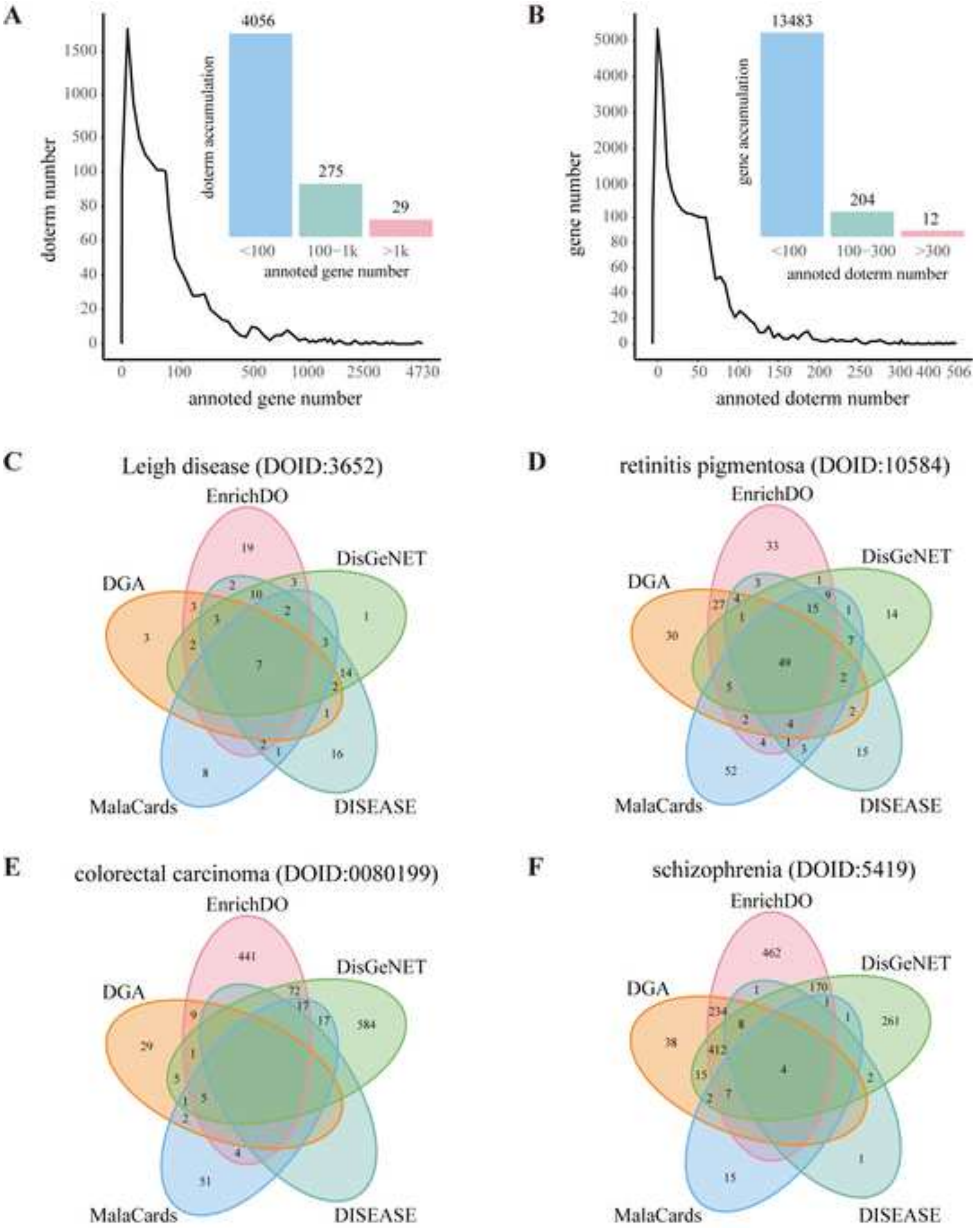

Figure 3

Click here to access/download;Figure;Fig3.tif

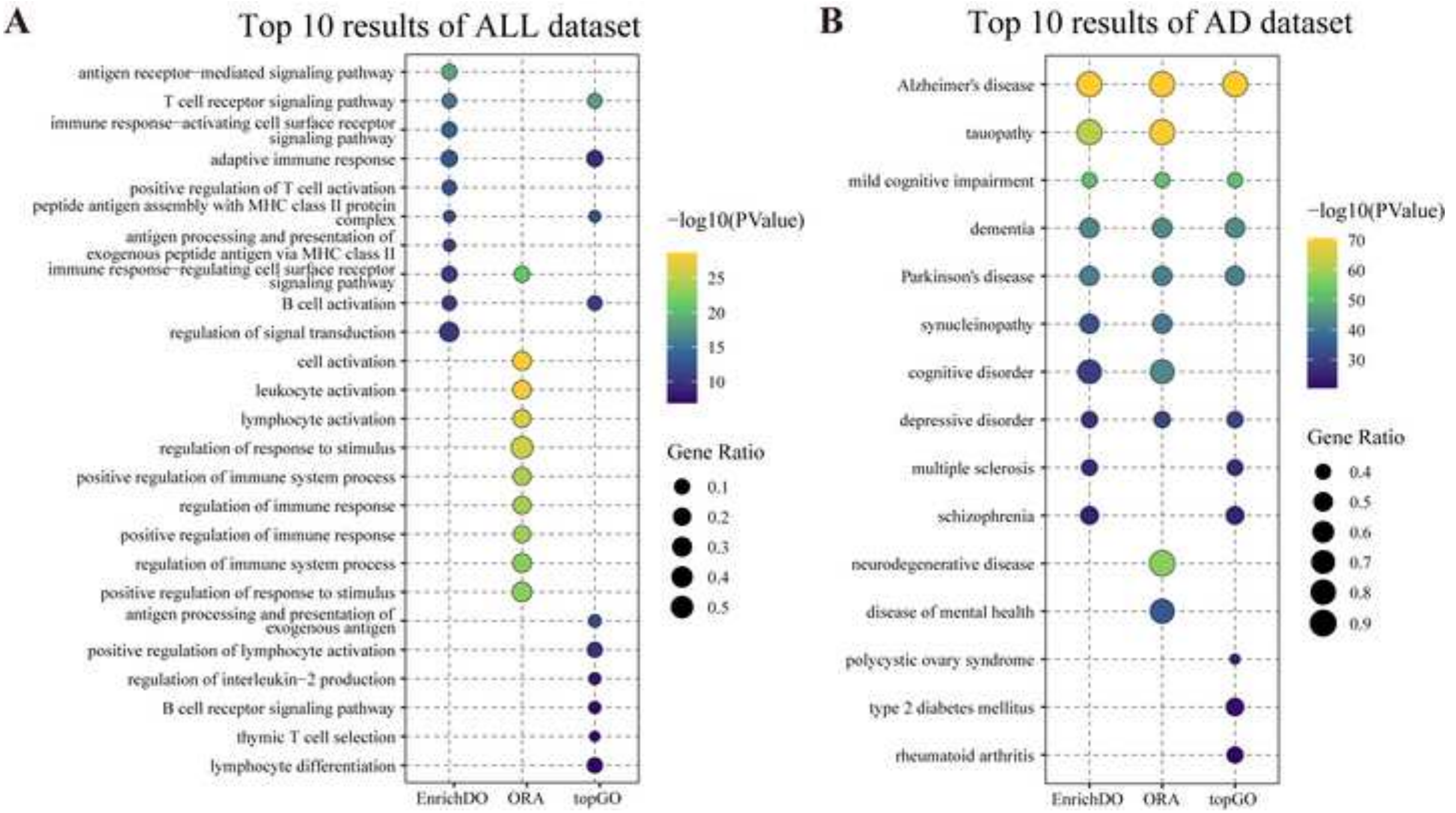

Figure 4

Click here to access/download;Figure;Fig4.tif

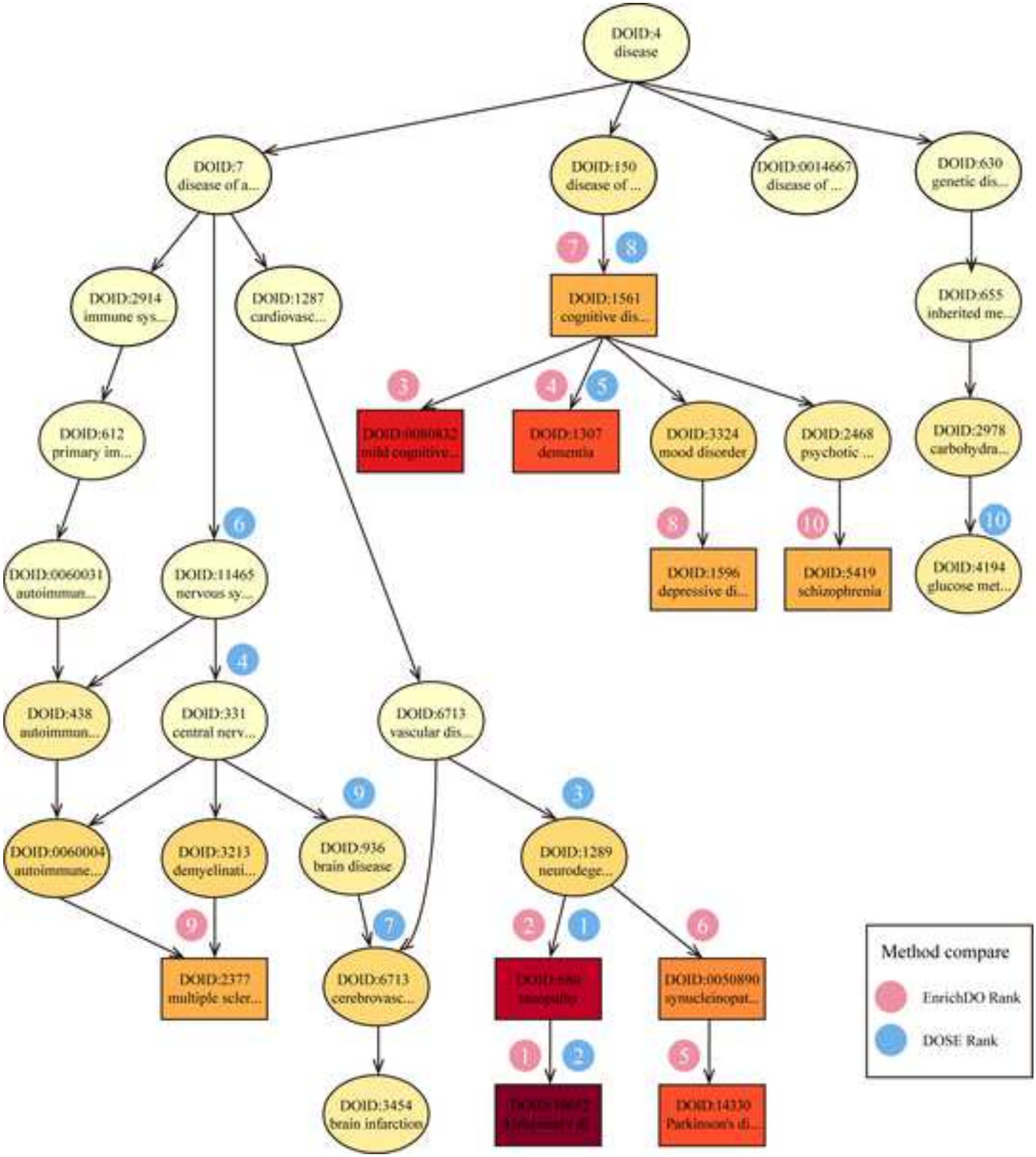

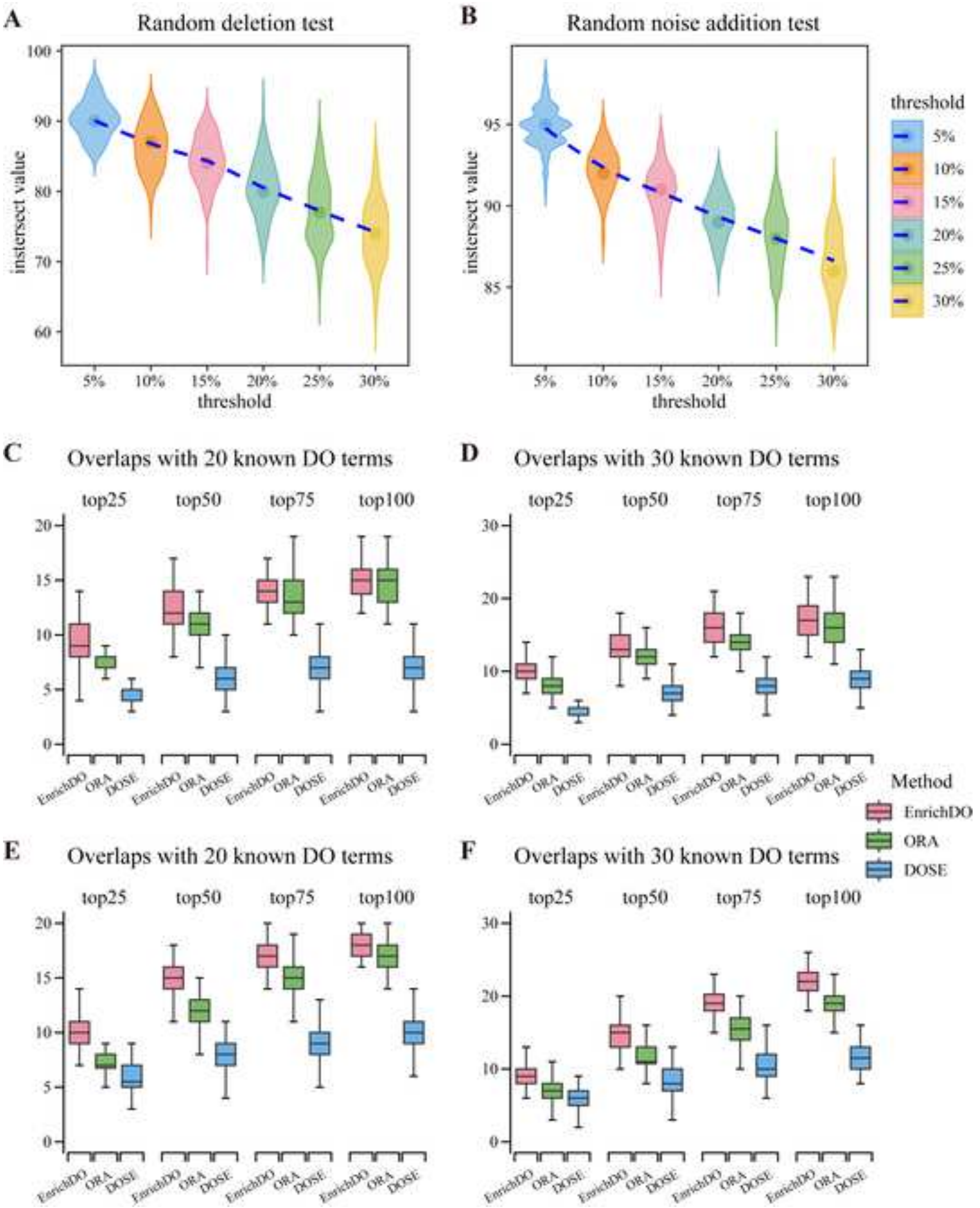
A
Random deletion test
instersect value
threshold
5%
10%
15%
20%
25%
30%
B
Random noise addition test
instersect value
threshold
C
Overlaps with 20 known DO terms
top25
top50
top75
top100
EnrichDO
ORA
DOSE
D
Overlaps with 30 known DO terms
Method
E
Overlaps with 20 known DO terms
F
Overlaps with 30 known DO terms

Figure 6

Click here to access/download;Figure;Fig6.tif

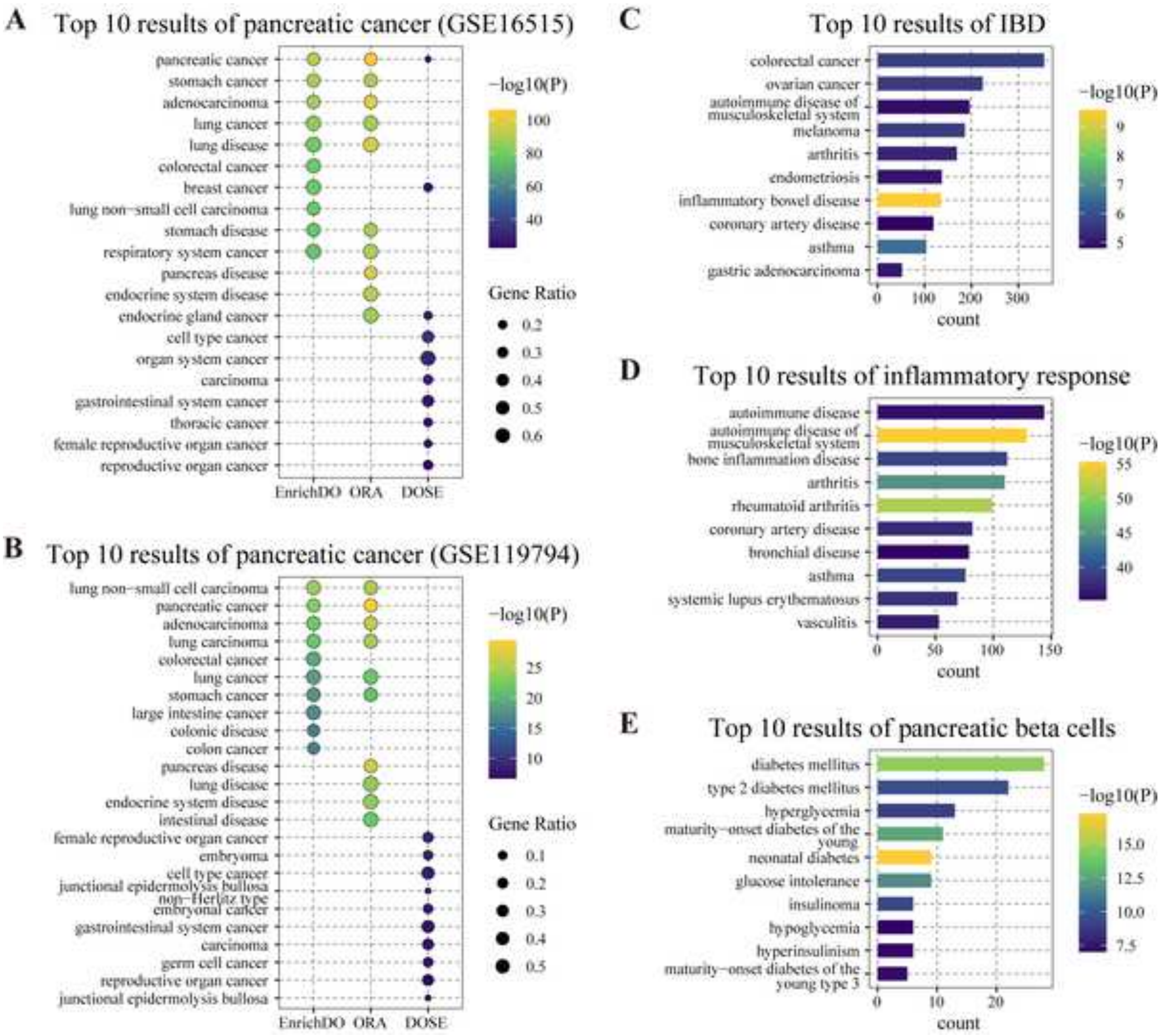

Click here to access/download
Supplementary Material
Figure S1_Supplementary Material.pdf

Click here to access/download
Supplementary Material
Table S1_Supplementary Material.xlsx

Graphical Abstract

Click here to access/download;Graphical Abstract;Graphical Abstract.tif

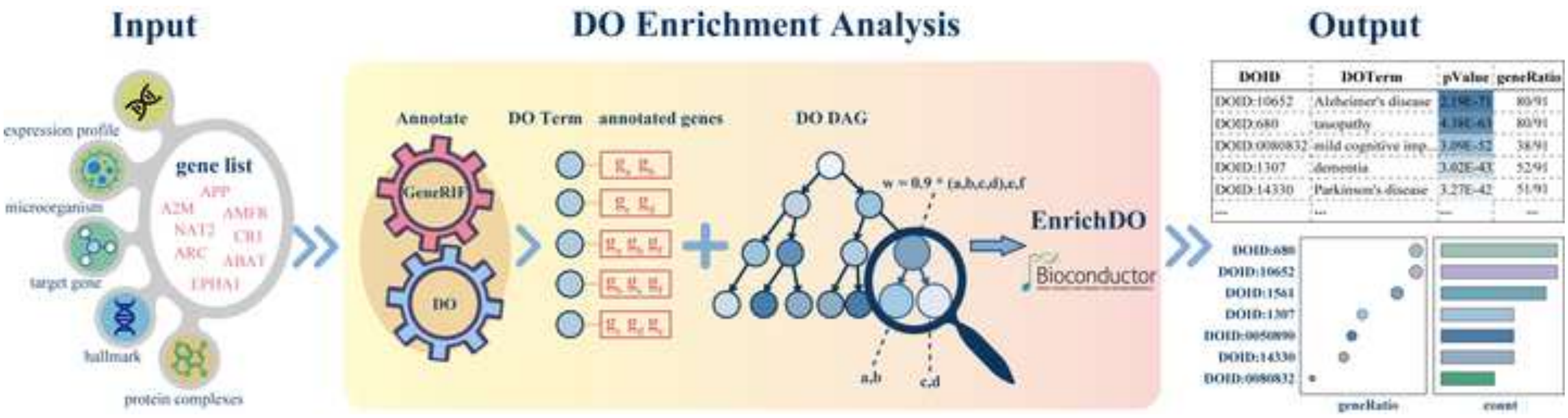

Supplement: giaf021_GIGA-D-24-00357_Original_Submission [file giaf021_giga-d-24-00357_original_submission.pdf]
