# Supplementary material for: EnrichDO: a global weighted model for Disease Ontology enrichment analysis
Source: Gigascience. 2025 Mar 26;14:giaf021. doi: 10.1093/gigascience/giaf021 (PMC11945307; doi:10.1093/gigascience/giaf021)

GigaScience

EnrichDO: a Global Weighted Model for Disease Ontology Enrichment Analysis

--Manuscript Draft--

**Manuscript Number:**	GIGA-D-24-00357R1
**Full Title:**	EnrichDO: a Global Weighted Model for Disease Ontology Enrichment Analysis
**Article Type:**	Technical Note
**Funding Information:**	Tou-Yan Innovation Team Program of the Heilongjiang Province (2019-15) — Dr. Liang Cheng<br>National Natural Science Foundation of China (62222104, 62172130) — Dr. Liang Cheng<br>National Natural Science Foundation of China (61902095) — Dr. Haixiu Yang<br>Heilongjiang Postdoctoral Science Foundation (LBH-Q20030) — Dr. Liang Cheng
**Abstract:**	Background: Disease Ontology (DO) has been widely studied in biomedical research and clinical practice to describe the roles of genes. DO enrichment analysis is an effective means to discover associations between genes and diseases. Compared to hundreds of Gene Ontology (GO)-based enrichment analysis methods, however, DO-based methods are relatively scarce, and most current DO-based approaches are term-for-term and thus are unable to solve over-enrichment problems caused by the “true-path” rule.<br>Results: Here, we describe a novel double-weighted model, EnrichDO, which leverages the latest annotations of the human genome with DO terms and integrates DO graph topology on a global scale. Compared to classic enrichment methods (mainly for GO) and existing DO-based enrichment tools, EnrichDO performs better in both GO and DO enrichment analysis cases. It can accurately identify more specific terms, without ignoring the truly associated parent terms, as shown in Alzheimer's disease (AD) case (AD ranked 1st). Moreover, both simulated test and a data perturbation test validate the accuracy and robustness of EnrichDO. Finally, EnrichDO is applied to other types of datasets to expand its application, including gene expression profile datasets, host gene set of microorganisms, and hallmark gene sets. Based on the findings reported here, EnrichDO shows significant improvement via all experimental results.<br>Conclusions: EnrichDO provides an effective DO enrichment analysis model for gaining insight into the significance of a particular gene set in the context of disease. To increase the usability of EnrichDO, we have developed an R-based software package, which is freely available through Bioconductor (https://bioconductor.org/packages/release/bioc/html/EnrichDO.html) or at https://github.com/liangcheng-hrbmu/EnrichDO.<br>Keywords: Disease Ontology; human genome; enrichment analysis; double weighting
**Corresponding Author:**	Liang Cheng<br>Harbin Medical University<br>CHINA
**Corresponding Author Secondary Information:**	
**Corresponding Author's Institution:**	Harbin Medical University
**Corresponding Author's Secondary Institution:**	
**First Author:**	Haixiu Yang
**First Author Secondary Information:**	
**Order of Authors:**	Haixiu Yang

Powered by Editorial Manager® and ProduXion Manager® from Aries Systems Corporation

	Hongyu Fu
	Meiyi Zhang
	Yangyang Liu
	Yongqun Oliver He
	Chao Wang
	Liang Cheng
**Order of Authors Secondary Information:**	
**Response to Reviewers:**	Dear Prof. Nicole Nogoy and reviewers,<br>Thanks very much for your editorial efforts regarding our manuscript entitled "EnrichDO: a Global Weighted Model for Disease Ontology Enrichment Analysis" (Manuscript ID: GIGA-D-24-00357). We also thank the reviewers for their constructive comments to strengthen this manuscript. Those comments are all valuable and very helpful for revising and improving our paper, as well as the important guiding significance to our research. We have studied comments carefully and have made corrections which we hope meet with approval. Revised portion are marked in RED in the paper. The main corrections in the paper and the responses to the reviewer's comments are as following:<br><br>Reply to Editor<br>In addition, please register any new software application in the bio.tools and SciCrunch.org databases to receive RRID (Research Resource Identification Initiative ID) and biotoolsID identifiers, and include these in your manuscript. Computational workflows should be registered in workflowhub.eu and the DOIs cited in the relevant places in the manuscript. These will facilitate tracking, reproducibility and re-use of your tool.<br>Please include a point-by-point within the 'Response to Reviewers' box in the submission system. Please ensure you describe additional experiments that were carried out and include a detailed rebuttal of any criticisms or requested revisions that you disagreed with. Please also ensure that your revised manuscript conforms to the journal style, which can be found in the Instructions for Authors on the journal homepage. If the data and code has been modified in the revision process, please be sure to update the public versions of this too.<br><br>Response：Thank you very much for your editorial efforts regarding our manuscript. In response to your request, we have registered our software on the bio.tools and SciCrunch.org databases and added RRID and biotoolsID identifiers to the section "Availability of Source Code and Requirements" in main manuscript. We have also registered the computational workflows in workflowhub.eu and added the DOIs to the section "Availability of Source Code and Requirements" in main manuscript.<br>We have provided detailed responses to all the reviewers' comments. We confirmed that the modifications and additional experimental results have been included in the main manuscript and supplementary data as required. In addition, we have adjusted the overall style of the manuscript to ensure compliance with the journal's "Author Guidelines" section. We believe that the manuscript has been greatly strengthened by the critique of the reviewers and hope that both you and the reviewers will now find the paper suitable for publication.<br><br><br><br>Reply to Reviewer #1<br><br>Comments to the Author:<br>In the manuscript entitled "EnrichDO: a Global Weighted Model for Disease Ontology Enrichment Analysis", authors described a new method and a tool for DO enrichment analysis by considering the topology of DO. However, my comment is the manuscript is not clearly written, the methods are not clearly explained and the analysis is not convincing.<br><br>Response：Many thanks to the reviewer for carefully reviewing our work. Those constructive comments are all valuable and very helpful for revising and improving our

Powered by Editorial Manager® and ProduXion Manager® from Aries Systems Corporation

paper. We have studied comments carefully and have made corrections which we hope meet with approval. In the revised manuscript, major revisions are shown in RED color.

Point 1: ## The language
I am very flexible to the English writing when I review manuscripts because I am not a native English speaker either. But in this manuscript, authors should spend more time to improve their English. There are many mistakes and unnatural uses of words and grammar. For example, in the Abstract, "DO-based methods were relatively scare,", scare? I guess it should be "rare"?

Response 1: Many thanks to the reviewer for pointing this out. We apologize for using this word incorrectly. To improve the clarity and readability, the manuscript has been professionally edited by the Open-Science publishing company, GigaScience Press, to correct academic and grammatical errors. All authors have also carefully revised the manuscript version to improve its overall clarity and readability.

Page 1, section “Abstract” in the main manuscript.
“Compared to hundreds of Gene Ontology (GO)-based enrichment analysis methods, however, DO-based methods are relatively scarce, and most current DO-based approaches are term-for-term and thus are unable to solve over-enrichment problems caused by the “true-path” rule.”

Point 2: ## Annotating the Human Genome with DO Terms
Authors link DO terms to genes by using an ontology annotator from gene-related textual descriptions. Have authors validated the quality of such mapping? In the manuscript, authors claimed that their annotation is better because there are more genes annotated to DO terms. There might be false positives in the mapping. The validation can be easily done by manually checking e.g. 1000 mappings, which, according to my experience, only takes 1-2 hours' work.

Response 2: Thank you for your invaluable comment. We accepted reviewer’s advice and validated the quality of the annotations. We randomly extracted 5,000 mappings, except for the root node “disease (DOID=4)” because of its’ lack of specific significance, and manually checked these mappings. Out of 5,000 mappings, there are 4,796 confirmed annotation relationships and 204 incorrect annotations.
In this study, the annotations were obtained by annotating the textual descriptions of gene function (GeneRIF) with DO terms utilizing Mgrep. Mgrep is an efficient tool designed for mapping free text to ontology terms, which was used by NCBO Annotator service. In this study, the false positive is 4.08%, which is within an acceptable range. We have added this manual validation content to the section “Summary of Annotations of the Human Genome with DO” in main manuscript.

Page 12, section “Summary of Annotations of the Human Genome with DO” in the main manuscript.
“High annotation quality is essential to the performance of enrichment analysis. We manually validated the annotations and compared them with the disease and gene annotations of the DGA database [25]. We randomly extracted and manually checked 5,000 annotation mappings, excluding the term “disease” (DOID=4). Out of 5,000 mappings, 204 were incorrectly annotated and the false positive (4.08%) was within an acceptable range, which confirmed the accuracy of the annotations of EnrichDO.”

Point 3: ## Weighted DO Enrichment Analysis
(1) Shouldn't step iv be the the second step because it is actually related to how to calculate score(c) and score(t)?
(2) I don't understand equation 3, and its functions in the adjustment process in this method.
(3) How does it deal with nodes with multiple parents?

Response 3: We apologize for the unclear method description about the section “Weighted DO Enrichment Analysis”. We have revised this section in main manuscript to make the algorithm’s process clearer.
(1) We first define four key concepts: (i) initial weight wi; (ii) dynamic weight wd; (iii) penalty score; (iv) significance score. Then we described the algorithm process

through five steps. In step 2, we first calculated the significance score for the current node t using concept mentioned in (iv), then calculated the dynamic weights wd for t and each child of t (mentioned in (ii)), and divided the children into two groups according to wd.

(2) Equation 3 is a penalty value for gene weights other than wd. We defined the penalty score based on a large number of experimental results, and used it to alleviate the impact of different magnitudes of p-values. As described in concepts (ii), the dynamic weights was defined as w_d=(log(score(c)))/(log(score(t))) , which score(.) is the p-value of weighted hypergeometric test. Given two groups, (1) score(c1)=1e-1, score(t1)=1e-2; (2) score(c2)=1e-25, score(t2)=1e-50; the wd was both 0.5, thus the weight of genes of child nodes for two groups is the same. However, the magnitudes of the two groups' p-value are significantly different, and it is inappropriate to down-weight the child nodes for two groups equally. Thus we further down-weighted the child nodes according to the significance of the parent and child nodes as shown in equation 3.

(3) Node with multiple parents can be operated normally according to the algorithm process, as we mainly focus on comparing the current node with its children. For the current node t, when the child node c is more significant than t, the genes of c in node t and all its ancestors (including multiple parents of t) should be down-weighted. While the other parent nodes of c do not participate in the current calculation.

We have revised the section "Weighted DO Enrichment Analysis" in main manuscript.

Page 9-11, section "Weighted DO Enrichment Analysis" in the main manuscript.

"We first define four key concepts:

(i) Initial weight wi. Initially, for each DO term, the weights for all directly annotated genes are set to 1, and the weights for indirectly annotated genes decrease by 0.1 for each level inherited upward. The smaller the weights of indirectly annotated genes, the less the contribution that these genes have to the enrichment analysis. Notably, smaller weights can cause certain nodes to be missed during the enrichment analysis. In this study, the weights for indirectly annotated genes were no less than 0.5.

(ii) Dynamic weight wd. For a given DO term t and its child c, the weight assigned to genes annotated to this pair of terms is defined as follows:

w_d=(log(score(c)))/(log(score(t)))   (2)

where score(.) is the p-value of the hypergeometric test with weighted genes. The weights for genes annotated to each node are memorized and updated during the process.

(iii) Penalty score. The penalty score is a penalty value for gene weights other than wd, that is used to alleviate the impact of different magnitudes of p-values. It is defined as follows:

penal=max(1/10×lg(xmin)/(lg(score(t))+lg(score(c))),1)   (3)

where xmin is the minimum positive number in R and score(.) is the p-value of the hypergeometric test with weighted genes mentioned above.

(iv) Significance score. The significance score for a given DO term t is calculated by applying the hypergeometric test with weighted genes. The number r in formula (1), which indicates the number of interesting genes included in term t, is replaced by rounding down the sum of the weight of r genes. The number m, which indicates the number of genes annotated to term t, is replaced by rounding down the sum of the weight of m genes.

EnrichDO is a double-weighted iterative model. Given a threshold of 0.01 (or less), our aim is to find significantly enriched DO terms with p-values < 0.01. The overall process of the algorithm is shown in Figure 1A, and the detailed process is described step-by-step as follows:

Step 1. Given the DAG with levels (mentioned in "Semantic Expansion in DO DAG"), we set initial weights for annotated genes for each node. We process the nodes bottom-up, from the highest level and then iteratively move to nodes at lower levels, with the aim of identifying the most specific nodes with minimum required significance (as shown in Figures 1A and 1B). For the current node t, all its children have significance scores; children with p-values larger than the threshold are excluded, as shown in Figures 1A and 1B. The node t is then processed by step 2 to step 5, as shown in Figure 1C.

Step 2. For the current node t, we calculate its significance score, using concept (iv) described above. If the score is larger than the threshold, then the process moves to the next node. Otherwise, t is compared to each of its children to discover the most significant nodes locally. In detail, for each child c, we calculate the weight wd for the pair of terms. When wd >1, it indicates that c is more significant than t, thus node c is a

Powered by Editorial Manager® and ProduXion Manager® from Aries Systems Corporation

local optimum, and c is moved from children to sigChildren; otherwise, c remains in children. This step was shown in Figure 1C-(1).
Step 3. For each child c in sigChildren, the weight of the genes annotated to c is decreased, by dividing term t and all its ancestors by wd and the penalty score. The child c is then removed from sigChildren, until the sigChildren is empty. This step is shown in Figure 1C-(2).
Step 4. Steps 2-3 are executed recursively. The significant score of t is recalculated with updated weights and is compared to its remaining children. This process is carried out until either of the two following situations arises: (1) there are no remaining children, in which case the process moves to the next node, or (2) remaining children exist, in which case the process moves to step 5.
Step 5. For each remaining child c, it is less significant than t. Thus, node t is a local optimum, and the weight of genes annotated to c is decreased by multiplying wd (wd <1) and dividing it by the penalty value, thereby, reducing the contribution of genes to the child nodes. The significance score is then recalculated, and the process moves to the next node. This step is shown in Figure 1C-(3)."

Point 4: ## Comparison with Other Methods
(1) topGO by default also uses ORA, so it is actually the same as ORA in the comparison. Authors can see it in Table 3 where ORA and topGO give the same p-values.
I noticed DO terms 2, 6, 11 and 12 have p-value of 1 by topGO. What is the reason for that? Is it because topGO has a different cutoff for minimal number of genes in a gene set?
(2) Only compare top 10 terms is not sufficient to conclude which tool is better than others. As authors can see in the tables, these top significant gene sets are with very small p-values. Under this context, the ranking of gene sets is less important because they are "all significant and important".
(3) And I didn't see big difference between EnrichDO and ORA.

Response 4: Many thanks to the reviewer for pointing this out. (1) topGO applied the Fisher's exact test on a weighted contingency table. We thoroughly studied the algorithm and code of topGO. For the first scenario, DO terms 1, 3-5, 7-10, 13-15 obtained the same p-values as ORA, because they were leaf nodes or more significant than their child nodes, thus the p-values of these nodes remained the initial ORA p-values that calculated at the beginning. For the second scenario, DO terms 2, 6, 11, and 12 obtained p-value of 1 by topGO, because they had child nodes more significant than themselves, thus the topGO down weighted the current node by multiplying it with wd (wd=0.5) and the penalize score (1/(|(log(p(t)*p(c)))/2|+1)), so the genes were significantly down-weighted, resulting in p-value of 1.
(2) We accepted reviewer's advice and conducted other comparisons of the significant results of three methods. The interesting gene list of Alzheimer's disease (AD) case was collected from the DisGeNET database, which was manually curated AD-related data, resulting in low p-values of three methods. We set strict thresholds with p-value < 0.01, p-adjust < 0.01, the hypergeometric test identified 859 significant terms, far exceeding those identified by EnrichDO (405) and topGO (272). We investigated top 50, 100, and 200 significant results of the three methods in the DAG. As illustrated in Supplementary Table 2, ORA's results are more general, located at lower levels, and primarily converged on same branches. Conversely, the results of topGO are more specific, located at higher levels, and distributed across distinct branches.
The above results indicated that ORA performed enrichment term-for-term, cannot solve over-enrichment problem caused by the "true-path" rule. In contrast, topGO recognized more specific terms, but at the same time, it might ignore some truly relevant nodes, such as tauopathy, and synucleinopathy in Table 3 in main manuscript. In comparison, EnrichDO represented a moderate approach that identified more specific terms as well as not neglected the true significant terms, making it more suitable for DO enrichment analysis.
Finally, when there are quite a few significant results of the statistic model, we think that not all results within the threshold are as important. For example, autosomal genetic disease (DOID:0050739) has a significant p-value and ranks 225th, 76th, and 245th in the results of EnrichDO, ORA, and topGO, respectively. However, to our knowledge, it is not associated with AD. Therefore, we believe that the ranking of terms in the results is also important, that many studies used top n terms for graphical display and even for further experimental verification.

Powered by Editorial Manager® and ProduXion Manager® from Aries Systems Corporation

(3) As stated in (2), the ORA cannot solve over-enrichment problem, and the results were more general, that usually be the ancestors of EnrichDO's results. Therefore, EnrichDO is more accurate that can identify more specific diseases.
We have revised the section “Comparison with Other Methods” in main manuscript.

Page 15-16, section “Comparison with Other Methods” in the main manuscript.
“The phenomenon is more evident in other comparisons of the significant results. Setting the threshold p-value < 0.01, and p-adjust < 0.01, the hypergeometric test identified 859 significant terms, far exceeding those identified by EnrichDO (405) and topGO (272). We investigated top 50, 100, and 200 results of the three methods in the DAG. The results of the hypergeometric test are more general, located at lower levels, and primarily converged on same branches. Conversely, the results of topGO are more specific, located at higher levels, and distributed across distinct branches (Supplementary Table 2). The above results indicate that the hypergeometric test performed enrichment term-for-term, cannot solve over-enrichment problem, while topGO recognized more specific terms, but might overlook truly relevant nodes. In contrast, EnrichDO often yields more accurate, more specific, and more significant DO terms, thereby overcoming the over-enrichment problem. At the same time, it is also a moderate weighted algorithm that will not neglect true significant terms, even though their child nodes are significant.”

Point 5: ## Comparison with Current DO-based Enrichment Analysis Tools
DOSE also uses ORA, but with a different default of background. By default, DOSE uses all DO genes as the background while not all protein-coding genes. If authors explicitly set all protein-coding genes to the "universe" argument in 'EnrichDO()", they might have very similar results as enrichDO.

Response 5: Many thanks to the reviewer for pointing this out. We accepted reviewer's advice and conducted a comparison in consistent background. Due to the background of EnrichDO was constructed by annotating all protein-coding genes (pc-genes) with DO terms, we extracted pc genes from the background data of DOSE and implemented the enrichment analysis. The new enrichment results of DOSE did not show a trend of "lower p-values", and the order of significant terms did not change obviously, as show in Supplementary Table 3.
We have revised the section “Comparison with Current DO-based Enrichment Analysis Tools” in the main manuscript.

Page 17, section “Comparison with Current DO-based Enrichment Analysis Tools” in the main manuscript.
“Another explanation for the results is that the annotation data of DOSE has not been updated and therefore contains only 10,312 annotated genes, compared to 15,106 pc genes in EnrichDO. Furthermore, DOSE obtains similar results when the enrichment background is restricted to pc genes (Supplementary Table 3).”

Point 6: ## Stability Assessment
This is not strange. The counts in ORA are always huge, which results in tiny p-values. Simply speaking, if p is the fraction of differential genes from total genes (bgRatio in https://github.com/liangcheng-hrbmu/EnrichDO/blob/devel/inst/examples/result.txt), and N is the number of genes in the gene set, as long as the observed counts is larger than pN, even with a small difference, there will be a significant p-value.

Response 6: Many thanks to the reviewer for pointing this out. We apologize for this inaccurate description. We intended to test the robustness of EnrichDO by performing data removal and noise addition test. For AD case, we randomly deleted data (or added noise) at 5% intervals, and repeated enrichment 100 times for each interval. When the disturbance of inputs was up to 30%, the number of input interesting gene set has decreased from 91 to 64, and the ratio of overlapped DO terms to original significant DO terms still remained 74.17%, which indicated that the EnrichDO is robust to data removal or noise addition. We have corrected the section “Stability Assessment” to “Robustness of EnrichDO” in main manuscript, and revised this section in main manuscript.

Page 17-18, section “Robustness of EnrichDO” in the main manuscript.
“We tested the robustness of EnrichDO with the AD dataset by removing interesting

Powered by Editorial Manager® and ProduXion Manager® from Aries Systems Corporation

genes and by adding noise, respectively. First, we removed the AD dataset at 5% intervals and repeated the EnrichDO method 100 times for each removal. The number of significant overlapped DO terms (top 100) decreased slowly compared to the original data, and the ratio of overlapped DO terms to original significant DO terms remained at 74.17%, even after removal of up to 30% of the AD dataset (Figure 5A). These results indicate that the EnrichDO method is robust to data removal. In a similar way, noise was added to the AD dataset. We added 5% to 30% noise from a background gene set, at 5% intervals, repeated 100 times. Significantly overlapped DO terms in the top 100 decreased from 94.8 to 86.6 (Figure 5B). The results highlight the robustness of EnrichDO to data noise."

Reply to Reviewer #2:

Comments to the Author:
The manuscript by Yang et al, titled "EnrichDO: a Global Weighted Model for Disease Ontology Enrichment Analysis" presents EnrichDO, a new R package for weighted functional enrichment analysis of gene datasets with Disease Ontology (DO) terms. Enrichment analysis is a staple in current methods used in Systems Biology for the large-scale annotation of gene datasets; therefore, although a large number of tools are already available in the field, a new implementation is always welcome, provided it can offers users novel features not presented by other implementations. The authors attempt to do just that, by presenting a tool that provides weighted enrichment results from DO, using data provided by annotating the human genome, and applying a directed acyclic graph (DAG) to take DO term inheritance into account and reduce term redundancy and biased over-representation. Such a methodology has been applied to Gene Ontology (GO enrichment) a few times before, but rarely to disease annotations; therefore, EnrichDO can become a useful tool for the community by providing this functionality.
Despite its importance, the manuscript, and the EnrichDO R package itself, need to undergo some REVISIONS, before they can be accepted for publication:

Response：Many thanks for acknowledging our efforts. We have studied comments carefully and have revised both manuscript and the R package which we hope meet with approval.

Manuscript points:

Point 1: Although the manuscript is, for the most part, well-written (clear description of the methods, distinct sections, case studies etc), the language of the text in many places is poor. The manuscript needs to be extensively proofed by a competent English speaker.

Response 1: Many thanks to the reviewer for pointing this out. To improve the clarity and readability, the manuscript has been professionally edited by the Open-Science publishing company, GigaScience Press, to correct academic and grammatical errors. All authors have also carefully revised the manuscript version to improve its overall clarity and readability. In the revised manuscript, major revisions are shown in RED color.

Point 2: Since the authors have constructed their own reference background for enrichment, by mapping the human genome with DO terms, they should add a discussion segment detailing their plans for maintaining and updating this reference dataset. Enrichment analysis tools are only as good as their background data; since new information is continuously being released, this data should always be kept up to date.

Response 2: Thank you for your invaluable comment. We accept reviewer's advice and will continuously update the background data of EnrichDO annually. Since the gene description file (generifs_basic.gz) from the GeneRIF database and the Disease ontology file (doid.obo) from the disease ontology database are both open source, it is feasible to maintain and updating the annotations with our pipeline of data processing. We have added the data update vision in the section "Discussion" and revised the

Powered by Editorial Manager® and ProduXion Manager® from Aries Systems Corporation

section “Availability of Source Code and Requirements” in main manuscript.

Page 24, section “Discussion” in the main manuscript.
“To facilitate the use of our model, we have developed an R-based software package, which is freely available through Bioconductor (https://bioconductor.org/packages/release/bioc/html/EnrichDO.html) or at https://github.com/liangcheng-hrbmu/EnrichDO. Background annotations will be updated annually according to the latest information in the GeneRIF and DO databases, and the EnrichDO package will be continuously maintained.”
Page 25, section “Availability of Source Code and Requirements” in the main manuscript.
“Project home page: https://bioconductor.org/packages/release/bioc/html/EnrichDO.html, the latest version can be available at https://www.bioconductor.org/packages/devel/bioc/html/EnrichDO.html or https://github.com/liangcheng-hrbmu/EnrichDO.”

Point 3: Right now, the tool exists solely as an R package. End users, particularly wet lab scientists with little to medium bioinformatics/R knowledge (who are usually the majority of people using these enrichment tools), may find this daunting. Are there any plans to provide either a user interface (in a form of e.g. a Shiny app) or a web server version of the method? This would greatly increase the visibility and accessibility of the tool. This is in no way a request to implement such an interface for this current paper; just a recommendation for a future update.

Response 3: Thank you for your suggestion. It is also the goal of EnrichDO to serve more scientists without any biological or programming skills. To this end, we have developed a user-friendly web server, named “OntoTiger”, to enabling one-stop ontology-based applications, which can be freely available at http://bio-annotation.cn/OntoTiger. EnrichDO will be integrated as the underlying algorithm in enrichment analysis module once it was published. We will synchronous update this web service information of EnrichDO in github when the OntoTiger is released.

Tool points:

Point 1: Although the manuscript states that EnrichDO is compatible with R v. 3.5 or higher, I was unable to install it in an R 3.6.1 installation; primarily due to some of the tools' dependencies no longer being available/maintained for such old R versions. Standard Bioconductor installation was also problematic in R version 4.1; eventually, I was able to install the development version of the package through GitHub. The authors should make sure that everything can be installed as intended, and to make sure that the reported minimum requirements for R are correct.

Response 1: Many thanks to the reviewer for pointing this out. We apologize for the incorrect description of the version information. The EnrichDO package was released on Bioconductor 3.20，which is designed for R 4.4 version. Thus standard Bioconductor installation can be executed on R 4.4 and above versions. For version below R 4.4 (minimum R4.0), users can download EnrichDO and its dependency packages and install package(s) from local files. We have corrected the minimum requirements for R in section “Availability of Source Code and Requirements” in main manuscript.

Page 25, section “Availability of Source Code and Requirements” in the main manuscript.
“Programming language: It is recommended to use R version 4.4 and Bioconductor version 3.20 or later. For versions below R-4.4 (minimum R-4.0), users can download source packages from Bioconductor and install package(s) from local files.”

Point 2: I tried testing the tool by following the example in the tool's vignette. The vignette example is very barebone; more detailed information should be given. The same goes for the manual, it should be more documented. Since this is an R package, a lot of people are going to want to implement EnrichDO as part of their greater analysis workflow; having a good documentation would be a great help to that.

Powered by Editorial Manager® and ProduXion Manager® from Aries Systems Corporation

Response 2: Thank you for your invaluable comment. We accepted reviewer's advice and rewritten the EnrichDO's vignette and manual. Users can easily implement DO enrichment analysis by following the instructions of vignette or manual, which can be obtained by the command "browseVignettes(EnrichDO)" or "??EnrichDO". We hope that our revised vignette and manual of the R package will meet with approval. The latest version can be available at
https://www.bioconductor.org/packages/devel/bioc/html/EnrichDO.html or
https://github.com/liangcheng-hrbmu/EnrichDO.

Point 3: The tool seems to perform very poorly in terms of speed and responsiveness. This may have to do with the fact that I used the development version instead of the Bioconductor one; however, even in that case, the performance is too poor for the specifications of the computer used for testing (8 CPU cores, 32 GB RAM). The authors should try to optimize their implementation as much as possible to improve its performance.

Response 3: Many thanks to the reviewer for pointing this out. The case studies in EnrichDO have met the performance requirements of Bioconductor, with run times under 10 seconds on Windows, under 10 seconds on macOS, and under 5 seconds on Linux System.
Additionally, EnrichDO is an iterative model that process the nodes layer by layer, resulting in slower running speed compared to the term-for-term approach (ORA methods). We have tested the AD case on different computers (minimum 4 CPU cores, 8 GB RAM), and the run times were all less than 10 seconds. It worth noting that if the input gene set intersects with many DOTerms, the running time of EnrichDO will slow down. Additionally, to prevent users from waiting for too long, EnrichDO provides feedback on the calculation progress at each layer in the console interface, allowing users to roughly be aware of the progress of the EnrichDO calculations.

Reply to Reviewer #3:

Comments to the Author:
The manuscript offers a novel method for Disease Ontology (DO) enrichment. Although the field already contains numerous tools, new tools that bring unique features are always appreciated.
Although the manuscript is generally well-organized (with clear descriptions of methods, case studies, and defined sections), many passages suffer from language issues. It would benefit from thorough proofreading by a proficient English editor.

Response：Many thanks to the reviewer for pointing this out. To improve the clarity and readability, the manuscript has been professionally edited by the Open-Science publishing company, GigaScience Press, to correct academic and grammatical errors. All authors have also carefully revised the manuscript version to improve its overall clarity and readability. In the revised manuscript, major revisions are shown in RED color.

Point 1: The authors generated a unique reference background for enrichment by mapping DO terms to the human genome, yet there is no mention of how they intend to keep this dataset current. For example, DisGeNet has already become private and is no longer accessible. Same for OMIM. What about integrating other disease-related databases (e.g., MedGen)?

Response 1: Thank you for your invaluable comment. We have carefully studied the MedGen database. It serves as a portal for information on genetic aspects of human health and disease. MedGen integrate information from various sources, but not focus on Disease Ontology, thus it is not suitable for annotations for DO enrichment.
In our work, we annotated the human genome (GeneRIF) with disease ontology (Disease Ontology) terms to generate reference background for enrichment. Since the GeneRIF and Disease Ontology database are both open source, it is feasible update the annotations with our pipeline of data processing, and we will continuously update the background data of EnrichDO annually. We have added the data update vision in the section "Discussion" and revised the section "Availability of Source Code and Requirements" in main manuscript.

Page 24, section "Discussion" in the main manuscript.
"To facilitate the use of our model, we have developed an R-based software package, which is freely available through Bioconductor (https://bioconductor.org/packages/release/bioc/html/EnrichDO.html) or at https://github.com/liangcheng-hrbmu/EnrichDO. Background annotations will be updated annually according to the latest information in the GeneRIF and DO databases, and the EnrichDO package will be continuously maintained."
Page 25, section "Availability of Source Code and Requirements" in the main manuscript.
"Project home page: https://bioconductor.org/packages/release/bioc/html/EnrichDO.html, the latest version can be available at https://www.bioconductor.org/packages/devel/bioc/html/EnrichDO.html or https://github.com/liangcheng-hrbmu/EnrichDO. "

Point 2: Possibility to access EnrichDO through the web? A web application would be useful.

Response 2: Thank you for your suggestion. It is also the goal of EnrichDO to serve more scientists without any biological or programming skills. To this end, we have developed a user-friendly web server, named "OntoTiger", which dedicated to enabling one-stop ontology-based applications. EnrichDO will be integrated as the underlying algorithm in enrichment analysis module once it was published. We will synchronous update this web service information of EnrichDO in github when the OntoTiger is released. Currently, the OntoTiger can be freely available at http://bio-annotation.cn/OntoTiger.

Point 3: Many references related to functional enrichments are missing - Examples are: EnrichR, WebGestaldt, Flame, aGOtool etc.

Response 3: Many thanks to the reviewer for pointing this out. We overlooked these enrichment tools, they are all excellent enrichment web services，especially Flame (v2.0), which also supports DO enrichment analysis. We have carefully studied these tools and have added these references to the article. In addition, we have implemented enrichment analysis with Flame (v2.0) and conducted a comparison with our method. We have revised the section "Background" and added the comparison results in the section "Comparison with Current DO-based Enrichment Analysis Tools" in the main manuscript.

Page 4, section "Background" in the main manuscript.
"Similar web service tools include EnrichR [27], WebGestaldt [28], Flame (V2.0) [29], and aGOtool [30], all of which provide disease enrichment analysis by integrating multiple disease data sources."
Page 17, section "Comparison with Current DO-based Enrichment Analysis Tools" in the main manuscript.
"Flame had three overlaps with EnrichDO: "AD," "dementia," and "cognitive disorder." Flame recognized AD as the top correlated disease; however, in comparison to EnrichDO, other results obtained by Flame were more general, as shown in Figure 4. In addition, the branches of Flame results are more similar to DOSE (with six overlaps), and there is a branch related to metabolic diseases."

Point 4: The tool's performance seemed suboptimal, showing slow processing times and low responsiveness. The authors should consider optimizing the package to enhance its speed and responsiveness.

Response 4: Many thanks to the reviewer for pointing this out. The case studies in EnrichDO have met the performance requirements set by Bioconductor, with run times under 10 seconds on Windows, under 10 seconds on macOS, and under 5 seconds on Linux System.
Additionally, EnrichDO is an iterative model that process the nodes layer by layer, resulting in slower running speed compared to the term-for-term approach (ORA methods). We have tested the AD case on different computers (minimum 4 CPU cores, 8 GB RAM), and the run times were all less than 10 seconds. It worth noting that if the

	input gene set intersects with many DOTerms, the running time of EnrichDO will slow down. Additionally, to prevent users from waiting for too long, EnrichDO provides feedback on the calculation progress at each layer in the console interface, allowing users to roughly be aware of the progress of the EnrichDO calculations.
Additional Information:	
Question	Response
Are you submitting this manuscript to a special series or article collection?	No
**Experimental design and statistics**<br><br>Full details of the experimental design and statistical methods used should be given in the Methods section, as detailed in our Minimum Standards Reporting Checklist. Information essential to interpreting the data presented should be made available in the figure legends.<br><br>Have you included all the information requested in your manuscript?	Yes
**Resources**<br><br>A description of all resources used, including antibodies, cell lines, animals and software tools, with enough information to allow them to be uniquely identified, should be included in the Methods section. Authors are strongly encouraged to cite Research Resource Identifiers (RRIDs) for antibodies, model organisms and tools, where possible.<br><br>Have you included the information requested as detailed in our Minimum Standards Reporting Checklist?	Yes
**Availability of data and materials**<br><br>All datasets and code on which the conclusions of the paper rely must be either included in your submission or deposited in publicly available repositories (where available and ethically appropriate), referencing such data using	Yes

a unique identifier in the references and in the “Availability of Data and Materials” section of your manuscript.<br><br>Have you have met the above requirement as detailed in our Minimum Standards Reporting Checklist?	

Powered by Editorial Manager® and ProduXion Manager® from Aries Systems Corporation

Manuscript

Click here to access/download;Manuscript;Manuscript (GIGA-D-24-00357).pdf

EnrichDO: a Global Weighted Model for Disease Ontology Enrichment Analysis

Haixiu Yang[1,†,*], Hongyu Fu[1,†], Meiyi Zhang[1], Yangyang Liu[1], Yongqun Oliver He[3], Chao Wang[1], and Liang Cheng[1,2,*]

[1] College of Bioinformatics Science and Technology, Harbin Medical University, Harbin, Heilongjiang, 150081, China.

[2] National Health Commission (NHC) Key Laboratory of Molecular Probes and Targeted Diagnosis and Therapy, Harbin Medical University, Harbin, 150028, China.

[3] University of Michigan Medical School, Ann Arbor, MI, USA.

[*] Correspondence address. Liang Cheng, College of Bioinformatics Science and Technology, Harbin Medical University, Harbin, Heilongjiang, 150081, China. Email: liangcheng@hrbmu.edu.cn; Haixiu Yang, College of Bioinformatics Science and Technology, Harbin Medical University, Harbin, Heilongjiang, 150081, China. Email: yanghaixiu@ems.hrbmu.edu.cn

[†] Contributed equally.

ABSTRACT

Background: Disease Ontology (DO) has been widely studied in biomedical research and clinical practice to describe the roles of genes. DO enrichment analysis is an effective means to discover associations between genes and diseases. Compared to hundreds of Gene Ontology (GO)-based enrichment analysis methods, however, DO-based methods are relatively scarce, and most current DO-based approaches are term-for-term and thus are unable to solve over-enrichment problems caused by the “true-path” rule.

Results: Here, we describe a novel double-weighted model, EnrichDO, which leverages the latest annotations of the human genome with DO terms and integrates DO graph topology on a global scale. Compared to classic enrichment methods (mainly for GO) and existing DO-based enrichment tools, EnrichDO performs better in both GO and DO enrichment analysis cases. It can accurately identify more specific terms, without ignoring the truly associated parent terms, as shown in Alzheimer's disease (AD) case (AD ranked 1st). Moreover, both simulated test and a data perturbation test validate the accuracy and robustness of EnrichDO. Finally, EnrichDO is applied to other types of datasets to expand its application, including gene expression profile datasets, host gene set of microorganisms, and hallmark gene sets. Based on the findings reported here, EnrichDO shows significant improvement via all experimental results.

Conclusions: EnrichDO provides an effective DO enrichment analysis model for gaining insight into the significance of a particular gene set in the context of disease. To increase the usability of EnrichDO, we have developed an R-based software package, which is freely available through Bioconductor (https://bioconductor.org/packages/release/bioc/html/EnrichDO.html) or at https://github.com/liangcheng-hrbmu/EnrichDO.

Keywords: Disease Ontology; human genome; enrichment analysis; double weighting

Background

Biomedical ontologies organize biomedical findings into hierarchical structures and controlled vocabularies, which are human-readable and machine-computable. Biomedical ontologies have been widely studied and applied in biomedical research and clinical practice [1]. Gene Ontology (GO) is arguably the most successful example of a biomedical ontology. It provides structured, controlled vocabularies and classifications that cover several domains of molecular and cellular biology and that are freely available for community use in the annotation of genes, gene products and sequences [2, 3]. Disease Ontology (DO) is another representative example. DO

organizes, represents, and standardizes human diseases through extensive cross-mapping and integration of MeSH, ICD, NCI's thesaurus, SNOMED CT, and OMIM disease-specific terms and identifiers [4, 5]. Other widely used biomedical ontologies, such as the Human Phenotype Ontology (HPO) [6, 7], Chemical Entities of Biological Interest (ChEBI) [8, 9], and the Ontology of Adverse Events (OAE) [10], have been developed and are included in the BioPortal [11, 12] and/or the OBO Foundry [13, 14]. With the development of biomedical ontologies, numerous excellent algorithms, tools, and platforms have emerged, including annotation, similarity calculation, enrichment analysis, and function prediction. For example, AmiGO [15] and GO-CAM [16] enable functional annotations of genes and gene products, GOSemSim provides an R package for measuring semantic similarity among GO terms and gene products [17], PANTHER is a web service for GO enrichment analysis [18], and PhenIX effectively diagnoses genetic diseases through computational phenotype analysis of disease-associated genomes [19]. However, most state-of-the-art methods are focused on GO, where DO-based analyses mainly focus on similarity calculation [20-22].

Biomedical ontology-based enrichment analysis can help to elucidate the potential biological significance of a particular set of genes, such as differentially expressed gene lists of high-throughput experiments. Thousands of studies have been conducted by GO-based enrichment, but DO-based enrichment methods remain relatively scarce. Osborne et al. used the Unified Medical Language System (UMLS) MetaMap Transfer tool (MMTx) and the Gene Reference Into Function (GeneRIF) database to annotate the human genome with DO for the first time [23]. LePendu et al. annotated GO annotation files with DO terms using the NCBO annotator, facilitating DO enrichment analysis via a simple binomial model [24]. The disease and gene annotations database (DGA) employs NCBO Annotator and GeneRIF to semantically annotates human genes with disease descriptors [25]. Based on the annotations of the human genome with DO, DO-based enrichment analysis is capable of discovering disease associations in high-throughput biological data. For example, KOBAS-i incorporates five human disease

databases and provides a web server for annotation and identification of enriched diseases by binomial test and false discovery rate (FDR) correction [26]. Similar web service tools include EnrichR [27], WebGestaldt [28], Flame (V2.0) [29], and aGOtool [30], all of which provide disease enrichment analysis by integrating multiple disease data sources. DOSim [20] and DOSE [31] are R packages widely used for enrichment analysis that employ the hypergeometric model and gene set enrichment analysis. The web tool ADEPTUS, which is based on high-quality curated databases with information on gene expression profiles and diseases, enables various functional genomics analyses, including DO enrichment analysis [32].

Obviously, DO-based annotation and enrichment are relatively scarce, and current DO enrichment methods are lacking. On one hand, gene-DO annotations and DO terms of the current methods are outdated, and some web services for DO enrichment are inaccessible, due to lack of maintenance. On the other hand, these methods are based on classic models, such as the hypergeometric test, Fisher's exact test, the χ^2-test, and the binomial test, which do not take dependencies between DO terms into consideration. DO has a hierarchical structure that forms a directed acyclic graph (DAG) that follows the "true-path" rule, which means if a gene is annotated to node *t*, it is also annotated to all parent terms of t. This inheritance problem can lead to some terms being over enriched, which has been well addressed in GO-based enrichment methods [33, 34].

In this study, we annotated the human genome with DO, utilizing the latest data source, and developed a novel DO enrichment method, EnrichDO. EnrichDO tackled the "inheritance problem" moderately, by considering the DO graph topology on a global scale, and double-weighted the annotated genes. We compared EnrichDO with classic enrichment methods and current DO-based enrichment tools and evaluated the performance and robustness of EnrichDO. We also performed EnrichDO on datasets directly related to disease (including a microarray gene-expression dataset and an RNA-seq expression dataset), as well as the datasets indirectly related to disease (including a microorganism co-expressed host gene set and biological processes-related datasets). Our results

showed that EnrichDO outperformed classic methods and current DO-based enrichment tools, demonstrating the effectiveness of EnrichDO for gaining insight into the significance of a particular set of genes in the context of disease. EnrichDO has been implemented as an R-based tool, which is available at https://bioconductor.org/packages/release/bioc/html/EnrichDO.html or https://github.com/liangcheng-hrbmu/EnrichDO.

Materials and Methods

Data Collection and Preprocessing

Standard DO terms were downloaded from the DO Database (http://disease-ontology.org) (data-version: releases/2024-03-28/doid.obo). The DO database is a comprehensive resource that aims to organize, represent, and standardize human diseases. The DO semantically integrates disease and medical vocabularies through extensive cross-mapping of DO terms to MeSH, ICD, NCI's thesaurus, SNOMED CT, and OMIM disease-specific terms and identifiers [4, 5]. The DO has been widely used for disease annotation by various biomedical databases [5]. The obo file records the information of 14,019 DO terms; and we collected information on 11,537 DO terms, excluding obsolete DO terms (is_obsolete=true). The names and synonyms of DO terms were extracted to construct the dictionary of disease names for DO annotation. The DOID and is_a relations were used to construct a DAG of DO.

Information on human genes with disease descriptions was downloaded from GeneRIF (version date: March 31, 2024). GeneRIF is a database that provides concise descriptions of gene function, making it the most suitable resource for DO annotation to infer gene-disease associations [23]. GeneRIF is available from the NCBI Gene Database (ftp://ftp.ncbi.nih.gov/gene/GeneRIF/). GeneRIF entries were manually extracted from scientific literature, including Tax IDs, Gene IDs, PubMed IDs, and concise GeneRIF textual descriptions (up to 250 characters) of gene function. GeneRIF entries of *Homo sapiens* were extracted from the generifs_basic file, and

GeneRIF text was annotated with DO terms.

Annotating the Human Genome with DO Terms

Semantically annotating the human genome with DO terms can connect biomedical gene and disease data through the lens of human disease. In this study, the textual descriptions of gene function in the GeneRIF database were annotated with DO terms using the University of Michigan's Mgrep tool. Mgrep is an efficient tool for mapping free text to ontology terms [35], which is used in the concept recognition step by the NCBO Annotator [11]. We choose Mgrep because it is claimed to be a fast and scalable tool for concept recognition and has a high degree of customizability vis-à-vis dictionaries and resources [36]. For concept recognition, the data resources represent a particular type of biomedical knowledge, and a dictionary represents a set of terms to recognize in the biomedical data resource. In our study, the biomedical data resources are GeneRIF entries of *Homo sapiens*; the disease dictionary was constructed by extracting all standard DO terms and their synonyms. The concept recognizer identifies the related disease name in GeneRIF entries and maps it to the concept of DO terms in the dictionary. At last, 525,289 out of 1,039,774 GeneRIF entries of *Homo sapiens* were annotated with 4,407 disease terms.

Data pre-processing and result filtering were conducted as follows: (i) the disease dictionary was generated to include all standard DO terms and synonyms, with character length no less than 3, and to exclude obsolete DO terms (is_obsolete=true); (ii) only GeneRIF entries of protein-coding genes (pc genes) of *Homo sapiens* were retained; (iii) disease names annotated to DO terms and synonyms were standardized to DOIDs; and (iv) all repeating results were removed. As a result, we acquired 191,542 gene-DO annotations, which involved 4,361 DO terms and 15,106 pc genes. The DO annotations describe unique roles for human genes in the context of disease, and can be used for DO enrichment analysis.

Semantic Expansion in DO DAG

We leveraged the hierarchical structure of DO to expand annotations. Like GO, DO has a hierarchical structure

that forms a DAG, which follows the “true-path” rule. The additional annotation information is produced using the semantic relationships in DO, such as “is_a” relation, as follows: first, DOID, name, and “is_a” relations were used to construct the DAG. Every DO term is represented by a node in the graph, while the “is_a” relation is represented by an edge in the graph. Two terms with an “is_a” relation are represented as a child node and a parent node in the graph; a child can have multiple parents. For example, “B-cell acute lymphoblastic leukemia” (DOID:0080638) is_a “acute lymphoblastic leukemia” (DOID:9952) and “lymphoma” (DOID:0060058); the node “B-cell acute lymphoblastic leukemia” is a child of “acute lymphoblastic leukemia” and “lymphoma” and, is a more specific biological classification than its parents. The term “disease” (DOID:4) is the root node of the DAG, which has no parents, and is the most general node. Next, according to the gene-DO annotations mentioned above, a set of genes annotated with each DO term in the DAG were also annotated to its parents and its ancestors (the so-called true path rule). Leaf nodes with no annotated genes were iteratively pruned, until all nodes in the final DAG tree contained at least one annotated gene. After pruning, the DAG contained 4,813 nodes. At last, all nodes of DAG were marked as different levels. For a node *n*, the level was defined as the length of the longest path from the root to node *n*. The root node, the term “disease,” was marked as level 1; level 13 is the highest level. Nodes at the same level do not share any edges and, can be investigated independently. The levels of DAG and the statistics of DO terms are displayed in Table 1.

Table 1. Annotations of molecules with ontology terms in EnrichDO.

Level	Num of DO	Num of Annotated Genes
1	1	15106
2	8	14400
3	120	15185
4	212	15526
5	574	15929
6	1242	15790
7	1009	13686
8	850	11600
9	535	7826

10	170	3231
11	58	1400
12	26	567
13	8	186

DO Enrichment based on Weighted Algorithm

Classical Enrichment Analysis

Overrepresentation analysis (ORA) is a widely used term-for-term approach for enrichment analysis. The ORA method measures the statistical proportion of a pre-selected list of genes of interest (e.g., differentially expressed gene list) and specific gene sets according to known gene functions (e.g. DO terms). The hypergeometric test is a classical ORA test, wherein the *p*-value indicates the probability of the null hypothesis, which can be calculated as follows:

$$p = 1 - \sum_{k=0}^{r-1} \frac{\binom{m}{k}\binom{N-m}{n-k}}{\binom{N}{n}} \qquad (1)$$

The value *N* is the number of all human genes annotated to DO terms, while *m* indicates the number of genes annotated to term *t* and *n* indicates the size of the interesting gene list, of which *r* are included in the term *t*. A small *p*-value indicates a low probability of randomly obtaining such statistical proportion of the interesting gene list *t*. A multiple testing correction method, such as Benjamini-Hochberg method, was then used to adjust the *p*-value to control the type I error (false positive) rate.

Weighted DO Enrichment Analysis

The disadvantage of the classical enrichment analysis approach is that it ignores the annotation dependencies between DO terms that are caused by the "true-path" rule. This inheritance problem can lead to some terms being over-enriched. To address this problem, we developed the weighted DO enrichment analysis method, referred to as EnrichDO, by double-weighting the annotated genes and integrating DO graph topology on a global scale. To reinforce the saliency of direct gene-DO annotations, we assigned different initial weights to directly annotated

genes and to indirectly annotated genes caused by the "true-path" rule, respectively. To detect the most significant local nodes between the parent and its children, we dynamically down-weighted genes in less significant nodes, as described by Alexa et al. [33].

We first define four key concepts:

(i) *Initial weight* w_i. Initially, for each DO term, the weights for all directly annotated genes are set to 1, and the weights for indirectly annotated genes decrease by 0.1 for each level inherited upward. The smaller the weights of indirectly annotated genes, the less the contribution that these genes have to the enrichment analysis. Notably, smaller weights can cause certain nodes to be missed during the enrichment analysis. In this study, the weights for indirectly annotated genes were no less than 0.5.

(ii) *Dynamic weight* w_d. For a given DO term *t* and its child *c*, the weight assigned to genes annotated to this pair of terms is defined as follows:

$$w_d = \frac{log(score(c))}{log(score(t))} \qquad (2)$$

where *score(.)* is the *p*-value of the hypergeometric test with weighted genes. The weights for genes annotated to each node are memorized and updated during the process.

(iii) *Penalty score*. The penalty score is a penalty value for gene weights other than w_d, that is used to alleviate the impact of different magnitudes of *p*-values. It is defined as follows:

$$penal = max(\frac{1}{10} \times \frac{lg(xmin)}{lg(score(t))+lg(score(c))}, 1) \qquad (3)$$

where $xmin$ is the minimum positive number in *R* and *score(.)* is the *p*-value of the hypergeometric test with weighted genes mentioned above.

(iv) *Significance score*. The significance score for a given DO term *t* is calculated by applying the hypergeometric test with weighted genes. The number *r* in formula (1), which indicates the number of interesting genes included in term *t*, is replaced by rounding down the sum of the weight of *r* genes. The number *m*, which indicates the

number of genes annotated to term *t*, is replaced by rounding down the sum of the weight of *m* genes.

EnrichDO is a double-weighted iterative model. Given a threshold of 0.01 (or less), our aim is to find significantly enriched DO terms with *p*-values < 0.01. The overall process of the algorithm is shown in Figure 1A, and the detailed process is described step-by-step as follows:

Step 1. Given the DAG with levels (mentioned in "Semantic Expansion in DO DAG"), we set initial weights for annotated genes for each node. We process the nodes bottom-up, from the highest level and then iteratively move to nodes at lower levels, with the aim of identifying the most specific nodes with minimum required significance (as shown in Figures 1A and 1B). For the current node *t*, all its children have significance scores; children with *p*-values larger than the threshold are excluded, as shown in Figures 1A and 1B. The node *t* is then processed by step 2 to step 5, as shown in Figure 1C.

Step 2. For the current node *t*, we calculate its significance score, using concept (iv) described above. If the score is larger than the threshold, then the process moves to the next node. Otherwise, *t* is compared to each of its children to discover the most significant nodes locally. In detail, for each child *c*, we calculate the weight w_d for the pair of terms. When $w_d > 1$, it indicates that *c* is more significant than *t*, thus node *c* is a local optimum, and *c* is moved from children to sigChildren; otherwise, *c* remains in children. This step was shown in Figure 1C-(1).

Step 3. For each child *c* in sigChildren, the weight of the genes annotated to *c* is decreased, by dividing term *t* and all its ancestors by w_d and the penalty score. The child *c* is then removed from sigChildren, until the sigChildren is empty. This step is shown in Figure 1C-(2).

Step 4. Steps 2-3 are executed recursively. The significant score of *t* is recalculated with updated weights and is compared to its remaining children. This process is carried out until either of the two following situations arises: (1) there are no remaining children, in which case the process moves to the next node, or (2) remaining children exist, in which case the process moves to step 5.

Step 5. For each remaining child *c*, it is less significant than *t*. Thus, node *t* is a local optimum, and the weight of genes annotated to c is decreased by multiplying w_d (w_d <1) and dividing it by the penalty value, thereby, reducing the contribution of genes to the child nodes. The significance score is then recalculated, and the process moves to the next node. This step is shown in Figure 1C-(3).

At last, the multiple testing correction procedure from the Benjamini-Hochberg method (1995) is applied to adjust the significance score (*p*-values).

Results

Summary of Annotations of the Human Genome with DO

Annotating human genes with DO is crucial to advancing the discover of gene-disease associations. In this study, GeneRIF and DO terms were utilized to annotate the human genome with DO, using the Mrep tool. We annotated 525,289 GeneRIF entries of *Homo sapiens* with 4,407 disease ontologies directly. We then integrated and filtered the annotated results, finally acquiring 191,542 gene-DO annotations with 4,361 DO terms and 15,106 pc genes. We analyzed all gene-DO annotations except for the term “disease” (DOID=4), which lacks specific significance. The number of genes annotated with each DO term was distributed from 1 to 4,730. The annotation results show an uneven distribution (Figure 2A). Out of 4,360 DO terms, 2,971 (68.14%) have fewer than 10 annotated genes, among which 1,217 specific terms have only one annotated gene. In contrast, 29 DO terms have more than 1,000 annotated genes. The DO term “cancer” (DOID=162), with the highest number of annotated genes (4,730), is a disease of uncontrolled cellular proliferation that is malignant and primary, characterized by both local cell invasion and metastasis. It is a generalized and widely studied disease located at level 4 of DAG. Specific cancer types, such as breast cancer, hepatocellular carcinoma, and colorectal cancer, are also widely studied and are annotated with more than 3,000 genes.

The number of DO terms annotated by each gene was distributed from 1 to 506, which presented an uneven

distribution, as shown in Figure 2B. Of 13,699 genes, 213 (1.55%) were annotated to more than 100 DO terms, while 9,595 genes were annotated to no more than 10 DO terms, of which 2,463 genes annotated to only one DO term. The pc gene "tumor necrosis factor (*TNF*)," which annotated with the highest number of DO terms (506), encodes a multifunctional pro-inflammatory cytokine that belongs to the TNF superfamily. This cytokine is involved in the regulation of a wide spectrum of biological processes, including cell proliferation, differentiation, apoptosis, lipid metabolism, and coagulation. It has been implicated in a variety of diseases, including autoimmune diseases, insulin resistance, psoriasis, rheumatoid arthritis ankylosing spondylitis, tuberculosis, autosomal dominant polycystic kidney disease, and cancer. Mutations in *TNF* affect susceptibility to cerebral malaria, septic shock, and Alzheimer disease [37]. Other notable genes, such as interleukin 6 (*IL6*), tumor protein p53 (*TP53*), vascular endothelial growth factor A (*VEGFA*), transforming growth factor beta 1 (*TGFB1*) and matrix metallopeptidase 9 (*MMP9*), are also implicated in a variety of diseases and annotated to more than 400 DO terms. High annotation quality is essential to the performance of enrichment analysis. We manually validated the annotations and compared them with the disease and gene annotations of the DGA database [25]. We randomly extracted and manually checked 5,000 annotation mappings, excluding the term "disease" (DOID=4). Out of 5,000 mappings, 204 were incorrectly annotated and the false positive (4.08%) was within an acceptable range, which confirmed the accuracy of the annotations of EnrichDO. In addition, we compared the annotations with the DGA database, which was widely used for DO enrichment analysis [31]. We selected four diseases with annotated gene numbers in different distribution intervals, namely Leigh disease (DOID:3652, with 53 annotated genes), retinitis pigmentosa (DOID:10584, with 158 annotated genes), colorectal carcinoma (DOID:0080199, with 549 annotated genes), and schizophrenia (DOID:5419, with 1,299 annotated genes). We then collected the manually curated disease-gene relations for these four diseases from the DisGeNET [38], MalaCards [39], and DISEASE [40] databases as standards. When comparing the directly annotated genes of Leigh disease against these three

databases, we acquired 27, 11, and 26 overlaps, respectively; overlaps with DGA were 14, 7, and 13 (see Figure 2C). Similar results were observed for retinitis pigmentosa, colorectal carcinoma, and schizophrenia. As shown in Figures 2D-2F and in Table 2, EnrichDO annotations consistently outperformed those of the DGA database in overlap counts, demonstrating the accuracy of EnrichDO annotations and their reliability for DO enrichment analysis.

Table 2. Comparison of Annotations between EnrichDO and DGA for different DO Terms.

DOID	Disease	Num of related genes in EnrichDO and DGA	Database	Num of related genes	Num of overlaps with EnrichDO	Num of overlaps with DGA
DOID:3652	Leigh disease	53/21/15	MalaCards	23	11	7
			DISEASE	63	26	13
			DisGeNET	47	27	14
DOID:10548	retinitis pigmentosa	158/126/92	MalaCards	154	89	62
			DISEASE	106	77	62
			DisGeNET	104	80	57
DOID:0080199	colorectal carcinoma	549/52/15	MalaCards	97	26	8
			DISEASE	0	0	0
			DisGeNET	702	95	12
DOID:5419	schizophrenia	1299/720/665	MalaCards	30	12	13
			DISEASE	16	13	12
			DisGeNET	883	602	448

* The three values in the third column represent the number of genes annotated with the corresponding DO term in EnrichDO and DGA, as well as the number of overlaps between EnrichDO and DGA.

Algorithm Comparison and Assessment

Comparison with Other Methods

We compared EnrichDO with the classic overrepresentation analysis method (common hypergeometric test) and topGO [33]. These two methods were widely used in the GO enrichment analysis, and thus we compared them based on both GO and DO, respectively.

We first applied three methods to perform GO (biological process, BP) enrichment analysis on acute

lymphoblastic leukemia (ALL) [41], which had been previously analyzed using topGO. The dataset consists of 95 patients with B-cell ALL and 33 patients with T-cell ALL in the study. A total of 194 differentially expressed pc genes with |logFC|>=1 were obtained for the enrichment analysis. The top 10 enriched GO terms of EnrichDO were displayed in Figure 3A. Among the top 10 results, EnrichDO had four terms that overlapped with topGO, including "T cell receptor signaling pathway" (GO:0050852), "adaptive immune response" (GO:0002250), "peptide antigen assembly with MHC class II protein complex" (GO:0002503) and "B cell activation" (GO:0042113). The top enriched GO term identified by EnrichDO was "antigen receptor-mediated signaling pathway" (GO:0050851), which was defined as the series of molecular signals initiated by the cross-linking of an antigen receptor on a B or T cell. The hypergeometric test had only one overlap with EnrichDO, named "immune response-regulating cell surface receptor signaling pathway" (GO:0002768). Additionally, GO terms such as "positive regulation of T cell activation" (GO:0050870) and "immune response-activating cell surface receptor signaling pathway" (GO:0002429) were also top-ranked in EnrichDO's results. As shown in Supplementary Table 1, the enriched terms of EnrichDO and topGO are more specific than those of the hypergeometric test. Notably, although the results of topGO are specific, they overlook the parent nodes of some significant nodes, which was more evident in the following DO enrichment analysis.

We then performed DO enrichment analysis on a publicly available dataset for Alzheimer's disease (AD), which is a neurological disorder. AD is associated with declines in thinking, memory, and language and with personality changes and changes in the brain that eventually result in the loss of ability to carry out simple daily tasks [42]. Due to the lack of a gold standard dataset for DO enrichment analysis, we collected AD-related genes from DisGeNET [38], which is deemed a gold standard dataset. We collected 91 curated disease-associated pc genes from the DisGeNET, which were taken as interesting gene set for DO enrichment analysis. All three methods were based on the latest annotations of DO from EnrichDO.

The top 10 enriched DO terms of EnrichDO are presented in Figure 3B, with ascending order of their computed *p*-values. The first-ranked disease is AD, indicating that EnrichDO can accurately enrich the specific disease. The second-ranked DO term is "tauopathy" (DOID:680), a heterogeneous group of neurodegenerative diseases characterized by aggregated tau proteins, which is the parent of AD [43]. "Mild cognitive impairment" (DOID:0080832) and "dementia" (DOID:1307) were also top-ranked. Mild cognitive impairment (MCI) is an intermediate state between normal cognition and dementia. MCI is a useful label in clinical settings to help identify individuals who are at risk of developing AD [44]. Dementia is characterized by progressive deterioration in cognition, function and behavior; AD is the most frequent cause of dementia [44]. Thus, top-10 ranked DO terms are all neurological or cognitive system diseases, indicating the accuracy of the enrichment algorithm.

Results from the comparison with the hypergeometric test and topGO on AD are displayed in Figure 3B and in Table 3. All three methods acquired low *p*-values due to the manually curated AD-related interesting genes. In the top 10 results, topGO had seven overlaps with EnrichDO's results; the DO terms "tauopathy" (2nd) and "synucleinopathy" (6th) were identified as non-significant (*p*-vlaue=1) by topGO, while "cognitive disorder" (7th) ranked 45th in the topGO results. The more specific DO terms "polycystic ovary syndrome," "type 2 diabetes mellitus," and "rheumatoid arthritis" were also among the top 10 results, but are not as closely related to AD as are the aforementioned three terms. The hypergeometric test had eight DO terms that overlapped with EnrichDO, and the top two enriched DO terms were "Alzheimer's disease" and "tauopathy." Nevertheless, DO terms ranked differently between the two methods, with the top-ranked DO terms of EnrichDO being more specific. The phenomenon is more evident in other comparisons of the significant results. Setting the threshold *p*-value < 0.01, and *p*-adjust < 0.01, the hypergeometric test identified 859 significant terms, far exceeding those identified by EnrichDO (405) and topGO (272). We investigated top 50, 100, and 200 results of the three methods in the DAG. The results of the hypergeometric test are more general, located at lower levels, and primarily converged on same

branches. Conversely, the results of topGO are more specific, located at higher levels, and distributed across distinct branches (Supplementary Table 2). The above results indicate that the hypergeometric test performed enrichment term-for-term, cannot solve over-enrichment problem, while topGO recognized more specific terms, but might overlook truly relevant nodes. In contrast, EnrichDO often yields more accurate, more specific, and more significant DO terms, thereby overcoming the over-enrichment problem. At the same time, it is also a moderate weighted algorithm that will not neglect true significant terms, even though their child nodes are significant.

Table 3. Comparison of enrichment results with other methods on AD case.

DOID	DOTerm	level	EnrichDO		ORA		topGO	
			p	rank	p	rank	p	rank
DOID:10652	Alzheimer's disease	7	2.19E-71	1	2.44E-71	1	2.44E-71	1
DOID:680	tauopathy	6	4.38E-63	2	3.89E-71	2	1	4054
DOID:0080832	mild cognitive impairment	4	3.09E-52	3	3.09E-52	4	3.09E-52	2
DOID:1307	dementia	4	3.02E-43	4	4.52E-44	5	4.52E-44	3
DOID:14330	Parkinson's disease	7	3.27E-42	5	3.48E-42	7	3.48E-42	4
DOID:0050890	synucleinopathy	6	2.26E-34	6	3.30E-41	8	1	3178
DOID:1561	cognitive disorder	3	1.26E-30	7	1.01E-43	6	5.72E-14	45
DOID:1596	depressive disorder	5	2.19E-28	8	1.40E-31	10	1.40E-31	5
DOID:2377	multiple sclerosis	8	8.99E-27	9	1.33E-27	12	1.33E-27	6
DOID:5419	schizophrenia	5	2.70E-25	10	2.78E-25	16	2.78E-25	7
DOID:1289	neurodegenerative disease	5	1.68E-22	15	8.17E-58	3	1	4348
DOID:150	disease of mental health	2	2.14E-11	107	3.61E-35	9	1	4810
DOID:1596	depressive disorder	5	2.19E-28	8	1.40E-31	10	1.40E-31	5
DOID:11612	polycystic ovary syndrome	7	3.05E-25	11	3.05E-25	17	3.05E-25	8
DOID:9352	type 2 diabetes mellitus	7	2.02E-22	16	2.07E-22	28	2.07E-22	9
DOID:7148	rheumatoid arthritis	8	2.96E-21	18	3.31E-21	33	3.31E-21	10

* ORA represents the hypergeometric test.

Comparison with Current DO-based Enrichment Analysis Tools

We also applied the AD case to compare EnrichDO with DOSE (v3.26.2), Flame (V2.0), and KOBAS-i, which

are widely used disease-based enrichment analysis tools. The enrichment tool option for Flame was set to "aGOtool," and the minGSSize and maxGSSize for DOSE were set to 5 and 5,000, respectively. All other parameters for these tools were set to default. Among the top 10 results, DOSE had four overlaps with EnrichDO. Notably, it recognized "tauopathy" as the most significantly enriched DO term, which is the parent of "Alzheimer's disease." The latter term ranked second. The parent and ancestors of the term "tauopathy," including "neurodegenerative disease," "central nervous system disease," and "nervous system disease," were also significantly enriched (Figure 4). This may be attributed to the inheritance problem caused by the "true-path" rule. DOSE identified statistically significant enriched diseases based on the hypergeometric test, which did not take topological relationships between DO terms into consideration. Another explanation for the results is that the annotation data of DOSE has not been updated and therefore contains only 10,312 annotated genes, compared to 15,106 pc genes in EnrichDO. Furthermore, DOSE obtains similar results when the enrichment background is restricted to pc genes (Supplementary Table 3). Flame had three overlaps with EnrichDO: "AD," "dementia," and "cognitive disorder." Flame recognized AD as the top correlated disease; however, in comparison to EnrichDO, other results obtained by Flame were more general, as shown in Figure 4. In addition, the branches of Flame results are more similar to DOSE (with six overlaps), and there is a branch related to metabolic diseases. KOBAS recognized five duplicate AD-like diseases, with different disease names that come from different human disease databases. The other results were mainly related to cardiovascular disease and metabolic disease. KOBAS incorporated three human disease databases, OMIM, KEGG DISEASE, and the NHGRI GWAS Catalog (NHGRI), but did not include DO. Multiple data sources might lead to redundancy in the enrichment results. The above results indicated that EnrichDO performed better than current methods.

Robustness of EnrichDO

We tested the robustness of EnrichDO with the AD dataset by removing interesting genes and by adding noise,

respectively. First, we removed the AD dataset at 5% intervals and repeated the EnrichDO method 100 times for each removal. The number of significant overlapped DO terms (top 100) decreased slowly compared to the original data, and the ratio of overlapped DO terms to original significant DO terms remained at 74.17%, even after removal of up to 30% of the AD dataset (Figure 5A). These results indicate that the EnrichDO method is robust to data removal. In a similar way, noise was added to the AD dataset. We added 5% to 30% noise from a background gene set, at 5% intervals, repeated 100 times. Significantly overlapped DO terms in the top 100 decreased from 94.8 to 86.6 (Figure 5B). The results highlight the robustness of EnrichDO to data noise.

Accuracy Assessment

The comparison and evaluation of different DO enrichment algorithms relies on true relevant DO terms. In the real datasets mentioned above, the true significant DO terms are not known. To address this problem, the simulated dataset was applied to test the accuracy of EnrichDO. The simulation study was designed as follows:

(i) *Select the known DO terms*. The number of genes directly annotated with DO terms was distributed from 1 to 4,730. DO terms with annotated genes that are too small or too large are biased. Therefore, we ordered DO terms by the number of their annotated genes and selected 20 (or 30) known DO terms randomly from the middle 50% of DO terms, which were deemed as the truly enriched nodes.

(ii) *Obtain the interesting gene list*. The genes annotated to the known DO terms were combined to a single set, which were deemed the list of interesting genes. Considering that some genes annotated with many DO terms are without specificity, and may bring noise to simulation study, genes annotated to 30 or more DO terms were removed from the list.

(iii) *Evaluate performance*. In the simulation study, the performance of the algorithms is evaluated by the number of overlaps between the identified significant DO terms and the known DO terms.

We compared EnrichDO with the hypergeometric test (based on annotations of EnrichDO) and DOSE (Figure

5C-5F). When selecting 20 known DO terms, among top 25 results, EnrichDO identified an average of 9.23 (46.15%) nodes that overlapped with known DO terms, compared with an average of 7.57 (37.85%) overlaps for the hypergeometric test and 4.39 (21.95%) overlaps for DOSE. When relaxed to the top 100 nodes, an average of 14.79, 14.73, and 7.2 (73.95%, 73.65%, and 36%) overlaps were identified by EnrichDO, the hypergeometric test, and DOSE, respectively (Figure 5C). The results indicate that EnrichDO has better performance. A simulation study of 30 selected, known DO terms was implemented in a similar manner; results are shown in Figure 5D. Notably, when the interesting gene list was derived from genes directly annotated with 20 (or 30) known DO terms, accuracy improved significantly, from 73.95% to 89.7% (or 57% to 72.93%), as shown in Figures 5E and 5F. Thus, the accuracy of EnrichDO was better than the hypergeometric test, indicating that the weighted algorithm performs better than the classic ORA enrichment methods. The hypergeometric test was better than DOSE, indicating that the accuracy of disease-gene annotations of EnrichDO is also higher than that of DOSE.

EnrichDO Application on Disease Expression Profile Dataset

EnrichDO was further applied to two real gene expression datasets of pancreatic cancer [45, 46]. The pancreatic cancer dataset I contains microarray gene-expression profiles (GSE16515), including 36 pancreatic tumor and 16 normal samples [45]. Briefly, a list of 1,380 differentially expressed genes (DEGs) were identified with a strict threshold (|logFC|>=1, p-value<0.05, q-value<0.05), of which 1,094 up-regulated DEGs were taken as the interesting genes. DO enrichment results using EnrichDO, DOSE, and the hypergeometric test are displayed in Table 4 and Figure 6A. EnrichDO identified "pancreatic cancer" (DOID:1793) as the most significantly enriched term, as did the hypergeometric test. Meanwhile, DOSE identified it as the eighth most significantly enriched term, and KOBAS as 21st, and 22nd most significantly enriched terms. Among top 10 results, EnrichDO identified many cancer types, such as "stomach cancer" (DOID:76), "colorectal cancer" (DOID:9256), "breast cancer" (DOID:1612), and "lung non-small cell carcinoma" (DOID:3908). On the one hand, the interesting gene list

contains many cancer genes, such as "*ERBB2,*" "*HMGA1,*" and "*MET,*" that are related to these cancer types, which have been widely studied. Because these cancer terms have thousands of directly annotated genes, they may be apt to enrichment. Such case also took place in DOSE and the hypergeometric test results. Furthermore, DO terms identified by DOSE and the hypergeometric test were more general; for example, "endocrine gland cancer" (DOID:170), "gastrointestinal system cancer" (DOID:3119), and "carcinoma" (DOID:305), which were ancestors of the results of EnrichDO. The above results indicate that EnrichDO has superior performance over other algorithms in real microarray gene-expression datasets.

Table 4. Comparison of enrichment analysis results of EnrichDO on pancreatic cancer case (GSE16515).

DOID	DOTerm	level	geneRatio	EnrichDO		ORA		DOSE	
				p	rank	p	rank	p	rank
DOID:1793	pancreatic cancer	6	435/1038	2.24E-92	1	4.64E-107	1	1.99E-24	8
DOID:10534	stomach cancer	6	498/1038	6.82E-90	2	3.67E-90	7	1.82E-16	20
DOID:299	adenocarcinoma	6	488/1038	6.17E-89	3	1.04E-100	2	2.42E-10	59
DOID:1324	lung cancer	6	592/1038	4.03E-85	4	5.23E-89	9	1.86E-15	24
DOID:850	lung disease	5	646/1038	2.83E-80	5	1.20E-96	4	1.47E-12	44
DOID:9256	colorectal cancer	8	564/1038	2.34E-79	6	1.09E-79	15	3.43E-09	72
DOID:1612	breast cancer	6	613/1038	4.62E-79	7	2.18E-79	18	2.08E-25	6
DOID:3908	lung non-small cell carcinoma	8	481/1038	6.27E-79	8	2.68E-81	13	3.15E-14	28
DOID:76	stomach disease	4	501/1038	9.22E-79	9	1.11E-89	8	1.10E-03	241
DOID:0050615	respiratory system cancer	5	615/1038	1.97E-77	10	9.96E-91	6	5.30E-16	22
DOID:26	pancreas disease	4	445/1038	5.78E-68	16	7.90E-100	3	8.21E-03	376
DOID:28	endocrine system disease	3	617/1038	1.72E-32	48	3.05E-91	5	2.94E-02	495
DOID:170	endocrine gland cancer	5	633/1038	9.90E-50	24	5.71E-87	10	1.66E-27	4
DOID:0050687	cell type cancer	4	814/1038	1	4618	1	4674	1.66E-35	1
DOID:0050686	organ system cancer	4	944/1038	1	4617	1	4673	7.31E-32	2
DOID:305	carcinoma	5	792/1038	1	4497	1	4606	2.23E-30	3
DOID:170	endocrine gland cancer	5	633/1038	9.90E-50	24	5.71E-87	10	1.66E-27	4
DOID:3119	gastrointestinal system cancer	5	770/1038	1	4499	1	4608	2.44E-26	5
DOID:5093	thoracic cancer	5	613/1038	2.54E-71	13	2.40E-79	19	2.38E-25	7
DOID:120	female reproductive organ cancer	6	493/1038	3.07E-56	21	6.03E-75	21	2.62E-24	9
DOID:193	reproductive	5	587/1038	1.14E-44	29	2.40E-71	24	1.00E-23	10

	organ cancer

* ORA represents the hypergeometric test.

Pancreatic cancer dataset II contains RNA-seq expression profiles (GSE119794), including 10 paired pancreatic tumor and normal samples [46]. After differential gene expression analysis, a list of 372 up-regulated DEGs were identified with a strict threshold (|logFC|>=1, p-value<0.05, q-value<0.05), which were adopted as interesting gene list. As Figure 6B shows, EnrichDO obtained enrichment results similar to those for dataset I, even though the number of interesting genes varied significantly. Among top 10 results, the terms identified by EnrichDO were more specific than the hypergeometric test, though "pancreatic cancer" ranked second in the EnrichDO results, highlighting EnrichDO's utility for RNA-seq expression dataset analysis. In contrast, DOSE identified "pancreatic cancer" as the 44th most significantly enriched term. The results show that the annotations and proposed algorithms perform better than the classic methods on real gene expression datasets.

EnrichDO Application on Other Datasets

DO Enrichment on a Microbial Host Gene Set

To explore the application of EnrichDO on additional datasets, we applied EnrichDO to the host gene set of microbes of inflammatory bowel disease (IBD). IBD, mainly in forms of Crohn's disease (CD) and ulcerative colitis (UC), is characterized by debilitating and chronic relapsing and remitting inflammation of the gastrointestinal tract or the colon; IBD exhibits significant heterogeneity at the clinical, molecular, genetic, and microbial levels [47]. Sambhawa et al. investigated the shared and disease-specific host gene-microbiome associations and identified subsets of significantly correlated host genes and gut microbes [48]. The set of host genes was used to perform DO enrichment analysis in this study. EnrichDO identified IBD (DOID:0050589) accurately, ranking first in the result list, with "colorectal cancer" (DOID:9256) ranking third. The second term was "asthma" (DOID:2841), which was found to be associated with subsequent development of IBD by Kuenzig et al. [49]. As shown in Figure 6C, the results suggest a high level of accuracy of EnrichDO when using co-

expressed host genes in microorganisms, supporting the hypothesis that host genes and gut microbial taxa involved in common biological functions act in a coordinated fashion.

DO Enrichment on Hallmark Gene Sets

EnrichDO can also be applied to investigate potential connections between diseases and specific gene sets, such as hallmark gene sets. We selected the hallmark gene sets of “inflammatory response” and “pancreas beta cells” from the Molecular Signatures Database (MSigDB) [50] and used the gene sets to execute DO enrichment analysis. For “inflammatory response,” the top 10 enriched DO terms in EnrichDO were inflammation-related diseases or immune diseases (see Table 5 and Figure 6D). For “pancreas beta cells,” the top 10 enriched DO terms in EnrichDO consisted entirely of islet-related diseases or diabetes mellitus (see Table 6 and Figure 6E). The results suggest that EnrichDO is an effective means of uncovering associations between specified gene sets and human diseases.

Table 5. The top 10 enrichment analysis results of EnrichDO on an inflammatory response case.

DOID	DOTerm	p	p.adjust	geneRatio	bgRatio
DOID:0060032	autoimmune disease of musculoskeletal system	5.04E-56	2.43E-52	129/200	2083/15106
DOID:7148	rheumatoid arthritis	4.59E-52	1.11E-48	99/200	1235/15106
DOID:848	arthritis	1.43E-45	2.30E-42	110/200	1735/15106
DOID:2841	asthma	2.96E-40	3.56E-37	76/200	905/15106
DOID:3342	bone inflammation disease	4.70E-40	4.52E-37	112/200	1819/15106
DOID:9074	systemic lupus erythematosus	8.41E-39	6.75E-36	69/200	780/15106
DOID:3393	coronary artery disease	2.96E-38	2.03E-35	82/200	1161/15106
DOID:865	vasculitis	1.95E-37	1.17E-34	53/200	385/15106
DOID:417	autoimmune disease	1.64E-36	8.76E-34	144/200	2877/15106
DOID:1176	bronchial disease	5.42E-36	2.61E-33	79/200	952/15106

Table 6. The top 10 enrichment analysis results of EnrichDO on a pancreas beta cells case.

DOID	DOTerm	p	p.adjust	geneRatio	bgRatio
DOID:11717	neonatal diabetes	5.71E-18	2.75E-14	9/39	27/15106
DOID:9351	diabetes mellitus	2.75E-15	6.61E-12	28/39	2087/15106

DOID:0050524	maturity-onset diabetes of the young	2.59E-13	4.15E-10	11/39	101/15106
DOID:10603	glucose intolerance	1.90E-12	2.28E-09	9/39	95/15106
DOID:9352	type 2 diabetes mellitus	2.44E-10	2.35E-07	22/39	1449/15106
DOID:3892	insulinoma	5.14E-10	4.13E-07	6/39	38/15106
DOID:4195	hyperglycemia	1.01E-09	6.92E-07	13/39	324/15106
DOID:0111102	maturity-onset diabetes of the young type 3	5.40E-08	3.25E-05	5/39	7/15106
DOID:2018	hyperinsulinism	6.95E-08	3.72E-05	6/39	84/15106
DOID:9993	hypoglycemia	9.20E-08	4.43E-05	6/39	89/15106

Discussion

Compared to various GO enrichment analysis methods, DO enrichment analysis methods are relatively scarce. We annotated the latest GeneRIF information with DO terms and provided a new DO Enrichment analysis method, EnrichDO, which comprehensively considers DO graph topology and double-weighting of annotated genes. EnrichDO is a moderately weighted algorithm that effectively addresses over-enrichment without pruning truly enriched nodes. EnrichDO improved the accuracy of enrichment analysis results compared to classic enrichment analysis methods and current DO-based enrichment analysis tools. Simulation and robustness tests indicate high and stable performance for EnrichDO. Additionally, we successfully applied EnrichDO to different datasets, such as a microarray gene-expression dataset, a RNA-seq expression dataset, a host gene set of microorganisms, and hallmark gene datasets.

There are some limitations of the current study. One is that the size of the interesting gene list influences the enrichment analysis results. As shown in the simulation study, when the size of known DO terms increased from 20 to 30, the average number of interesting genes (directly annotated) increased from 1,525 to 2,176, while accuracy of decreased from 89.7 to 72.93% (Figures 5E and 5F). It can be concluded that an excessively large genes-of-interest set can decrease enrichment efficiency. This phenomenon also appeared in the pancreatic cancer (GSE16515) case. By setting different thresholds of |logFC|, the number of interesting genes changed, and EnrichDO acquired better performance within the number range 67-1,094 (see Supplementary Figure S1). When

the genes-of-interest set is large, some general DO terms with too many annotated genes are enriched; examples include “stomach cancer” and “lung non-small cell carcinoma,” which may be related to pancreatic cancer. It is worth noting that some top-ranked terms, such as “stomach disease” (23/3141) and “lung disease” (119/4701), may be over-enriched, owing to inheritance problems, although EnrichDO down-weighted indirectly annotated genes. Therefore, a moderate size for the interesting gene list is suggested, as is setting a threshold for the number of annotated gene of DO terms (e.g., minNum=10, maxNum=2000). Another unexpected issue we observed is bias of annotations. Some specific DO terms have too few annotated genes to be enriched, due to a lack of available information. Out of 4,361 DO terms, 2,971 were annotated to less than 10 genes. A pipeline that automatically integrates the manually curated disease-gene relations from other databases will greatly improve the performance of EnrichDO.

In general, annotations of the human genome with DO terms and the corresponding weighted DO enrichment analysis method described herein demonstrate superior performance over existing approaches. Semantically annotating results based on the latest GeneRIF and DO terms connected data on genes and diseases through the lens of human disease. Based on these annotations, EnrichDO exhibited higher accuracy that often yielded more specific significant DO terms, which alleviated the inheritance problem, making it an effective DO enrichment tool. To facilitate the use of our model, we have developed an R-based software package, which is freely available through Bioconductor (https://bioconductor.org/packages/release/bioc/html/EnrichDO.html) or at https://github.com/liangcheng-hrbmu/EnrichDO. Background annotations will be updated annually according to the latest information in the GeneRIF and DO databases, and the EnrichDO package will be continuously maintained.

Availability of Source Code and Requirements

Project name: EnrichDO

Project home page: https://bioconductor.org/packages/release/bioc/html/EnrichDO.html, the latest version can be available at https://www.bioconductor.org/packages/devel/bioc/html/EnrichDO.html or https://github.com/liangcheng-hrbmu/EnrichDO.

Operating system(s): Platform independent

Programming language: It is recommended to use R version 4.4 and Bioconductor version 3.20 or later. For versions below R-4.4 (minimum R-4.0), users can download source packages from Bioconductor and install package(s) from local files.

Other requirements: R packages BiocGenerics, Rgraphviz, clusterProfiler, hash, S4Vectors, dplyr, ggplot2, graph, magrittr, methods, pheatmap, graphics, utils, purrr, tidyr, stats.

License: MIT

BioTools ID: biotools:enrichdo

RRID: SCR_025840

Workflowhub DOI: 10.48546/workflowhub.workflow.1221.1

Abbreviations

DO: Disease Ontology; AD: Alzheimer's disease; GO: Gene Ontology; HPO: Human Phenotype Ontology; ChEBI: Chemical Entities of Biological Interest; OAE: Ontology of Adverse Events; UMLS: Unified Medical Language System; MMTx: MetaMap Transfer tool; GeneRIF: Gene Reference Into Function; DGA: Disease and Gene Annotations database; DAG: directed acyclic graph; ORA: overrepresentation analysis; TNF: tumor necrosis factor; IL6: interleukin 6; TP53: tumor protein p53; VEGFA: vascular endothelial growth factor A; TGFB1: transforming growth factor beta 1; MMP9: matrix metallopeptidase 9; BP: biological process; ALL: acute lymphoblastic leukemia; MCI: mild cognitive impairment; NHGRI: NHGRI GWAS Catalog; DEGs: differentially

expressed genes; IBD: inflammatory bowel disease; CD: Crohn’s disease; UC: ulcerative colitis; MSigDB: Molecular Signatures Database.

Supplementary Material

Figure S1_Supplementary Material. Statistics of enrichment results according to different threshold of |logFC|.

Table S1_Supplementary Material. Comparison of enrichment analysis results with other methods on ALL case.

Authors’ contributions

L.C. conceived the idea for the manuscript; H.Y. designed the study and generated annotations for the human genome with DO terms; H.Y. and H.F. wrote the source code; H.F. conducted testing and debugging of the model. H.F., M.Z., Y.L., and C.W. performed the case study analysis and implemented the data analytics. H.Y. and Y.O.H. wrote the manuscript. L.C. revised the manuscript. All authors read and approved the final manuscript.

Funding

This work was supported by Tou-Yan Innovation Team Program of the Heilongjiang Province [2019-15]; National Natural Science Foundation of China (grant numbers 62222104, 62172130 to LC, and 61902095 to HXY), and Heilongjiang Postdoctoral Fund (LBH-Q20030 to LC).

Data availability

Annotations of the Human Genome with DO were stored in https://github.com/liangcheng-hrbmu/EnrichDO/blob/devel/data/doterms.rda.

The datasets for case studies of enrichment analysis were collected as follows:

- The dataset of ALL case [41] was obtained from the R package from Bioconductor (https://www.bioconductor.org/packages/release/data/experiment/html/ALL.html), and the differentially expressed genes were extracted.
- The dataset of the AD case was curated using genes downloaded from DisGeNET [38].

- The expression profile datasets were downloaded from the Gene Expression Omnibus (GEO) database, namely GSE16515 and GSE119794 [45, 46], and the differentially expressed genes were extracted.
- The shared and disease-specific host gene-microbiome associations were obtained from the study of Priya [48].
- The hallmark gene sets were downloaded from the Human Molecular Signatures Database [50] (MSigDB, https://www.gsea-msigdb.org/gsea/msigdb/), including HALLMARK_PANCREAS_BETA_CELLS and HALLMARK_INFLAMMATORY_RESPONSE.

Specific data information has been uploaded to https://github.com/liangcheng-hrbmu/EnrichDO/tree/devel/thesisData.

Competing Interests

The authors declare that they have no competing interests.

References

1. Alterovitz G, Xiang M, Hill DP, Lomax J, Liu J, Cherkassky M, et al. Ontology engineering. Nat Biotechnol. 2010;28 2:128-30. doi:10.1038/nbt0210-128.
2. Ashburner M, Ball CA, Blake JA, Botstein D, Butler H, Cherry JM, et al. Gene ontology: tool for the unification of biology. The Gene Ontology Consortium. Nat Genet. 2000;25 1:25-9. doi:10.1038/75556.
3. Gene Ontology C, Aleksander SA, Balhoff J, Carbon S, Cherry JM, Drabkin HJ, et al. The Gene Ontology knowledgebase in 2023. Genetics. 2023;224 1 doi:10.1093/genetics/iyad031.
4. Schriml LM, Arze C, Nadendla S, Chang YW, Mazaitis M, Felix V, et al. Disease Ontology: a backbone for disease semantic integration. Nucleic Acids Res. 2012;40 Database issue:D940-6. doi:10.1093/nar/gkr972.
5. Baron JA, Johnson CS, Schor MA, Olley D, Nickel L, Felix V, et al. The DO-KB Knowledgebase: a 20-year journey developing the disease open science ecosystem. Nucleic Acids Res. 2024;52 D1:D1305-D14. doi:10.1093/nar/gkad1051.
6. Robinson PN, Kohler S, Bauer S, Seelow D, Horn D and Mundlos S. The Human Phenotype Ontology: a tool for annotating and analyzing human hereditary disease. Am J Hum Genet. 2008;83 5:610-5. doi:10.1016/j.ajhg.2008.09.017.
7. Gargano MA, Matentzoglu N, Coleman B, Addo-Lartey EB, Anagnostopoulos AV, Anderton J, et al. The Human Phenotype Ontology in 2024: phenotypes around the world. Nucleic Acids Res. 2024;52 D1:D1333-D46. doi:10.1093/nar/gkad1005.

8. Degtyarenko K, de Matos P, Ennis M, Hastings J, Zbinden M, McNaught A, et al. ChEBI: a database and ontology for chemical entities of biological interest. Nucleic Acids Res. 2008;36 Database issue:D344-50. doi:10.1093/nar/gkm791.
9. Hastings J, Owen G, Dekker A, Ennis M, Kale N, Muthukrishnan V, et al. ChEBI in 2016: Improved services and an expanding collection of metabolites. Nucleic Acids Res. 2016;44 D1:D1214-9. doi:10.1093/nar/gkv1031.
10. He Y, Sarntivijai S, Lin Y, Xiang Z, Guo A, Zhang S, et al. OAE: The Ontology of Adverse Events. J Biomed Semantics. 2014;5:29. doi:10.1186/2041-1480-5-29.
11. Noy NF, Shah NH, Whetzel PL, Dai B, Dorf M, Griffith N, et al. BioPortal: ontologies and integrated data resources at the click of a mouse. Nucleic Acids Res. 2009;37 Web Server issue:W170-3. doi:10.1093/nar/gkp440.
12. Whetzel PL, Noy NF, Shah NH, Alexander PR, Nyulas C, Tudorache T, et al. BioPortal: enhanced functionality via new Web services from the National Center for Biomedical Ontology to access and use ontologies in software applications. Nucleic Acids Res. 2011;39 Web Server issue:W541-5. doi:10.1093/nar/gkr469.
13. Smith B, Ashburner M, Rosse C, Bard J, Bug W, Ceusters W, et al. The OBO Foundry: coordinated evolution of ontologies to support biomedical data integration. Nat Biotechnol. 2007;25 11:1251-5. doi:10.1038/nbt1346.
14. Jackson R, Matentzoglu N, Overton JA, Vita R, Balhoff JP, Buttigieg PL, et al. OBO Foundry in 2021: operationalizing open data principles to evaluate ontologies. Database (Oxford). 2021;2021 doi:10.1093/database/baab069.
15. Carbon S, Ireland A, Mungall CJ, Shu S, Marshall B, Lewis S, et al. AmiGO: online access to ontology and annotation data. Bioinformatics. 2009;25 2:288-9. doi:10.1093/bioinformatics/btn615.
16. Thomas PD, Hill DP, Mi H, Osumi-Sutherland D, Van Auken K, Carbon S, et al. Gene Ontology Causal Activity Modeling (GO-CAM) moves beyond GO annotations to structured descriptions of biological functions and systems. Nat Genet. 2019;51 10:1429-33. doi:10.1038/s41588-019-0500-1.
17. Yu G, Li F, Qin Y, Bo X, Wu Y and Wang S. GOSemSim: an R package for measuring semantic similarity among GO terms and gene products. Bioinformatics. 2010;26 7:976-8. doi:10.1093/bioinformatics/btq064.
18. Thomas PD, Ebert D, Muruganujan A, Mushayahama T, Albou LP and Mi H. PANTHER: Making genome-scale phylogenetics accessible to all. Protein Sci. 2022;31 1:8-22. doi:10.1002/pro.4218.
19. Zemojtel T, Kohler S, Mackenroth L, Jager M, Hecht J, Krawitz P, et al. Effective diagnosis of genetic disease by computational phenotype analysis of the disease-associated genome. Sci Transl Med. 2014;6 252:252ra123. doi:10.1126/scitranslmed.3009262.
20. Li J, Gong B, Chen X, Liu T, Wu C, Zhang F, et al. DOSim: an R package for similarity between diseases based on Disease Ontology. BMC Bioinformatics. 2011;12:266. doi:10.1186/1471-2105-12-266.
21. Cheng L, Li J, Ju P, Peng J and Wang Y. SemFunSim: a new method for measuring disease similarity by integrating semantic and gene functional association. PLoS One. 2014;9 6:e99415. doi:10.1371/journal.pone.0099415.

22. Hu Y, Zhao L, Liu Z, Ju H, Shi H, Xu P, et al. DisSetSim: an online system for calculating similarity between disease sets. J Biomed Semantics. 2017;8 Suppl 1:28. doi:10.1186/s13326-017-0140-2.
23. Osborne JD, Flatow J, Holko M, Lin SM, Kibbe WA, Zhu LJ, et al. Annotating the human genome with Disease Ontology. BMC Genomics. 2009;10 Suppl 1 Suppl 1:S6. doi:10.1186/1471-2164-10-s1-s6.
24. LePendu P, Musen MA and Shah NH. Enabling enrichment analysis with the Human Disease Ontology. J Biomed Inform. 2011;44 Suppl 1 Suppl 1:S31-s8. doi:10.1016/j.jbi.2011.04.007.
25. Peng K, Xu W, Zheng J, Huang K, Wang H, Tong J, et al. The Disease and Gene Annotations (DGA): an annotation resource for human disease. Nucleic Acids Res. 2013;41 Database issue:D553-60. doi:10.1093/nar/gks1244.
26. Bu D, Luo H, Huo P, Wang Z, Zhang S, He Z, et al. KOBAS-i: intelligent prioritization and exploratory visualization of biological functions for gene enrichment analysis. Nucleic Acids Res. 2021;49 W1:W317-W25. doi:10.1093/nar/gkab447.
27. Kuleshov MV, Jones MR, Rouillard AD, Fernandez NF, Duan Q, Wang Z, et al. Enrichr: a comprehensive gene set enrichment analysis web server 2016 update. Nucleic Acids Res. 2016;44 W1:W90-7. doi:10.1093/nar/gkw377.
28. Elizarraras JM, Liao Y, Shi Z, Zhu Q, Pico AR and Zhang B. WebGestalt 2024: faster gene set analysis and new support for metabolomics and multi-omics. Nucleic Acids Res. 2024;52 W1:W415-W21. doi:10.1093/nar/gkae456.
29. Karatzas E, Baltoumas FA, Aplakidou E, Kontou PI, Stathopoulos P, Stefanis L, et al. Flame (v2.0): advanced integration and interpretation of functional enrichment results from multiple sources. Bioinformatics. 2023;39 8 doi:10.1093/bioinformatics/btad490.
30. Scholz C, Lyon D, Refsgaard JC, Jensen LJ, Choudhary C and Weinert BT. Avoiding abundance bias in the functional annotation of post-translationally modified proteins. Nat Methods. 2015;12 11:1003-4. doi:10.1038/nmeth.3621.
31. Yu G, Wang LG, Yan GR and He QY. DOSE: an R/Bioconductor package for disease ontology semantic and enrichment analysis. Bioinformatics. 2015;31 4:608-9. doi:10.1093/bioinformatics/btu684.
32. Amar D, Vizel A, Levy C and Shamir R. ADEPTUS: a discovery tool for disease prediction, enrichment and network analysis based on profiles from many diseases. Bioinformatics. 2018;34 11:1959-61. doi:10.1093/bioinformatics/bty027.
33. Alexa A, Rahnenfuhrer J and Lengauer T. Improved scoring of functional groups from gene expression data by decorrelating GO graph structure. Bioinformatics. 2006;22 13:1600-7. doi:10.1093/bioinformatics/btl140.
34. Grossmann S, Bauer S, Robinson PN and Vingron M. Improved detection of overrepresentation of Gene-Ontology annotations with parent child analysis. Bioinformatics. 2007;23 22:3024-31. doi:10.1093/bioinformatics/btm440.
35. Dai M, Shah NH, Xuan W, Musen MA, Watson SJ, Athey BD, et al. An efficient solution for mapping free text to ontology terms. AMIA summit on translational bioinformatics. 2008;21.
36. Shah NH, Bhatia N, Jonquet C, Rubin D, Chiang AP and Musen MA. Comparison of concept recognizers for building the Open Biomedical Annotator. BMC Bioinformatics.

2009;10 Suppl 9 Suppl 9:S14. doi:10.1186/1471-2105-10-S9-S14.
37. Annibaldi A and Meier P. Checkpoints in TNF-Induced Cell Death: Implications in Inflammation and Cancer. Trends Mol Med. 2018;24 1:49-65. doi:10.1016/j.molmed.2017.11.002.
38. Pinero J, Ramirez-Anguita JM, Sauch-Pitarch J, Ronzano F, Centeno E, Sanz F, et al. The DisGeNET knowledge platform for disease genomics: 2019 update. Nucleic Acids Res. 2020;48 D1:D845-D55. doi:10.1093/nar/gkz1021.
39. Rappaport N, Twik M, Plaschkes I, Nudel R, Iny Stein T, Levitt J, et al. MalaCards: an amalgamated human disease compendium with diverse clinical and genetic annotation and structured search. Nucleic Acids Res. 2017;45 D1:D877-D87. doi:10.1093/nar/gkw1012.
40. Grissa D, Junge A, Oprea TI and Jensen LJ. Diseases 2.0: a weekly updated database of disease-gene associations from text mining and data integration. Database (Oxford). 2022;2022 doi:10.1093/database/baac019.
41. Chiaretti S, Li X, Gentleman R, Vitale A, Vignetti M, Mandelli F, et al. Gene expression profile of adult T-cell acute lymphocytic leukemia identifies distinct subsets of patients with different response to therapy and survival. Blood. 2004;103 7:2771-8. doi:10.1182/blood-2003-09-3243.
42. Ballard C, Gauthier S, Corbett A, Brayne C, Aarsland D and Jones E. Alzheimer's disease. Lancet. 2011;377 9770:1019-31. doi:10.1016/s0140-6736(10)61349-9.
43. Horie K, Barthélemy NR, Spina S, VandeVrede L, He Y, Paterson RW, et al. CSF tau microtubule-binding region identifies pathological changes in primary tauopathies. Nat Med. 2022;28 12:2547-54. doi:10.1038/s41591-022-02075-9.
44. Reitz C and Mayeux R. Alzheimer disease: epidemiology, diagnostic criteria, risk factors and biomarkers. Biochem Pharmacol. 2014;88 4:640-51. doi:10.1016/j.bcp.2013.12.024.
45. Pei H, Li L, Fridley BL, Jenkins GD, Kalari KR, Lingle W, et al. FKBP51 affects cancer cell response to chemotherapy by negatively regulating Akt. Cancer Cell. 2009;16 3:259-66. doi:10.1016/j.ccr.2009.07.016.
46. Lin J, Wu YJ, Liang X, Ji M, Ying HM, Wang XY, et al. Network-based integration of mRNA and miRNA profiles reveals new target genes involved in pancreatic cancer. Mol Carcinog. 2019;58 2:206-18. doi:10.1002/mc.22920.
47. Adolph TE and Zhang J. Diet fuelling inflammatory bowel diseases: preclinical and clinical concepts. Gut. 2022;71 12:2574-86. doi:10.1136/gutjnl-2021-326575.
48. Priya S, Burns MB, Ward T, Mars RAT, Adamowicz B, Lock EF, et al. Identification of shared and disease-specific host gene-microbiome associations across human diseases using multi-omic integration. Nat Microbiol. 2022;7 6:780-95. doi:10.1038/s41564-022-01121-z.
49. Kuenzig ME, Barnabe C, Seow CH, Eksteen B, Negron ME, Rezaie A, et al. Asthma Is Associated With Subsequent Development of Inflammatory Bowel Disease: A Population-based Case-Control Study. Clin Gastroenterol Hepatol. 2017;15 9:1405-12 e3. doi:10.1016/j.cgh.2017.02.042.
50. Castanza AS, Recla JM, Eby D, Thorvaldsdottir H, Bult CJ and Mesirov JP. Extending support for mouse data in the Molecular Signatures Database (MSigDB). Nat Methods.

2023;20 11:1619-20. doi:10.1038/s41592-023-02014-7.

Figure Titles and Legends

Figure 1. Flowchart of EnrichDO. (A) The main process of EnrichDO. (B) The nodes of the DAG were processed bottom-up, and the children of node t were divided into different sets according to their significance scores. (C) Each node t was processed in detail step by step.

Figure 2. Statistics and Comparisons of Annotations of the Human Genome with DO. (A) Statistics of genes annotated with each DO term. (B) Statistics of DO terms annotated to each gene. (C) Annotations of Leigh disease in different databases. (D) Annotations of retinitis pigmentosa in different databases. (E) Annotations of colorectal carcinoma in different databases. (F) Annotations of schizophrenia in different databases.

Figure 3. Comparison of EnrichDO with other methods. (A) Comparison of GO enrichment analysis of EnrichDO, topGO and the hypergeometric test, on ALL case. (B) Comparison of DO enrichment analysis of EnrichDO, topGO and the hypergeometric test, on AD case.

Figure 4. Comparison of Enrichment results of EnrichDO with DOSE and Flame on AD case.

Figure 5. Stability and Accuracy Assessment of EnrichDO. (A) (B) Stability assessment of EnrichDO by random deletion test and noise addition test on AD case. (C) (D) Accuracy assessment of EnrichDO with simulated dataset (semantic expansion genes) of 20 and 30 known DO terms. (E) (F) Accuracy assessment of EnrichDO with simulated dataset (directly annotated genes) of 20 and 30 known DO terms.

Figure 6. Enrichment results of various datasets. (A) Top 10 enrichment results of EnrichDO, ORA (hypergeometric test) and DOSE for microarray gene-expression profiles (GSE16515); (B) Top 10 enrichment results of EnrichDO, ORA (hypergeometric test) and DOSE for RNA-seq expression profiles (GSE119794); (C) Top 10 enrichment results of EnrichDO for host genes of microbes of IBD; (D) Top 10 enrichment results of EnrichDO for hallmark gene sets of “inflammatory response”; (E) Top 10 enrichment results of EnrichDO for hallmark gene sets of “pancreas beta cells”.

Figure 1

Click here to access/download;Figure;Fig1.tiff

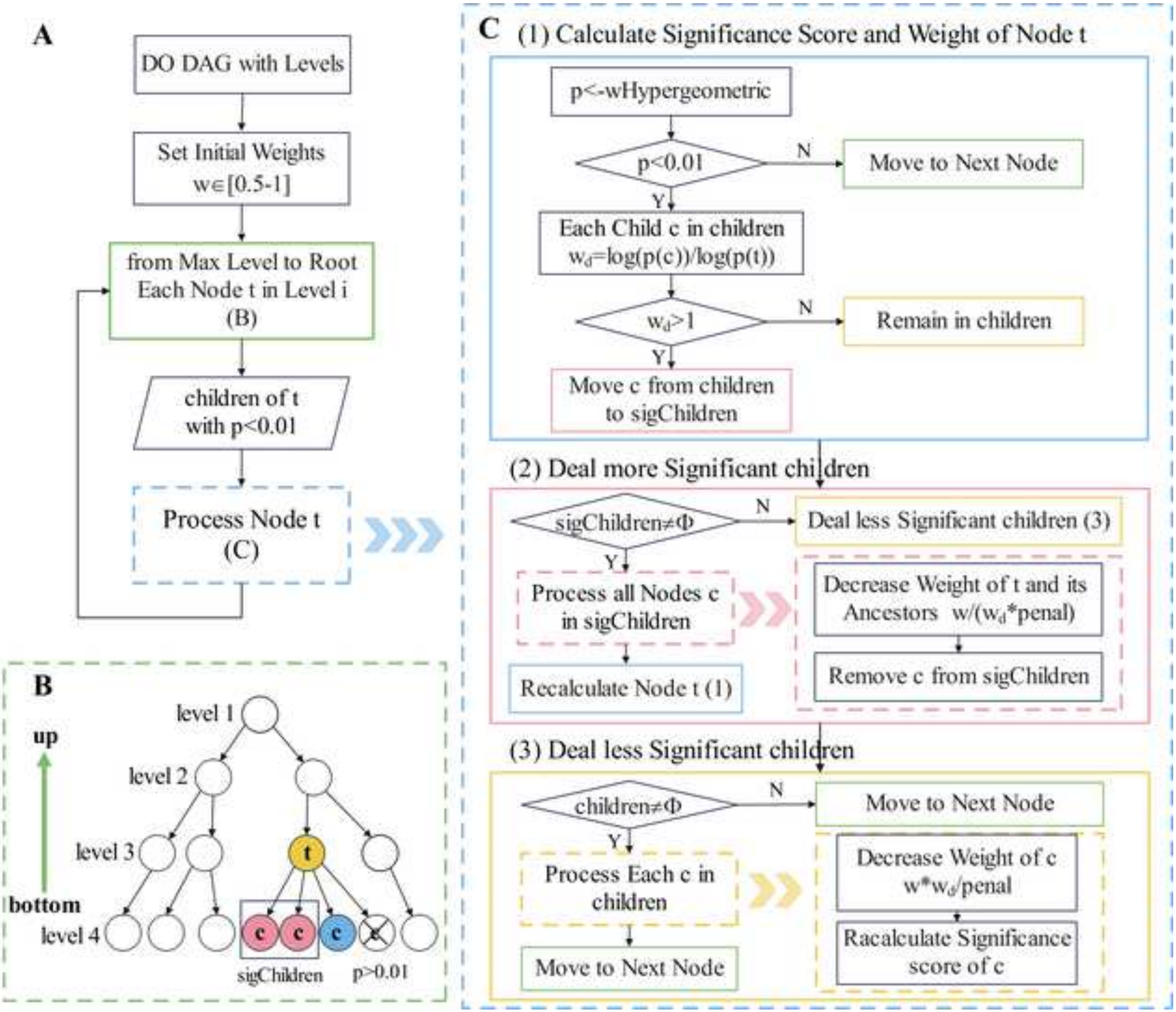

Figure 2

Click here to access/download;Figure;Fig2.tif

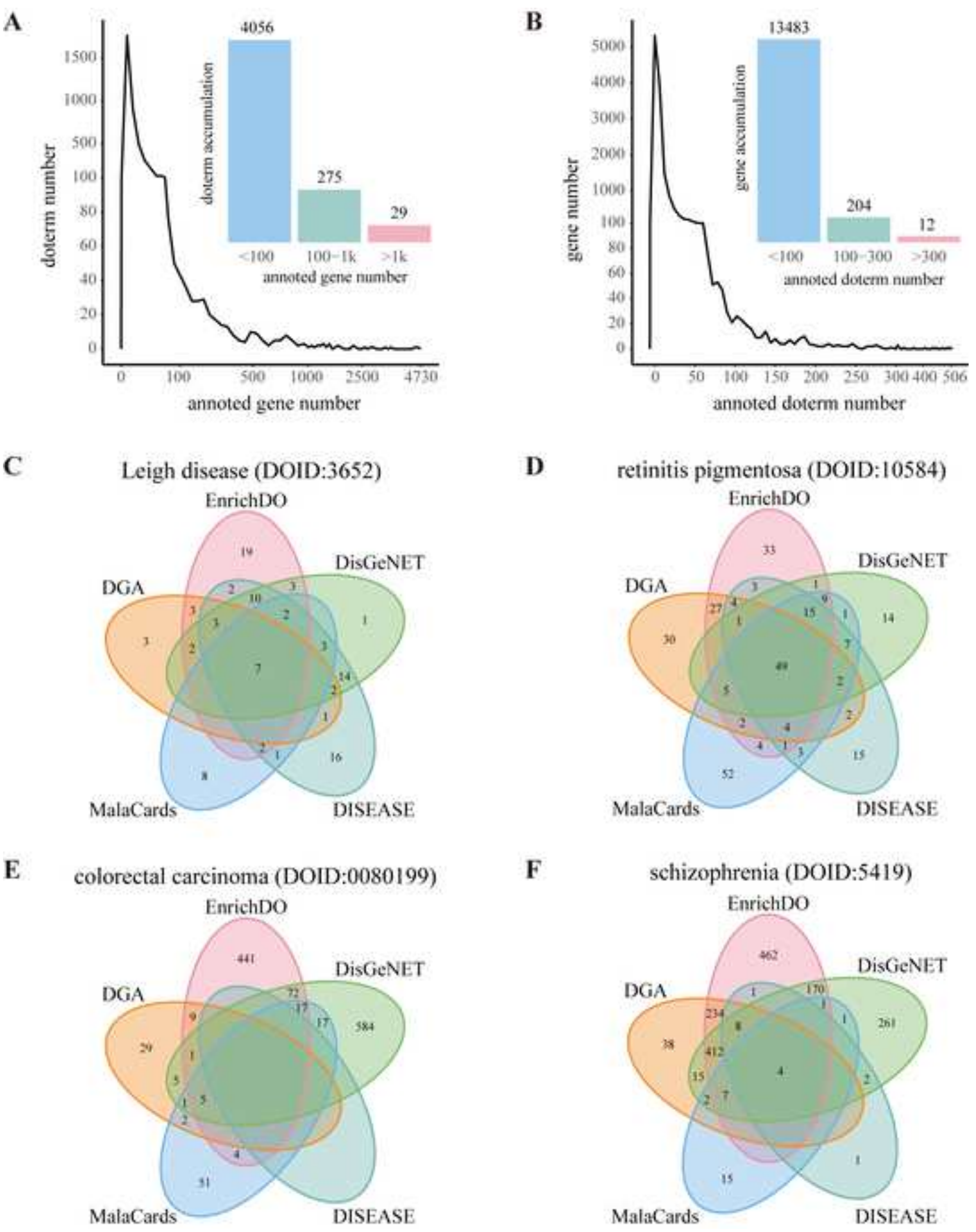

Figure 3

Click here to access/download;Figure;Fig3.tif

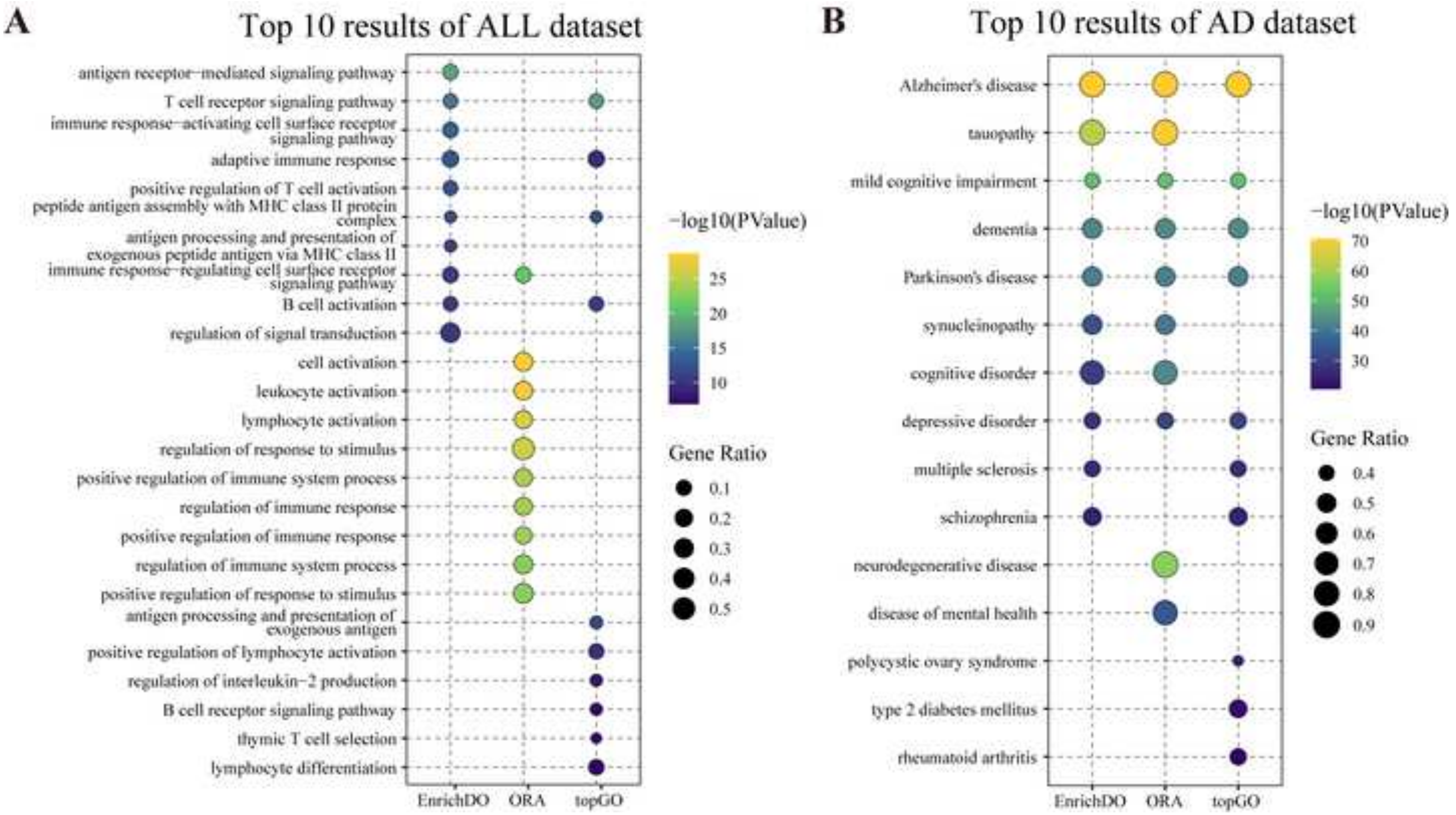

Figure 4

Click here to access/download;Figure;Fig4.tif

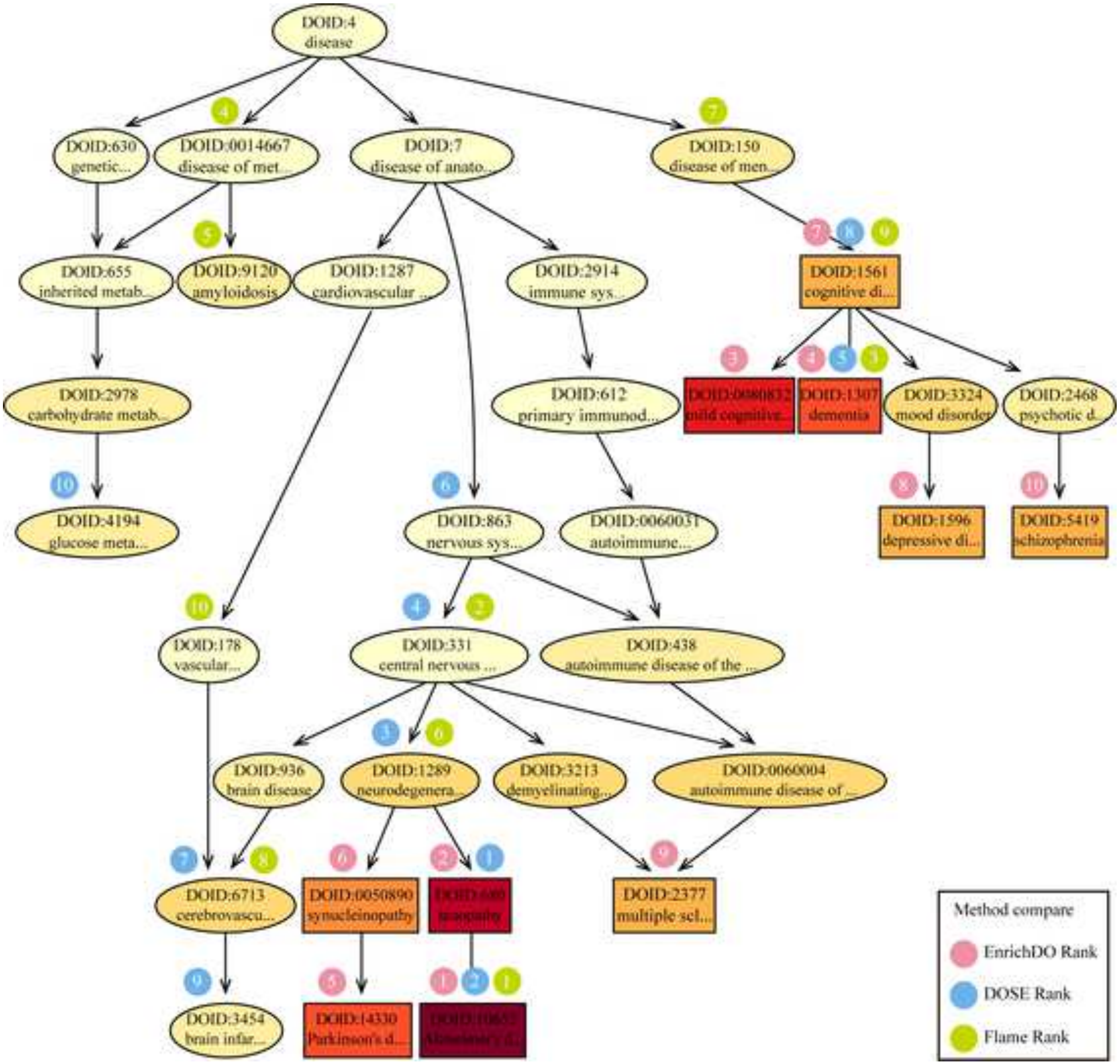

Figure 5

Click here to access/download;Figure;Fig5.tif

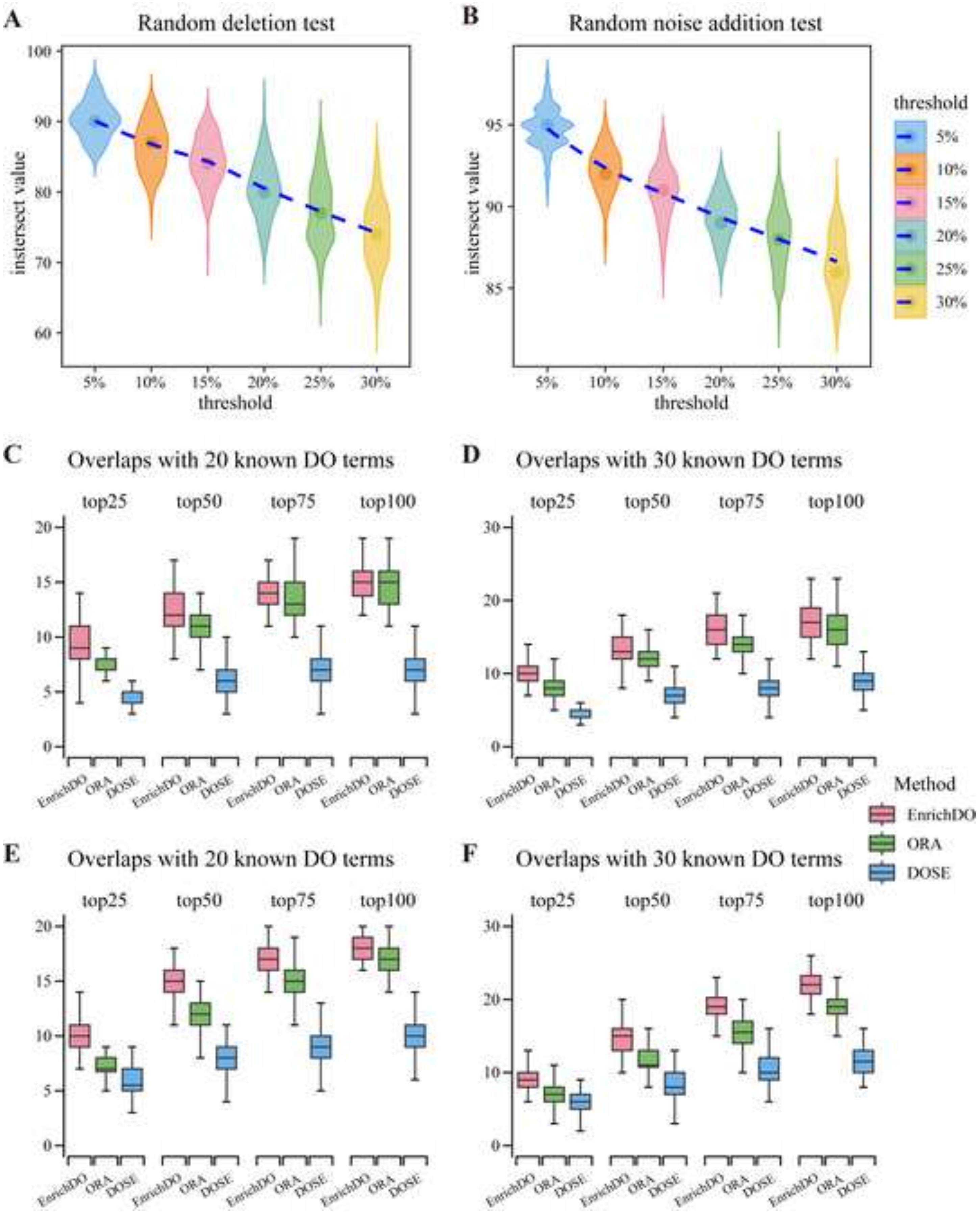

Figure 6

Click here to access/download;Figure;Fig6.tif

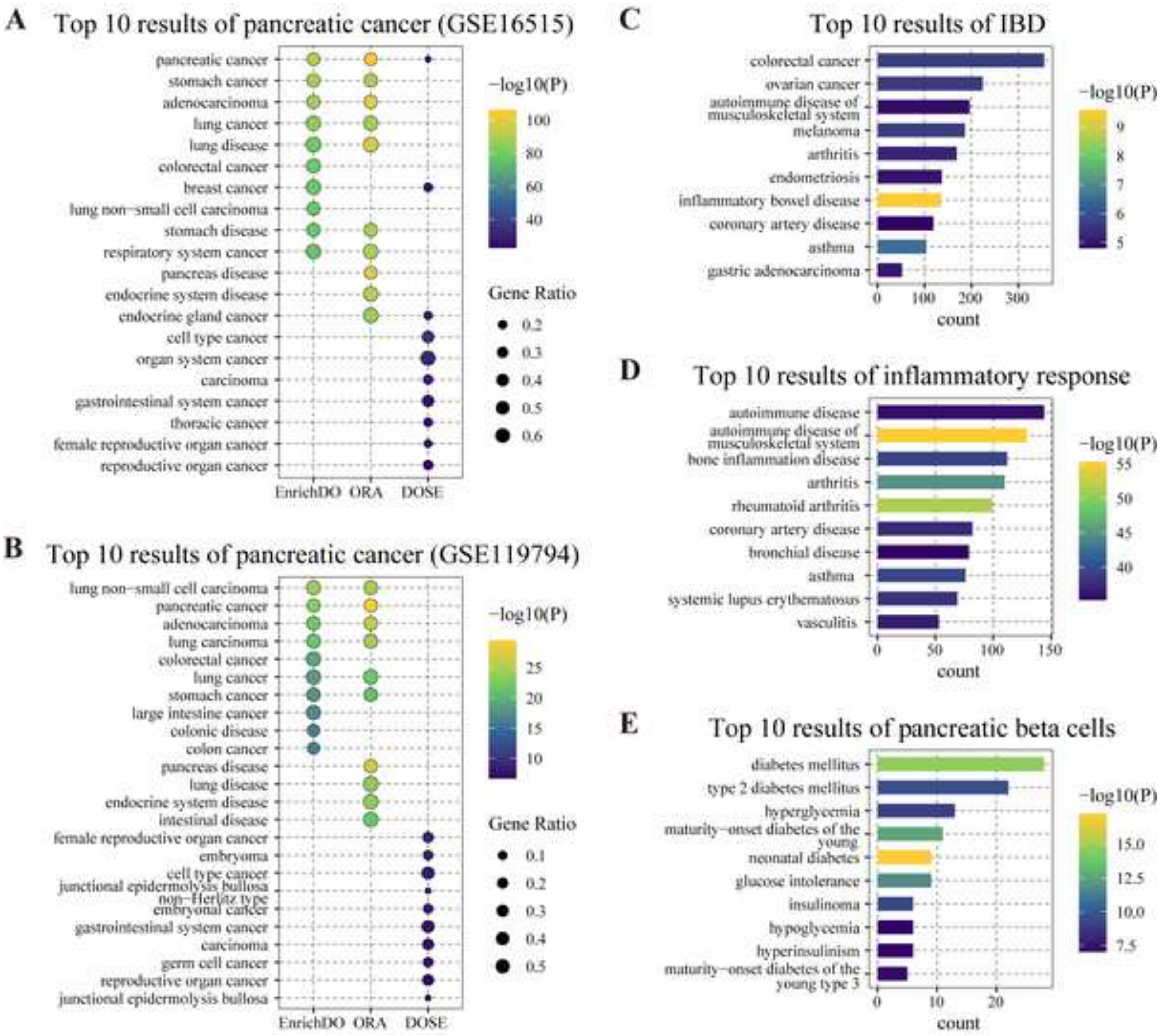

Click here to access/download
Supplementary Material
Figure S1_Supplementary Material.pdf

Manuscript

Click here to access/download
Supplementary Material
Supplementary Materials.docx

Graphical Abstract

Click here to access/download;Graphical Abstract;Graphical Abstract.tif

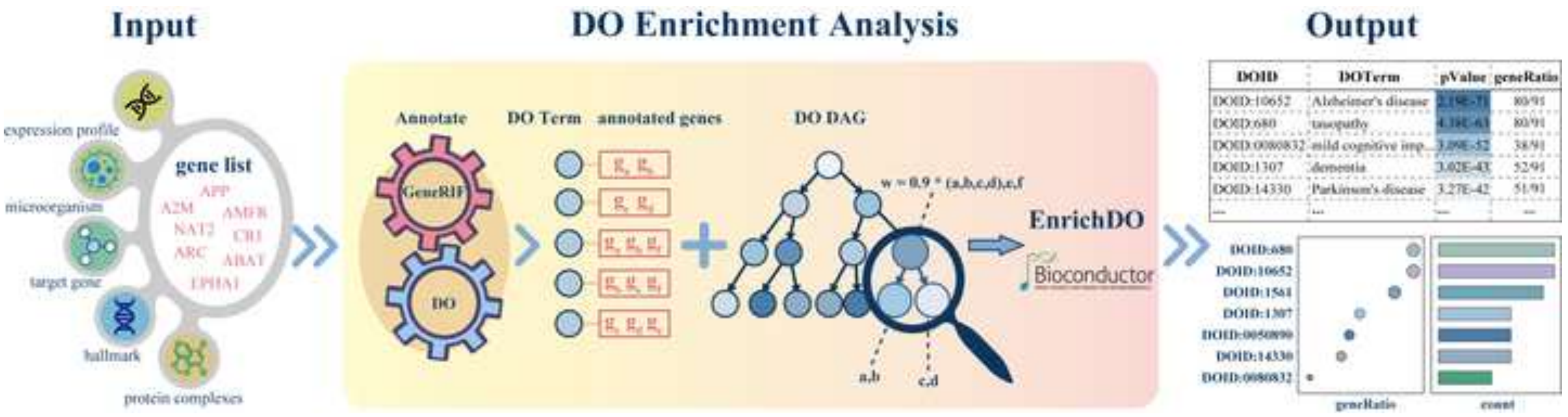

Supplement: giaf021_GIGA-D-24-00357_Revision_1 [file giaf021_giga-d-24-00357_revision_1.pdf]
