# Supplementary material for: EnrichDO: a global weighted model for Disease Ontology enrichment analysis
Source: Gigascience. 2025 Mar 26;14:giaf021. doi: 10.1093/gigascience/giaf021 (PMC11945307; doi:10.1093/gigascience/giaf021)
Supplement: giaf021_Response_to_Reviewer_Comments_Original_Submission [file giaf021_response_to_reviewer_comments_original_submission.pdf]

Author's Response To Reviewer Comments

Dear Prof. Nicole Nogoy and reviewers,
Thanks very much for your editorial efforts regarding our manuscript entitled “EnrichDO: a Global Weighted Model for Disease Ontology Enrichment Analysis” (Manuscript ID: GIGA-D-24-00357). We also thank the reviewers for their constructive comments to strengthen this manuscript. Those comments are all valuable and very helpful for revising and improving our paper, as well as the important guiding significance to our research. We have studied comments carefully and have made corrections which we hope meet with approval. Revised portion are marked in RED in the paper. The main corrections in the paper and the responses to the reviewer’s comments are as following:

Reply to Editor
In addition, please register any new software application in the bio.tools and SciCrunch.org databases to receive RRID (Research Resource Identification Initiative ID) and biotoolsID identifiers, and include these in your manuscript. Computational workflows should be registered in workflowhub.eu and the DOIs cited in the relevant places in the manuscript. These will facilitate tracking, reproducibility and re-use of your tool. Please include a point-by-point within the 'Response to Reviewers' box in the submission system. Please ensure you describe additional experiments that were carried out and include a detailed rebuttal of any criticisms or requested revisions that you disagreed with. Please also ensure that your revised manuscript conforms to the journal style, which can be found in the Instructions for Authors on the journal homepage. If the data and code has been modified in the revision process, please be sure to update the public versions of this too.

Response：Thank you very much for your editorial efforts regarding our manuscript. In response to your request, we have registered our software on the bio.tools and SciCrunch.org databases and added RRID and biotoolsID identifiers to the section “Availability of Source Code and Requirements” in main manuscript. We have also registered the computational workflows in workflowhub.eu and added the DOIs to the section “Availability of Source Code and Requirements” in main manuscript.
We have provided detailed responses to all the reviewers' comments. We confirmed that the modifications and additional experimental results have been included in the main manuscript and supplementary data as required. In addition, we have adjusted the overall style of the manuscript to ensure compliance with the journal's "Author Guidelines" section. We believe that the manuscript has been greatly strengthened by the critique of the reviewers and hope that both you and the reviewers will now find the paper suitable for publication.

Reply to Reviewer #1

Comments to the Author:
In the manuscript entitled "EnrichDO: a Global Weighted Model for Disease Ontology Enrichment Analysis", authors described a new method and a tool for DO enrichment analysis by considering the topology of DO. However, my comment is the manuscript is not clearly written, the methods are not clearly explained and the analysis is not convincing.

Response：Many thanks to the reviewer for carefully reviewing our work. Those constructive comments are all valuable and very helpful for revising and improving our paper. We have studied comments carefully and have made corrections which we hope meet with approval. In the revised manuscript, major revisions are shown in RED color.

Point 1: ## The language
I am very flexible to the English writing when I review manuscripts because I am not a native English speaker either. But in this manuscript, authors should spend more time to improve their English. There are many mistakes and unnatural uses of words and grammar. For example, in the Abstract, "DO-based methods were relatively scare,", scare? I guess it should be "rare"?

Response 1: Many thanks to the reviewer for pointing this out. We apologize for using this word incorrectly. To improve the clarity and readability, the manuscript has been professionally edited by the Open-Science publishing company, GigaScience Press, to correct academic and grammatical errors. All authors have also carefully revised the manuscript version to improve its overall clarity and readability.

Page 1, section “Abstract” in the main manuscript.
“Compared to hundreds of Gene Ontology (GO)-based enrichment analysis methods, however, DO-based methods are relatively scarce, and most current DO-based approaches are term-for-term and thus are unable to solve over-enrichment problems caused by the “true-path” rule.”

Point 2: ## Annotating the Human Genome with DO Terms
Authors link DO terms to genes by using an ontology annotator from gene-related textual descriptions. Have authors validated the quality of such mapping? In the manuscript, authors claimed that their annotation is better because there are more genes annotated to DO terms. There might be false positives in the mapping. The validation can be easily done by manually checking e.g. 1000 mappings, which, according to my experience, only takes 1-2 hours' work.

Response 2: Thank you for your invaluable comment. We accepted reviewer’s advice and validated the quality of the annotations. We randomly extracted 5,000 mappings, except for the root node “disease (DOID=4)” because of its’ lack of specific significance, and manually checked these mappings. Out of 5,000 mappings, there are 4,796 confirmed annotation relationships and 204 incorrect annotations.
In this study, the annotations were obtained by annotating the textual descriptions of gene function (GeneRIF) with DO terms utilizing Mgrep. Mgrep is an efficient tool designed for mapping free text to ontology terms, which was used by NCBO Annotator service. In this study, the false positive is 4.08%, which is within an acceptable range.
We have added this manual validation content to the section “Summary of Annotations of the Human Genome with DO” in main manuscript.

Page 12, section “Summary of Annotations of the Human Genome with DO” in the main manuscript.
“High annotation quality is essential to the performance of enrichment analysis. We manually validated the annotations and compared them with the disease and gene annotations of the DGA database [25]. We randomly extracted and manually checked 5,000 annotation mappings, excluding the term “disease” (DOID=4). Out of 5,000 mappings, 204 were incorrectly annotated and the false positive (4.08%) was within an acceptable range, which confirmed the accuracy of the annotations of EnrichDO.”

Point 3: ## Weighted DO Enrichment Analysis
(1) Shouldn't step iv be the the second step because it is actually related to how to calculate score(c) and score(t)?
(2) I don't understand equation 3, and its functions in the adjustment process in this method.
(3) How does it deal with nodes with multiple parents?

Response 3: We apologize for the unclear method description about the section “Weighted DO Enrichment Analysis”. We have revised this section in main manuscript to make the algorithm’s process clearer.
(1) We first define four key concepts: (i) initial weight wi; (ii) dynamic weight wd; (iii) penalty score; (iv) significance score. Then we described the algorithm process through five steps. In step 2, we first calculated the significance score for the current node t using concept mentioned in (iv), then calculated the dynamic weights wd for t and each child of t (mentioned in (ii)), and divided the children into two groups according to wd.
(2) Equation 3 is a penalty value for gene weights other than wd. We defined the penalty score based on a large number of experimental results, and used it to alleviate the impact of different magnitudes of p-values. As described in concepts (ii), the dynamic weights was defined as
$w_d=(\log(score(c)))/(\log(score(t)))$, which score(.) is the p-value of weighted hypergeometric test.
Given two groups, (1) score(c1)=1e-1, score(t1)=1e-2; (2) score(c2)=1e-25, score(t2)=1e-50; the wd was both 0.5, thus the weight of genes of child nodes for two groups is the same. However, the magnitudes of the two groups’ p-value are significantly different, and it is inappropriate to down-weight the child nodes for two groups equally. Thus we further down-weighted the child nodes according to the

significance of the parent and child nodes as shown in equation 3.
(3) Node with multiple parents can be operated normally according to the algorithm process, as we mainly focus on comparing the current node with its children. For the current node t, when the child node c is more significant than t, the genes of c in node t and all its ancestors (including multiple parents of t) should be down-weighted. While the other parent nodes of c do not participate in the current calculation. We have revised the section "Weighted DO Enrichment Analysis" in main manuscript.

Page 9-11, section "Weighted DO Enrichment Analysis" in the main manuscript.
"We first define four key concepts:
(i) Initial weight wi. Initially, for each DO term, the weights for all directly annotated genes are set to 1, and the weights for indirectly annotated genes decrease by 0.1 for each level inherited upward. The smaller the weights of indirectly annotated genes, the less the contribution that these genes have to the enrichment analysis. Notably, smaller weights can cause certain nodes to be missed during the enrichment analysis. In this study, the weights for indirectly annotated genes were no less than 0.5.
(ii) Dynamic weight wd. For a given DO term t and its child c, the weight assigned to genes annotated to this pair of terms is defined as follows:
$$w_d=(\log(score(c)))/(\log(score(t))) \quad (2)$$
where score(.) is the p-value of the hypergeometric test with weighted genes. The weights for genes annotated to each node are memorized and updated during the process.
(iii) Penalty score. The penalty score is a penalty value for gene weights other than wd, that is used to alleviate the impact of different magnitudes of p-values. It is defined as follows:
$$penal=\max(1/10\times \lg(xmin)/(\lg(score(t))+\lg(score(c))),1) \quad (3)$$
where xmin is the minimum positive number in R and score(.) is the p-value of the hypergeometric test with weighted genes mentioned above.
(iv) Significance score. The significance score for a given DO term t is calculated by applying the hypergeometric test with weighted genes. The number r in formula (1), which indicates the number of interesting genes included in term t, is replaced by rounding down the sum of the weight of r genes. The number m, which indicates the number of genes annotated to term t, is replaced by rounding down the sum of the weight of m genes.
EnrichDO is a double-weighted iterative model. Given a threshold of 0.01 (or less), our aim is to find significantly enriched DO terms with p-values < 0.01. The overall process of the algorithm is shown in Figure 1A, and the detailed process is described step-by-step as follows:
Step 1. Given the DAG with levels (mentioned in "Semantic Expansion in DO DAG"), we set initial weights for annotated genes for each node. We process the nodes bottom-up, from the highest level and then iteratively move to nodes at lower levels, with the aim of identifying the most specific nodes with minimum required significance (as shown in Figures 1A and 1B). For the current node t, all its children have significance scores; children with p-values larger than the threshold are excluded, as shown in Figures 1A and 1B. The node t is then processed by step 2 to step 5, as shown in Figure 1C.
Step 2. For the current node t, we calculate its significance score, using concept (iv) described above. If the score is larger than the threshold, then the process moves to the next node. Otherwise, t is compared to each of its children to discover the most significant nodes locally. In detail, for each child c, we calculate the weight wd for the pair of terms. When wd >1, it indicates that c is more significant than t, thus node c is a local optimum, and c is moved from children to sigChildren; otherwise, c remains in children. This step was shown in Figure 1C-(1).
Step 3. For each child c in sigChildren, the weight of the genes annotated to c is decreased, by dividing term t and all its ancestors by wd and the penalty score. The child c is then removed from sigChildren, until the sigChildren is empty. This step is shown in Figure 1C-(2).
Step 4. Steps 2-3 are executed recursively. The significant score of t is recalculated with updated weights and is compared to its remaining children. This process is carried out until either of the two following situations arises: (1) there are no remaining children, in which case the process moves to the next node, or (2) remaining children exist, in which case the process moves to step 5.
Step 5. For each remaining child c, it is less significant than t. Thus, node t is a local optimum, and the weight of genes annotated to c is decreased by multiplying wd (wd <1) and dividing it by the penalty value, thereby, reducing the contribution of genes to the child nodes. The significance score is then recalculated, and the process moves to the next node. This step is shown in Figure 1C-(3)."

Point 4: ## Comparison with Other Methods

(1) topGO by default also uses ORA, so it is actually the same as ORA in the comparison. Authors can see it in Table 3 where ORA and topGO give the same p-values.
I noticed DO terms 2, 6, 11 and 12 have p-value of 1 by topGO. What is the reason for that? Is it because topGO has a different cutoff for minimal number of genes in a gene set?
(2) Only compare top 10 terms is not sufficient to conclude which tool is better than others. As authors can see in the tables, these top significant gene sets are with very small p-values. Under this context, the ranking of gene sets is less important because they are "all significant and important".
(3) And I didn't see big difference between EnrichDO and ORA.

Response 4: Many thanks to the reviewer for pointing this out. (1) topGO applied the Fisher’s exact test on a weighted contingency table. We thoroughly studied the algorithm and code of topGO. For the first scenario, DO terms 1, 3-5, 7-10, 13-15 obtained the same p-values as ORA, because they were leaf nodes or more significant than their child nodes, thus the p-values of these nodes remained the initial ORA p-values that calculated at the beginning. For the second scenario, DO terms 2, 6, 11, and 12 obtained p-value of 1 by topGO, because they had child nodes more significant than themselves, thus the topGO down weighted the current node by multiplying it with wd (wd=0.5) and the penalize score (1/(|(log(p(t)*p(c)))/2|+1)), so the genes were significantly down-weighted, resulting in p-value of 1.
(2) We accepted reviewer’s advice and conducted other comparisons of the significant results of three methods. The interesting gene list of Alzheimer's disease (AD) case was collected from the DisGeNET database, which was manually curated AD-related data, resulting in low p-values of three methods. We set strict thresholds with p-value < 0.01, p-adjust < 0.01, the hypergeometric test identified 859 significant terms, far exceeding those identified by EnrichDO (405) and topGO (272). We investigated top 50, 100, and 200 significant results of the three methods in the DAG. As illustrated in Supplementary Table 2, ORA’s results are more general, located at lower levels, and primarily converged on same branches. Conversely, the results of topGO are more specific, located at higher levels, and distributed across distinct branches. The above results indicated that ORA performed enrichment term-for-term, cannot solve over-enrichment problem caused by the “true-path” rule. In contrast, topGO recognized more specific terms, but at the same time, it might ignore some truly relevant nodes, such as tauopathy, and synucleinopathy in Table 3 in main manuscript. In comparison, EnrichDO represented a moderate approach that identified more specific terms as well as not neglected the true significant terms, making it more suitable for DO enrichment analysis.
Finally, when there are quite a few significant results of the statistic model, we think that not all results within the threshold are as important. For example, autosomal genetic disease (DOID:0050739) has a significant p-value and ranks 225th, 76th, and 245th in the results of EnrichDO, ORA, and topGO, respectively. However, to our knowledge, it is not associated with AD. Therefore, we believe that the ranking of terms in the results is also important, that many studies used top n terms for graphical display and even for further experimental verification.
(3) As stated in (2), the ORA cannot solve over-enrichment problem, and the results were more general, that usually be the ancestors of EnrichDO’s results. Therefore, EnrichDO is more accurate that can identify more specific diseases.
We have revised the section “Comparison with Other Methods” in main manuscript.

Page 15-16, section “Comparison with Other Methods” in the main manuscript.
“The phenomenon is more evident in other comparisons of the significant results. Setting the threshold p-value < 0.01, and p-adjust < 0.01, the hypergeometric test identified 859 significant terms, far exceeding those identified by EnrichDO (405) and topGO (272). We investigated top 50, 100, and 200 results of the three methods in the DAG. The results of the hypergeometric test are more general, located at lower levels, and primarily converged on same branches. Conversely, the results of topGO are more specific, located at higher levels, and distributed across distinct branches (Supplementary Table 2). The above results indicate that the hypergeometric test performed enrichment term-for-term, cannot solve over-enrichment problem, while topGO recognized more specific terms, but might overlook truly relevant nodes. In contrast, EnrichDO often yields more accurate, more specific, and more significant DO terms, thereby overcoming the over-enrichment problem. At the same time, it is also a moderate weighted algorithm that will not neglect true significant terms, even though their child nodes are significant.”

Point 5: ## Comparison with Current DO-based Enrichment Analysis Tools
DOSE also uses ORA, but with a different default of background. By default, DOSE uses all DO genes as the

background while not all protein-coding genes. If authors explicitly set all protein-coding genes to the "universe" argument in 'EnrichDO()", they might have very similar results as enrichDO.

Response 5: Many thanks to the reviewer for pointing this out. We accepted reviewer's advice and conducted a comparison in consistent background. Due to the background of EnrichDO was constructed by annotating all protein-coding genes (pc-genes) with DO terms, we extracted pc genes from the background data of DOSE and implemented the enrichment analysis. The new enrichment results of DOSE did not show a trend of "lower p-values", and the order of significant terms did not change obviously, as show in Supplementary Table 3.
We have revised the section "Comparison with Current DO-based Enrichment Analysis Tools" in the main manuscript.

Page 17, section "Comparison with Current DO-based Enrichment Analysis Tools" in the main manuscript.
"Another explanation for the results is that the annotation data of DOSE has not been updated and therefore contains only 10,312 annotated genes, compared to 15,106 pc genes in EnrichDO. Furthermore, DOSE obtains similar results when the enrichment background is restricted to pc genes (Supplementary Table 3)."

Point 6: ## Stability Assessment
This is not strange. The counts in ORA are always huge, which results in tiny p-values. Simply speaking, if p is the fraction of differential genes from total genes (bgRatio in https://github.com/liangcheng-hrbmu/EnrichDO/blob/devel/inst/examples/result.txt), and N is the number of genes in the gene set, as long as the observed counts is larger than pN, even with a small difference, there will be a significant p-value.

Response 6: Many thanks to the reviewer for pointing this out. We apologize for this inaccurate description. We intended to test the robustness of EnrichDO by performing data removal and noise addition test. For AD case, we randomly deleted data (or added noise) at 5% intervals, and repeated enrichment 100 times for each interval. When the disturbance of inputs was up to 30%, the number of input interesting gene set has decreased from 91 to 64, and the ratio of overlapped DO terms to original significant DO terms still remained 74.17%, which indicated that the EnrichDO is robust to data removal or noise addition. We have corrected the section "Stability Assessment" to "Robustness of EnrichDO" in main manuscript, and revised this section in main manuscript.

Page 17-18, section "Robustness of EnrichDO" in the main manuscript.
"We tested the robustness of EnrichDO with the AD dataset by removing interesting genes and by adding noise, respectively. First, we removed the AD dataset at 5% intervals and repeated the EnrichDO method 100 times for each removal. The number of significant overlapped DO terms (top 100) decreased slowly compared to the original data, and the ratio of overlapped DO terms to original significant DO terms remained at 74.17%, even after removal of up to 30% of the AD dataset (Figure 5A). These results indicate that the EnrichDO method is robust to data removal. In a similar way, noise was added to the AD dataset. We added 5% to 30% noise from a background gene set, at 5% intervals, repeated 100 times. Significantly overlapped DO terms in the top 100 decreased from 94.8 to 86.6 (Figure 5B). The results highlight the robustness of EnrichDO to data noise."

Reply to Reviewer #2:

Comments to the Author:
The manuscript by Yang et al, titled "EnrichDO: a Global Weighted Model for Disease Ontology Enrichment Analysis" presents EnrichDO, a new R package for weighted functional enrichment analysis of gene datasets with Disease Ontology (DO) terms. Enrichment analysis is a staple in current methods used in Systems Biology for the large-scale annotation of gene datasets; therefore, although a large number of tools are already available in the field, a new implementation is always welcome, provided it can offers users novel features not presented by other implementations. The authors attempt to do just that, by presenting a tool that provides weighted enrichment results from DO, using data provided by annotating

the human genome, and applying a directed acyclic graph (DAG) to take DO term inheritance into account and reduce term redundancy and biased over-representation. Such a methodology has been applied to Gene Ontology (GO enrichment) a few times before, but rarely to disease annotations; therefore, EnrichDO can become a useful tool for the community by providing this functionality.
Despite its importance, the manuscript, and the EnrichDO R package itself, need to undergo some REVISIONS, before they can be accepted for publication:

Response：Many thanks for acknowledging our efforts. We have studied comments carefully and have revised both manuscript and the R package which we hope meet with approval.

Manuscript points:

Point 1: Although the manuscript is, for the most part, well-written (clear description of the methods, distinct sections, case studies etc), the language of the text in many places is poor. The manuscript needs to be extensively proofed by a competent English speaker.

Response 1: Many thanks to the reviewer for pointing this out. To improve the clarity and readability, the manuscript has been professionally edited by the Open-Science publishing company, GigaScience Press, to correct academic and grammatical errors. All authors have also carefully revised the manuscript version to improve its overall clarity and readability. In the revised manuscript, major revisions are shown in RED color.

Point 2: Since the authors have constructed their own reference background for enrichment, by mapping the human genome with DO terms, they should add a discussion segment detailing their plans for maintaining and updating this reference dataset. Enrichment analysis tools are only as good as their background data; since new information is continuously being released, this data should always be kept up to date.

Response 2: Thank you for your invaluable comment. We accept reviewer's advice and will continuously update the background data of EnrichDO annually. Since the gene description file (generifs_basic.gz) from the GeneRIF database and the Disease ontology file (doid.obo) from the disease ontology database are both open source, it is feasible to maintain and updating the annotations with our pipeline of data processing. We have added the data update vision in the section "Discussion" and revised the section "Availability of Source Code and Requirements" in main manuscript.

Page 24, section "Discussion" in the main manuscript.
"To facilitate the use of our model, we have developed an R-based software package, which is freely available through Bioconductor (https://bioconductor.org/packages/release/bioc/html/EnrichDO.html) or at https://github.com/liangcheng-hrbmu/EnrichDO. Background annotations will be updated annually according to the latest information in the GeneRIF and DO databases, and the EnrichDO package will be continuously maintained."
Page 25, section "Availability of Source Code and Requirements" in the main manuscript.
"Project home page: https://bioconductor.org/packages/release/bioc/html/EnrichDO.html, the latest version can be available at https://www.bioconductor.org/packages/devel/bioc/html/EnrichDO.html or https://github.com/liangcheng-hrbmu/EnrichDO."

Point 3: Right now, the tool exists solely as an R package. End users, particularly wet lab scientists with little to medium bioinformatics/R knowledge (who are usually the majority of people using these enrichment tools), may find this daunting. Are there any plans to provide either a user interface (in a form of e.g. a Shiny app) or a web server version of the method? This would greatly increase the visibility and accessibility of the tool. This is in no way a request to implement such an interface for this current paper; just a recommendation for a future update.

Response 3: Thank you for your suggestion. It is also the goal of EnrichDO to serve more scientists without any biological or programming skills. To this end, we have developed a user-friendly web server, named "OntoTiger", to enabling one-stop ontology-based applications, which can be freely available at http://bio-annotation.cn/OntoTiger. EnrichDO will be integrated as the underlying algorithm in enrichment analysis

module once it was published. We will synchronous update this web service information of EnrichDO in github when the OntoTiger is released.

Tool points:

Point 1: Although the manuscript states that EnrichDO is compatible with R v. 3.5 or higher, I was unable to install it in an R 3.6.1 installation; primarily due to some of the tools' dependencies no longer being available/maintained for such old R versions. Standard Bioconductor installation was also problematic in R version 4.1; eventually, I was able to install the development version of the package through GitHub. The authors should make sure that everything can be installed as intended, and to make sure that the reported minimum requirements for R are correct.

Response 1: Many thanks to the reviewer for pointing this out. We apologize for the incorrect description of the version information. The EnrichDO package was released on Bioconductor 3.20， which is designed for R 4.4 version. Thus standard Bioconductor installation can be executed on R 4.4 and above versions. For version below R 4.4 (minimum R4.0), users can download EnrichDO and its dependency packages and install package(s) from local files. We have corrected the minimum requirements for R in section "Availability of Source Code and Requirements" in main manuscript.

Page 25, section "Availability of Source Code and Requirements" in the main manuscript.
"Programming language: It is recommended to use R version 4.4 and Bioconductor version 3.20 or later. For versions below R-4.4 (minimum R-4.0), users can download source packages from Bioconductor and install package(s) from local files."

Point 2: I tried testing the tool by following the example in the tool's vignette. The vignette example is very barebone; more detailed information should be given. The same goes for the manual, it should be more documented. Since this is an R package, a lot of people are going to want to implement EnrichDO as part of their greater analysis workflow; having a good documentation would be a great help to that.

Response 2: Thank you for your invaluable comment. We accepted reviewer's advice and rewritten the EnrichDO's vignette and manual. Users can easily implement DO enrichment analysis by following the instructions of vignette or manual, which can be obtained by the command "browseVignettes(EnrichDO)" or "??EnrichDO". We hope that our revised vignette and manual of the R package will meet with approval. The latest version can be available at https://www.bioconductor.org/packages/devel/bioc/html/EnrichDO.html or https://github.com/liangcheng-hrbmu/EnrichDO.

Point 3: The tool seems to perform very poorly in terms of speed and responsiveness. This may have to do with the fact that I used the development version instead of the Bioconductor one; however, even in that case, the performance is too poor for the specifications of the computer used for testing (8 CPU cores, 32 GB RAM). The authors should try to optimize their implementation as much as possible to improve its performance.

Response 3: Many thanks to the reviewer for pointing this out. The case studies in EnrichDO have met the performance requirements of Bioconductor, with run times under 10 seconds on Windows, under 10 seconds on macOS, and under 5 seconds on Linux System.
Additionally, EnrichDO is an iterative model that process the nodes layer by layer, resulting in slower running speed compared to the term-for-term approach (ORA methods). We have tested the AD case on different computers (minimum 4 CPU cores, 8 GB RAM), and the run times were all less than 10 seconds. It worth noting that if the input gene set intersects with many DOTerms, the running time of EnrichDO will slow down. Additionally, to prevent users from waiting for too long, EnrichDO provides feedback on the calculation progress at each layer in the console interface, allowing users to roughly be aware of the progress of the EnrichDO calculations.

Reply to Reviewer #3:

Comments to the Author:

The manuscript offers a novel method for Disease Ontology (DO) enrichment. Although the field already contains numerous tools, new tools that bring unique features are always appreciated.
Although the manuscript is generally well-organized (with clear descriptions of methods, case studies, and defined sections), many passages suffer from language issues. It would benefit from thorough proofreading by a proficient English editor.

Response：Many thanks to the reviewer for pointing this out. To improve the clarity and readability, the manuscript has been professionally edited by the Open-Science publishing company, GigaScience Press, to correct academic and grammatical errors. All authors have also carefully revised the manuscript version to improve its overall clarity and readability. In the revised manuscript, major revisions are shown in RED color.

Point 1: The authors generated a unique reference background for enrichment by mapping DO terms to the human genome, yet there is no mention of how they intend to keep this dataset current. For example, DisGeNet has already become private and is no longer accessible. Same for OMIM. What about integrating other disease-related databases (e.g., MedGen)?

Response 1: Thank you for your invaluable comment. We have carefully studied the MedGen database. It serves as a portal for information on genetic aspects of human health and disease. MedGen integrate information from various sources, but not focus on Disease Ontology, thus it is not suitable for annotations for DO enrichment.
In our work, we annotated the human genome (GeneRIF) with disease ontology (Disease Ontology) terms to generate reference background for enrichment. Since the GeneRIF and Disease Ontology database are both open source, it is feasible update the annotations with our pipeline of data processing, and we will continuously update the background data of EnrichDO annually. We have added the data update vision in the section “Discussion” and revised the section “Availability of Source Code and Requirements” in main manuscript.

Page 24, section “Discussion” in the main manuscript.
“To facilitate the use of our model, we have developed an R-based software package, which is freely available through Bioconductor (https://bioconductor.org/packages/release/bioc/html/EnrichDO.html) or at https://github.com/liangcheng-hrbmu/EnrichDO. Background annotations will be updated annually according to the latest information in the GeneRIF and DO databases, and the EnrichDO package will be continuously maintained.”
Page 25, section “Availability of Source Code and Requirements” in the main manuscript.
“Project home page: https://bioconductor.org/packages/release/bioc/html/EnrichDO.html, the latest version can be available at https://www.bioconductor.org/packages/devel/bioc/html/EnrichDO.html or https://github.com/liangcheng-hrbmu/EnrichDO. ”

Point 2: Possibility to access EnrichDO through the web? A web application would be useful.

Response 2: Thank you for your suggestion. It is also the goal of EnrichDO to serve more scientists without any biological or programming skills. To this end, we have developed a user-friendly web server, named “OntoTiger”, which dedicated to enabling one-stop ontology-based applications. EnrichDO will be integrated as the underlying algorithm in enrichment analysis module once it was published. We will synchronous update this web service information of EnrichDO in github when the OntoTiger is released. Currently, the OntoTiger can be freely available at http://bio-annotation.cn/OntoTiger.

Point 3: Many references related to functional enrichments are missing - Examples are: EnrichR, WebGestaldt, Flame, aGOtool etc.

Response 3: Many thanks to the reviewer for pointing this out. We overlooked these enrichment tools, they are all excellent enrichment web services，especially Flame (v2.0), which also supports DO enrichment analysis. We have carefully studied these tools and have added these references to the article. In addition, we have implemented enrichment analysis with Flame (v2.0) and conducted a comparison with our method. We have revised the section “Background” and added the comparison results in the section “Comparison with Current DO-based Enrichment Analysis Tools” in the main manuscript.

Page 4, section “Background” in the main manuscript.
“Similar web service tools include EnrichR [27], WebGestaldt [28], Flame (V2.0) [29], and aGOtool [30], all of which provide disease enrichment analysis by integrating multiple disease data sources.”
Page 17, section “Comparison with Current DO-based Enrichment Analysis Tools” in the main manuscript.
“Flame had three overlaps with EnrichDO: “AD,” “dementia,” and “cognitive disorder.” Flame recognized AD as the top correlated disease; however, in comparison to EnrichDO, other results obtained by Flame were more general, as shown in Figure 4. In addition, the branches of Flame results are more similar to DOSE (with six overlaps), and there is a branch related to metabolic diseases.”

Point 4: The tool's performance seemed suboptimal, showing slow processing times and low responsiveness. The authors should consider optimizing the package to enhance its speed and responsiveness.

Response 4: Many thanks to the reviewer for pointing this out. The case studies in EnrichDO have met the performance requirements set by Bioconductor, with run times under 10 seconds on Windows, under 10 seconds on macOS, and under 5 seconds on Linux System.
Additionally, EnrichDO is an iterative model that process the nodes layer by layer, resulting in slower running speed compared to the term-for-term approach (ORA methods). We have tested the AD case on different computers (minimum 4 CPU cores, 8 GB RAM), and the run times were all less than 10 seconds. It worth noting that if the input gene set intersects with many DOTerms, the running time of EnrichDO will slow down. Additionally, to prevent users from waiting for too long, EnrichDO provides feedback on the calculation progress at each layer in the console interface, allowing users to roughly be aware of the progress of the EnrichDO calculations.
