# Supplementary material for: EnrichDO: a global weighted model for Disease Ontology enrichment analysis
Source: Gigascience. 2025 Mar 26;14:giaf021. doi: 10.1093/gigascience/giaf021 (PMC11945307; doi:10.1093/gigascience/giaf021)
Supplement: giaf021_Response_to_Reviewer_Comments_Revision_1 [file giaf021_response_to_reviewer_comments_revision_1.pdf]

Author's Response To Reviewer Comments

Dear Prof. Nicole Nogoy and reviewers,
Thanks very much for your editorial efforts regarding our manuscript entitled "EnrichDO: a Global Weighted Model for Disease Ontology Enrichment Analysis" (Manuscript ID: GIGA-D-24-00357R1). We also thank the reviewers for their constructive comments to strengthen this manuscript. Those comments are all valuable and very helpful for revising and improving our paper, as well as the important guiding significance to our research. We have studied comments carefully and have made corrections which we hope meet with approval. Revised portion are marked in RED in the paper. The main corrections in the paper and the responses to the reviewer's comments are as following:

Reply to Editor
In addition, please register any new software application in the bio.tools and SciCrunch.org databases to receive RRID (Research Resource Identification Initiative ID) and biotoolsID identifiers, and include these in your manuscript. Computational workflows should be registered in workflowhub.eu and the DOIs cited in the relevant places in the manuscript. These will facilitate tracking, reproducibility and re-use of your tool.

Response：Thank you very much for your editorial efforts regarding our manuscript. In response to your request, we have registered our software on the bio.tools and SciCrunch.org databases and added RRID and biotoolsID identifiers to the section "Availability of Source Code and Requirements" in main manuscript. We have also registered the computational workflows in workflowhub.eu and added the DOIs to the section "Availability of Source Code and Requirements" in main manuscript.
We have provided detailed responses to the reviewers' comments. We confirmed that the modifications and additional experimental results have been included in the main manuscript and supplementary data as required. In addition, we have adjusted the overall style of the manuscript to ensure compliance with the journal's "Author Guidelines" section. We believe that the manuscript has been greatly strengthened by the critique of the reviewers and hope that both you and the reviewers will now find the paper suitable for publication.

Reply to Reviewer #1
Comments to the Author:

Point 1: The algorithm in the manuscript is very similar as Alexa 2006. What is the major improvement compared to Alexa 2006? Supporting the analysis on DO does not account for an improvement.

Response 1: Many thanks to the reviewer for carefully reviewing our work. Both GO and DO possess hierarchical structures that follow the "true-path" rule. Therefore, many algorithms based on GO are applicable to DO, and we referenced the algorithmic ideas of weight method from Alexa 2006 (topGO) when calculating the dynamic weight wd. However, there are some differences between topGO and EnrichDO, which we will highlight from the following aspects:
(1) The biological significance of GO and DO is different. GO is a gene classification system where the most specific terms on each ontology branch are typically interpreted as the optimal classification for the genes-of-interest list. topGO is designed to detect the locally most significant terms and typically returned more specific terms within each ontology branch. In comparison, the parent and child nodes in DO often share common features and potential mechanisms that it is inappropriate to focus solely on the most specific nodes in DO enrichment. Moreover, the current DO-based enrichment analysis are all term-for-term methods that cannot solve the over-enrichment problem. Therefore, we propose the EnrichDO method.
(2) The purpose and design concepts of topGO and EnrichDO diverge significantly. topGO is tailored to detect the locally most significant terms of connected nodes (a parent and its child) by down-weighting genes in less significant neighbors. Conversely, EnrichDO aims at addressing the over-enrichment problems that arises due to the "true-path" rule by employing a double-weighted model, supplemented with a penalty score.
(3) The major improvement of algorithm for EnrichDO are list as follows: (i) Setting distinct initial weight wi. Due to the over-enrichment problems being caused by the "true-path" rule, we assign distinct initial weights to directly annotated genes and to indirectly annotated genes caused by the "true-path" rule,

respectively, to reinforce the saliency of direct gene-DO annotations and to reduce the influence of the indirect annotated genes. For each DO term, the weights for all directly annotated genes are set to 1, and the weights for indirectly annotated genes decrease by 0.1 for each level inherited upward. The initial weights are range from 0.5 to 1. Decreasing the initial weights of the indirectly annotated genes, which are typically annotated with parent or ancestor nodes, can effectively alleviate the over-enrichment problem. (ii) The dynamic weights are different. In topGO, the dynamic weights w was set to log(score(v))/log(score(u)) or score(u)/score(v), where v and u represent the child and the parent, respectively. However, there existed a case that u and v obtaining significant and similar p-values (|score(u)-score(v)|<1e-50), which was deemed as the inheritance problem stem from the"true-path" rule. For this case, w was set to 2 in the code of the topGO algorithm, and the current node was significantly down weighted by w and the penalize score, resulting in p-value of 1. In contrast, EnrichDO has addressed the inheritance problem by reducing the initial weights of the indirectly annotated genes to weaken the influence of inheritance, and utilizing the dynamic weights to reduce the local impact of inheritance. (iii) Setting the penalty score. The penalty score is used to supplement the double-weighting of initial weights and dynamic weights. It is specifically designed for such a situation that the parent and the child have similar p-values but with low significance. In this case, the penalty score, rather than the dynamic weight, can significantly reduce the gene weights, consequently filtering out less significant terms. Overall, topGO is a rigorous weight algorithm that can detect more specific terms that located at higher levels and is suitable for GO; while EnrichDO is a moderate approach that can overcome the over-enrichment problems as well as not neglect the true significant terms, which is suitable for DO enrichment analysis. Furthermore, (iv) Setting a threshold for the initial p-values before the iteration process. Only nodes that meet the threshold (p-value<0.01) participate in the dynamic weight reduction and the iteration process. This leads to significant improvement in running speed. The average running speeds (5 times for each case) of EnrichDO and topGO are as follows: in the ALL case, the speeds are 30.09s and 58.43s; in the Alzheimer's case, the speeds are 40.05s and 72.12s; and in the PAAD (GSE16515) case, the speeds are 47.94s and 92.64s.
We have revised the section "Comparison with Other Methods" in main manuscript.

Page 8-9, section "Weighted DO Enrichment Analysis" in the main manuscript.
"To address this problem, we developed the weighted DO enrichment analysis method, referred to as EnrichDO, by double-weighting the annotated genes and integrating DO graph topology on a global scale. On the one hand, we assigned distinct initial weights to directly annotated genes and to indirectly annotated genes caused by the "true-path" rule, respectively, with the intention of reinforcing the saliency of direct gene-DO annotations while reducing the influence of indirectly annotated genes. On the other hand, we dynamically down-weighted genes in less significant nodes to reinforce differences in significance between the parent and its children. as described by Alexa et al. [33]."

Point 2: The "weighted algorithm" introduced in Alexa 2006 and which this manuscript is based on, is implemented in the topGO package via "runTest(…, algorithm="weighted"). In the Results section where authors compared their tool to topGO, what is the method they used for running topGO? The default (which is Fisher's exact test) or "weighted"? Please specify the parameters they used for other tools in the comparisons.

Response 2: Many thanks to the reviewer for pointing this out. We apologize for the lack of parameters description of topGO in the section "Comparison with Other Methods". In this section, we utilized the "weight" algorithm of topGO via the runTest function (topGOdata, algorithm = "weight", statistic = "fisher"), which applies Fisher's exact test on a weighted contingency table. We have revised the section "Comparison with Other Methods" in main manuscript.

Page 14, section "Comparison with Other Methods" in the main manuscript.
"We compared EnrichDO with the classic overrepresentation analysis method (common hypergeometric test) and topGO [33], both of which have been widely used in GO enrichment analysis. Consequently, we conducted comparisons based on GO and DO separately, utilizing the latest annotations of GO and DO. For the topGO analysis, the parameters for the algorithm and statistic were set to "weight" and "fisher", respectively."

Point 3: Make sure the notations are consistent. In Equation 2 and 3, use either log or lg.

Response 3: Many thanks to the reviewer for pointing this out. We have revised lg in Equation 3 to log in the main manuscript.

Point 4: Equation 3 is still not convincing. How does the factor 1/10 and lg(xmin) come to the equation? What is their meaning? Why do we need a second penalty? Is this penalty necessary? To my understanding, the aim is to down-weight genes to let the corresponding term become insignificant and I think w_d can already do the job.

Response 4: Many thanks to the reviewer for pointing this out. The penalty score is used to supplement the initial weight and dynamic weight. EnrichDO first assigns distinct initial weights to reduce the influence of the indirect annotated genes, then it adopts dynamic weight to significantly reduce gene weights when there is a significant difference between parent and child nodes. However, when the parent and the child nodes have similar p-values, the value of dynamic weight (wd) is approximately 1, cannot significantly reduce the weight. In this case, the penalty score, rather than the dynamic weight, can significantly reduce the gene weights, consequently filtering out less significant terms.
The factor 1/10 and log(xmin) represents the parameters of penalty score. As the p-value ranges from 0 to 1, xmin denotes the minimum positive number in R language, is used to make the degree of down-weight be inversely proportional to significance of the nodes, as well as controlling the range of the weights. Nodes with higher significance are usually significantly correlated with the genes-of-interest list, so they will be slightly or not down-weighted. While nodes with lower significance are usually located in lower levels and are not so correlated with the genes-of-interest list, so they will be significantly down-weighted. 1/10 is the scale factor, which is defined based on large number of experiments. We tested the scale factor from 1/2, 1/3, to 1/10, 1/20, and found that 1/10 is the most appropriate scale factor. When the scale factor is set to 1/10, the maximum degree of weight reduction is 30.76, and the penalty score will be 1 if the product of the p-values of the parent and child is less than 1e-30.8; When the scale factor is set to 1/2, the maximum degree of weight reduction is 153.82, and the penalty score will be 1 if the product of the p-values of the parent and child is less than 1e-153.82; When the scale factor is set to 1/20, the degree of weight reduction is quite small that making the penalty score meaningless. Overall, setting the above penalty score parameters can better control the degree of weight reduction.
In Alzheimer's disease (AD) case, the primary immunodeficiency disease (DOID: 612, level=4) is the parent node of the autoimmune disease (DOID: 417, level=5), and their significance are relatively low. After applying the penalty score, the p-value of the primary immunodeficiency disease increased from 3.87e-06 to 1, and the p-value of the autoimmune disease increased from 3.98e-09 to 4.06e-04. In another group, tauopathy (DOID: 680, level=6, p=4.3e-63) is the parent node of AD (DOID: 10652, level=7, p=2.19e-71). Both of these nodes possess high significance, and the p-values remain unchanged after applying the penalty score. In another case of the hallmark gene set of “pancreas beta cells”, the numbers of significant nodes without penalty score and with penalty score are 94 and 63, respectively. The 31 missing nodes are located in lower levels that close to the root node, and the annotated genes are almost same to their children nodes. Therefore, the penalty function can further alleviate the over-enrichment problems.
Additionally, in EnrichDO, the parameter “penalty” is optional; however, we recommend using it based on extensive experimental results.

Point 5: The plots are in bad resolution. In Figure 1, please only capitalize the first word in each line.

Response 5: Many thanks to the reviewer for pointing this out. We have revised Figure 1 according to the reviewer’s advice, and resubmitted the PDF files of all Figures according to the “authors’ instructions.”
