# Supplementary material for: EnrichDO: a global weighted model for Disease Ontology enrichment analysis
Source: Gigascience. 2025 Mar 26;14:giaf021. doi: 10.1093/gigascience/giaf021 (PMC11945307; doi:10.1093/gigascience/giaf021)
Supplement: giaf021_Response_to_Reviewer_Comments_Revision_2 [file giaf021_response_to_reviewer_comments_revision_2.pdf]

Author's Response To Reviewer Comments

Dear Nicole Nogoy, Ph.D,
Thank you very much for your editorial efforts regarding our manuscript entitled “EnrichDO: a Global Weighted Model for Disease Ontology Enrichment Analysis” (GIGA-D-24-00357R2). We have carried out all essential formatting revisions suggested by the editors and added the references to the manuscript. Revised portion is marked in RED in the paper. We hope that you will now find the paper suitable for publication.
