# Supplementary material for: EnrichDO: a global weighted model for Disease Ontology enrichment analysis
Source: Gigascience. 2025 Mar 26;14:giaf021. doi: 10.1093/gigascience/giaf021 (PMC11945307; doi:10.1093/gigascience/giaf021)
Supplement: giaf021_Reviewer_1_Report_Original_Submission — Zuguang Gu -- 10/24/2024 [file giaf021_reviewer_1_report_original_submission.pdf]

Reviewer Report

Title: EnrichDO: a Global Weighted Model for Disease Ontology Enrichment Analysis

Version: Original Submission **Date:** 10/24/2024

Reviewer name: Zuguang Gu

Reviewer Comments to Author:

In the manuscript entitled "EnrichDO: a Global Weighted Model for Disease Ontology Enrichment Analysis", authors described a new method and a tool for DO enrichment analysis by considering the topology of DO. However, my comment is the manuscript is not clearly written, the methods are not clearly explained and the analysis is not convincing.## The languageI am very flexible to the English writing when I review manuscripts because I am not a native English speaker either. But in this manuscript, authors should spend more time to improve their English. There are many mistakes and unnatural uses of words and grammar. For example, in the Abstract, "DO-based methods were relatively scare,", scare? I guess it should be "rare"?## Annotating the Human Genome with DO TermsAuthors link DO terms to genes by using an ontology annotator from gene-related textual descriptions. Have authors validated the quality of such mapping? In the manuscript, authors claimed that their annotation is better because there are more genes annotated to DO terms. There might be false positives in the mapping. The validation can be easily done by manually checking e.g. 1000 mappings, which, according to my experience, only takes 1-2 hours' work.## Weighted DO Enrichment AnalysisShouldn't step iv be the the second step because it is actually related to how to calculate score(c) and score(t)?I don't understand equation 3, and its functions in the adjustment process in this method.How does it deal with nodes with multiple parents?## Comparison with Other MethodstopGO by default also uses ORA, so it is actually the same as ORA in the comparison. Authors can see it in Table 3 where ORA and topGO give the same p-values.I noticed DO terms 2, 6, 11 and 12 have p-value of 1 by topGO. What is the reason for that? Is it because topGO has a different cutoff for minimal number of genes in a gene set?Only compare top 10 terms is not sufficient to conclude which tool is better than others. As authors can see in the tables, these top significant gene sets are with very small p-values. Under this context, the ranking of gene sets is less important because they are "all significant and important".And I didn't see big difference between EnrichDO and ORA.## Comparison with Current DO-based Enrichment Analysis ToolsDOSE also uses ORA, but with a different default of background. By default, DOSE uses all DO genes as the background while not all protein-coding genes. If authors explicitly set all protein-coding genes to the "universe" argument in 'EnrichDO()", they might have very similar results as enrichDO.## Stability AssessmentThis is not strange. The counts in ORA are always huge, which results in tiny p-values. Simply speaking, if p is the fraction of differential genes from total genes (bgRatio in https://github.com/liangcheng-hrbmu/EnrichDO/blob/devel/inst/examples/result.txt), and N is the number of genes in the gene set, as long as the observed counts is larger than pN, even with a small difference, there will be a significant p-value.

Level of Interest

Please indicate how interesting you found the manuscript: Choose an item.

Quality of Written English

Please indicate the quality of language in the manuscript: Choose an item.

Declaration of Competing Interests

Please complete a declaration of competing interests, considering the following questions:

- Have you in the past five years received reimbursements, fees, funding, or salary from an organisation that may in any way gain or lose financially from the publication of this manuscript, either now or in the future?
- Do you hold any stocks or shares in an organisation that may in any way gain or lose financially from the publication of this manuscript, either now or in the future?
- Do you hold or are you currently applying for any patents relating to the content of the manuscript?
- Have you received reimbursements, fees, funding, or salary from an organization that holds or has applied for patents relating to the content of the manuscript?
- Do you have any other financial competing interests?
- Do you have any non-financial competing interests in relation to this paper?

If you can answer no to all of the above, write 'I declare that I have no competing interests' below. If your reply is yes to any, please give details below.

I declare that I have no competing interests

I agree to the open peer review policy of the journal. I understand that my name will be included on my report to the authors and, if the manuscript is accepted for publication, my named report including any attachments I upload will be posted on the website along with the authors' responses. I agree for my report to be made available under an Open Access Creative Commons CC-BY license (http://creativecommons.org/licenses/by/4.0/). I understand that any comments which I do not wish to be included in my named report can be included as confidential comments to the editors, which will not be published.

Choose an item.

To further support our reviewers, we have joined with Publons, where you can gain additional credit to further highlight your hard work (see: https://publons.com/journal/530/gigascience). On publication of this paper, your review will be automatically added to Publons, you can then choose whether or not to claim your Publons credit. I understand this statement.

Yes Choose an item.
