# Supplementary material for: EnrichDO: a global weighted model for Disease Ontology enrichment analysis
Source: Gigascience. 2025 Mar 26;14:giaf021. doi: 10.1093/gigascience/giaf021 (PMC11945307; doi:10.1093/gigascience/giaf021)
Supplement: giaf021_Reviewer_1_Report_Revision_1 — Zuguang Gu -- 1/7/2025 [file giaf021_reviewer_1_report_revision_1.pdf]

Reviewer Report

Title: EnrichDO: a Global Weighted Model for Disease Ontology Enrichment Analysis

Version: Revision 1 **Date:** 1/7/2025

Reviewer name: Zuguang Gu

Reviewer Comments to Author:

I have read the paper Alexa 2006 and the topGO package. I have the following question:1. The algorithm in the manuscript is very similar as Alexa 2006. What is the major improvement compared to Alexa 2006? Supporting the analysis on DO does not account for an improvement.2. The "weighted algorithm" introduced in Alexa 2006 and which this manuscript is based on, is implemented in the topGO package via "runTest(…, algorithm="weighted"). In the Results section where authors compared their tool to topGO, what is the method they used for running topGO? The default (which is Fisher's exact test) or "weighted"? Please specify the parameters they used for other tools in the comparisons.3. Make sure the notations are consistent. In Equation 2 and 3, use either log or lg.4. Equation 3 is still not convincing. How does the factor 1/10 and lg(xmin) come to the equation? What is their meaning? Why do we need a second penalty? Is this penalty necessary? To my understanding, the aim is to down-weight genes to let the corresponding term become insignificant and I think w_d can already do the job.5. The plots are in bad resolution. In Figure 1, please only capitalize the first word in each line.

Level of Interest

Please indicate how interesting you found the manuscript: Choose an item.

Quality of Written English

Please indicate the quality of language in the manuscript: Choose an item.

Declaration of Competing Interests

Please complete a declaration of competing interests, considering the following questions:

- Have you in the past five years received reimbursements, fees, funding, or salary from an organisation that may in any way gain or lose financially from the publication of this manuscript, either now or in the future?
- Do you hold any stocks or shares in an organisation that may in any way gain or lose financially from the publication of this manuscript, either now or in the future?
- Do you hold or are you currently applying for any patents relating to the content of the manuscript?
- Have you received reimbursements, fees, funding, or salary from an organization that holds or has applied for patents relating to the content of the manuscript?

- Do you have any other financial competing interests?
- Do you have any non-financial competing interests in relation to this paper?

If you can answer no to all of the above, write 'I declare that I have no competing interests' below. If your reply is yes to any, please give details below.

I declare that I have no competing interests.

I agree to the open peer review policy of the journal. I understand that my name will be included on my report to the authors and, if the manuscript is accepted for publication, my named report including any attachments I upload will be posted on the website along with the authors' responses. I agree for my report to be made available under an Open Access Creative Commons CC-BY license (http://creativecommons.org/licenses/by/4.0/). I understand that any comments which I do not wish to be included in my named report can be included as confidential comments to the editors, which will not be published.

Choose an item.

To further support our reviewers, we have joined with Publons, where you can gain additional credit to further highlight your hard work (see: https://publons.com/journal/530/gigascience). On publication of this paper, your review will be automatically added to Publons, you can then choose whether or not to claim your Publons credit. I understand this statement.

Yes Choose an item.
