# Supplementary material for: EnrichDO: a global weighted model for Disease Ontology enrichment analysis
Source: Gigascience. 2025 Mar 26;14:giaf021. doi: 10.1093/gigascience/giaf021 (PMC11945307; doi:10.1093/gigascience/giaf021)
Supplement: giaf021_Reviewer_2_Report_Original_Submission — Fotis A Baltoumas, PhD -- 11/20/2024 [file giaf021_reviewer_2_report_original_submission.pdf]

Reviewer Report

Title: EnrichDO: a Global Weighted Model for Disease Ontology Enrichment Analysis

Version: Original Submission **Date:** 10/29/2024

Reviewer name: Fotis A Baltoumas, PhD

Reviewer Comments to Author:

The manuscript by Yang et al, titled "EnrichDO: a Global Weighted Model for Disease Ontology Enrichment Analysis" presents EnrichDO, a new R package for weighted functional enrichment analysis of gene datasets with Disease Ontology (DO) terms. Enrichment analysis is a staple in current methods used in Systems Biology for the large-scale annotation of gene datasets; therefore, although a large number of tools are already available in the field, a new implementation is always welcome, provided it can offers users novel features not presented by other implementations. The authors attempt to do just that, by presenting a tool that provides weighted enrichment results from DO, using data provided by annotating the human genome, and applying a directed acyclic graph (DAG) to take DO term inheritance into account and reduce term redundancy and biased over-representation. Such a methodology has been applied to Gene Ontology (GO enrichment) a few times before, but rarely to disease annotations; therefore, EnrichDO can become a useful tool for the community by providing this functionality.Despite its importance, the manuscript, and the EnrichDO R package itself, need to undergo some REVISIONS, before they can be accepted for publication:Manuscript points:1. Although the manuscript is, for the most part, well-written (clear description of the methods, distinct sections, case studies etc), the language of the text in many places is poor. The manuscript needs to be extensively proofed by a competent English speaker.2. Since the authors have constructed their own reference background for enrichment, by mapping the human genome with DO terms, they should add a discussion segment detailing their plans for maintaining and updating this reference dataset. Enrichment analysis tools are only as good as their background data; since new information is continuously being released, this data should always be kept up to date.3. Right now, the tool exists solely as an R package. End users, particularly wet lab scientists with little to medium bioinformatics/R knowledge (who are usually the majority of people using these enrichment tools), may find this daunting. Are there any plans to provide either a user interface (in a form of e.g. a Shiny app) or a web server version of the method? This would greatly increase the visibility and accessibility of the tool. This is in no way a request to implement such an interface for this current paper; just a recommendation for a future update.Tool points:1. Although the manuscript states that EnrichDO is compatible with R v. 3.5 or higher, I was unable to install it in an R 3.6.1 installation; primarily due to some of the tools' dependencies no longer being available/maintained for such old R versions. Standard Bioconductor installation was also problematic in R version 4.1; eventually, I was able to install the development version of the package through GitHub. The authors should make sure that everything can be installed as intended, and to make sure that the reported minimum requirements for R are correct.2. I tried testing the tool by following the example in the tool's vignette. The vignette example is very barebone; more detailed information should be given. The same goes for the manual, it should be more documented. Since this is an R package, a lot of people

are going to want to implement EnrichDO as part of their greater analysis workflow; having a good documentation would be a great help to that.3. The tool seems to perform very poorly in terms of speed and responsiveness. This may have to do with the fact that I used the development version instead of the Bioconductor one; however, even in that case, the performance is too poor for the specifications of the computer used for testing (8 CPU cores, 32 GB RAM). The authors should try to optimize their implementation as much as possible to improve its performance.

Level of Interest

Please indicate how interesting you found the manuscript: Choose an item.

Quality of Written English

Please indicate the quality of language in the manuscript: Choose an item.

Declaration of Competing Interests

Please complete a declaration of competing interests, considering the following questions:

- Have you in the past five years received reimbursements, fees, funding, or salary from an organisation that may in any way gain or lose financially from the publication of this manuscript, either now or in the future?
- Do you hold any stocks or shares in an organisation that may in any way gain or lose financially from the publication of this manuscript, either now or in the future?
- Do you hold or are you currently applying for any patents relating to the content of the manuscript?
- Have you received reimbursements, fees, funding, or salary from an organization that holds or has applied for patents relating to the content of the manuscript?
- Do you have any other financial competing interests?
- Do you have any non-financial competing interests in relation to this paper?

If you can answer no to all of the above, write 'I declare that I have no competing interests' below. If your reply is yes to any, please give details below.

I declare that I have no competing interests

I agree to the open peer review policy of the journal. I understand that my name will be included on my report to the authors and, if the manuscript is accepted for publication, my named report including any attachments I upload will be posted on the website along with the authors' responses. I agree for my report to be made available under an Open Access Creative Commons CC-BY license (http://creativecommons.org/licenses/by/4.0/). I understand that any comments which I do not wish to

be included in my named report can be included as confidential comments to the editors, which will not be published.

Choose an item.

To further support our reviewers, we have joined with Publons, where you can gain additional credit to further highlight your hard work (see: https://publons.com/journal/530/gigascience). On publication of this paper, your review will be automatically added to Publons, you can then choose whether or not to claim your Publons credit. I understand this statement.

Yes Choose an item.
