# Supplementary material for: EnrichDO: a global weighted model for Disease Ontology enrichment analysis
Source: Gigascience. 2025 Mar 26;14:giaf021. doi: 10.1093/gigascience/giaf021 (PMC11945307; doi:10.1093/gigascience/giaf021)
Supplement: giaf021_Reviewer_3_Report_Original_Submission — Georgios Pavlopoulos -- 11/4/2024 [file giaf021_reviewer_3_report_original_submission.pdf]

Reviewer Report

Title: EnrichDO: a Global Weighted Model for Disease Ontology Enrichment Analysis

Version: Original Submission **Date:** 11/4/2024

Reviewer name: Georgios Pavlopoulos

Reviewer Comments to Author:

The manuscript offers a novel method for Disease Ontology (DO) enrichment. Although the field already contains numerous tools, new tools that bring unique features are always appreciated.Although the manuscript is generally well-organized (with clear descriptions of methods, case studies, and defined sections), many passages suffer from language issues. It would benefit from thorough proofreading by a proficient English editor.1. The authors generated a unique reference background for enrichment by mapping DO terms to the human genome, yet there is no mention of how they intend to keep this dataset current. For example, DisGeNet has already become private and is no longer accessible. Same for OMIM. What about integrating other disease-related databases (e.g., MedGen)?2. Possibility to access EnrichDO through the web? A web application would be useful.3. Many references related to functional enrichments are missing - Examples are: EnrichR, WebGestaldt, Flame, aGOtool etc..4. The tool's performance seemed suboptimal, showing slow processing times and low responsiveness. The authors should consider optimizing the package to enhance its speed and responsiveness.

Level of Interest

Please indicate how interesting you found the manuscript: Choose an item.

Quality of Written English

Please indicate the quality of language in the manuscript: Choose an item.

Declaration of Competing Interests

Please complete a declaration of competing interests, considering the following questions:

- Have you in the past five years received reimbursements, fees, funding, or salary from an organisation that may in any way gain or lose financially from the publication of this manuscript, either now or in the future?
- Do you hold any stocks or shares in an organisation that may in any way gain or lose financially from the publication of this manuscript, either now or in the future?
- Do you hold or are you currently applying for any patents relating to the content of the manuscript?
- Have you received reimbursements, fees, funding, or salary from an organization that holds or has applied for patents relating to the content of the manuscript?

- Do you have any other financial competing interests?
- Do you have any non-financial competing interests in relation to this paper?

If you can answer no to all of the above, write 'I declare that I have no competing interests' below. If your reply is yes to any, please give details below.

No competing interests

I agree to the open peer review policy of the journal. I understand that my name will be included on my report to the authors and, if the manuscript is accepted for publication, my named report including any attachments I upload will be posted on the website along with the authors' responses. I agree for my report to be made available under an Open Access Creative Commons CC-BY license (http://creativecommons.org/licenses/by/4.0/). I understand that any comments which I do not wish to be included in my named report can be included as confidential comments to the editors, which will not be published.

Choose an item.

To further support our reviewers, we have joined with Publons, where you can gain additional credit to further highlight your hard work (see: https://publons.com/journal/530/gigascience). On publication of this paper, your review will be automatically added to Publons, you can then choose whether or not to claim your Publons credit. I understand this statement.

Yes Choose an item.
